# Rupture strength of living cell monolayers

**DOI:** 10.1038/s41563-024-02027-3

**Published:** 2024-10-28

**Authors:** Julia Duque, Alessandra Bonfanti, Jonathan Fouchard, Lucia Baldauf, Sara R. Azenha, Emma Ferber, Andrew Harris, Elias H. Barriga, Alexandre J. Kabla, Guillaume Charras

**Affiliations:** 1 London Centre for Nanotechnology, University College London, London, UK; 2 Department of Civil and Environmental Engineering, Politecnico di Milano, Milan, Italy; 3 Laboratoire de Biologie du Développement (LBD), Institut de Biologie Paris Seine (IBPS), Paris, France; 4 Gulbenkian Institute of Science (IGC), Oeiras, Portugal; 5 Mechanical and Aerospace Engineering, Carleton University, Ottawa, Ontario Canada; 6 Cluster of Excellence Physics of Life, TU Dresden, Dresden, Germany; 7 Department of Engineering, University of Cambridge, Cambridge, UK; 8 Institute for the Physics of Living Systems, University College London, London, UK; 9 Department of Cell and Developmental Biology, University College London, London, UK

**Keywords:** Biopolymers in vivo, Rheology

## Abstract

To fulfil their function, epithelial tissues need to sustain mechanical stresses and avoid rupture. Although rupture is usually undesired, it is central to some developmental processes, for example, blastocoel formation. Nonetheless, little is known about tissue rupture because it is a multiscale phenomenon that necessitates comprehension of the interplay between mechanical forces and biological processes at the molecular and cellular scales. Here we characterize rupture in epithelial monolayers using mechanical measurements, live imaging and computational modelling. We show that despite consisting of only a single layer of cells, monolayers can withstand surprisingly large deformations, often accommodating several-fold increases in their length before rupture. At large deformation, epithelia increase their stiffness multiple fold in a process controlled by a supracellular network of keratin filaments. Perturbing the keratin network organization fragilized the monolayers and prevented strain-stiffening. Although the kinetics of adhesive bond rupture ultimately control tissue strength, tissue rheology and the history of deformation set the strain and stress at the onset of fracture.

## Abstract (web summary)

Tissue monolayers avoid rupture at large tensile stresses through a strain-stiffening process governed by intermediate keratin filaments.

## Main

During development and normal physiological function, epithelial monolayers continuously withstand mechanical stresses. In embryogenesis, tissues undergo large deformations over hours or days, in processes enabled by the rearrangement of adhesive contacts at the cellular scale. In contrast, deformations in adult tissues are typically smaller and take place on shorter timescales with fixed tissue organization. For example, lung alveoli deform by ~20% up to 20 times a minute during breathing^1^, and the skin deforms by over 50% in fractions of a second during limb movement^2^. The mechanical role of epithelia is particularly apparent in disease. Mutations in intermediate filaments and desmosomal proteins in the epidermis lead to epidermolysis bullosa, a family of diseases characterized by fragile skin that fractures in response to physiological levels of deformation^3^. However, we know relatively little about the strength of epithelia and the biological structures that underpin it.

Fracture is a permanent break of a material into smaller components when subjected to stress. Loss of material integrity can occur once a threshold of strain or stress is exceeded and the mode of fracture often depends on the rate of stress application. Although much is known about fracture in hard materials such as ceramics and steel^4^, fracture in soft materials is less well understood^5,6^. Tissue fracture is inherently a multiscale process with deformations applied at the tissue scale resulting in stress at the cellular scale that causes the rupture of intercellular adhesion complexes at the molecular scale. Further complexity arises because of the viscoelastic properties of living tissues that stem from biological processes with distinct timescales, ranging from seconds to days. In living tissues, rupture can occur in response to either extrinsically applied forces or forces generated by the cells within a tissue. Although we are familiar with the former, the latter is unique to living tissues. Indeed, tissues can self-rupture as a consequence of the local upregulation of active stress due to myosin contractility, motility on a substrate or an increase in osmotic forces to ensure the proper development of embryonic structures^7–11^.

Here we investigate rupture at the tissue scale by subjecting monolayers devoid of a substrate to ramps in deformation. We reveal that epithelia strain-stiffen at large deformation due to the emergent properties of a supracellular network of keratin filaments and show that tissue-scale rheology interplays with molecular-bond rupture kinetics to govern tissue rupture.

## Monolayers withstand large deformations before rupture

**Fig. 1:**
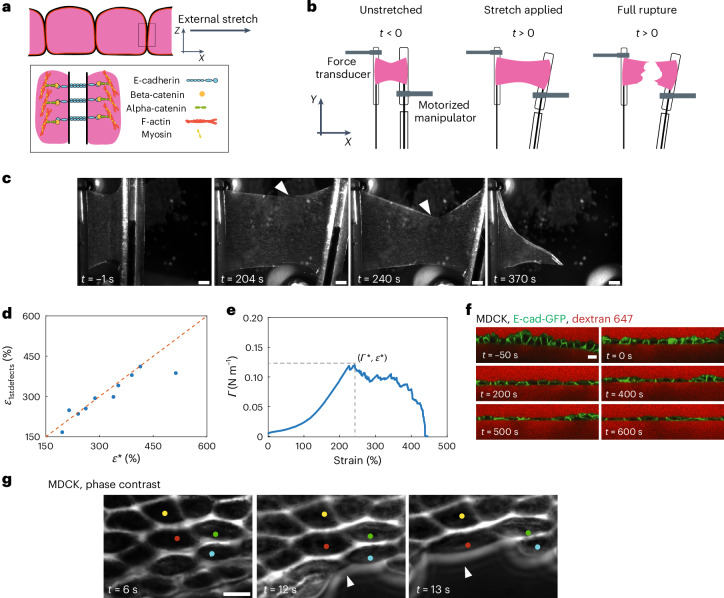
Epithelial monolayers rupture in response to excessive stretch. **a**, Cellular-scale diagram of the epithelial monolayer. Top: profile view of the monolayer. Cells are linked to one another via specialized junctions. Bottom: zoomed-in view of an adherens junctions linking the F-actin cytoskeleton of neighbouring cells. The ectodomain of E-cadherin links cells to one another, whereas its intracellular domain binds to the F-actin cytoskeleton via beta- and alpha-catenin. Myosin motor proteins bind F-actin to generate a cellular surface tension that results in a pre-tension in the monolayer. **b**, Diagram of the experiment. The monolayers in pink are subjected to a ramp in deformation applied via displacement of one of the test rods. Stretch starts at time 0 and continues at a constant rate until full rupture of the monolayer. **c**, Bright-field microscopy time series of an MDCK monolayer subjected to a ramp in deformation performed at 1% s^−1^. Arrowheads indicate the crack tip. Time is indicated in the bottom-left corner. Scale bars, 500 μm. **d**, Strain at which the first defects (*ε*_1stdefects_) are observed as a function of strain ε* at which the maximum tension is reached. The dashed red line shows the line of slope 1. **e**, Evolution of monolayer tension as a function of applied strain for the monolayer shown in **c**. The dashed lines show the maximum tension Γ* and strain ε* coinciding with the appearance of the first defects. Full rupture of the monolayer takes place for *ε* = 450%. **f**, Time series of a profile view of an MDCK monolayer during stretch. Intercellular junctions are visualized with E-cadherin-GFP (green) and cells are visualized by dye exclusion of dextran-Alexa 647 (red) added to the medium. Time is indicated in the bottom-left corner of each image. Deformation starts at time 0 and proceeds at a rate of 1% s^–1^. Scale bar, 10 μm. **g**, High-magnification phase contrast time series of crack propagation along cellular interfaces in an MDCK monolayer. The crack front is indicated by the white arrowheads. Several cells are marked by coloured dots in each frame. Time is indicated in the bottom-left corner. Scale bar, 10 μm. Source data

To investigate the response of living tissues to externally applied deformation, we used Madin–Darby canine kidney (MDCK) monolayers devoid of a substrate and suspended between two test rods^12^. In these conditions, all of the force applied to the monolayer is borne by intercellular adhesions and transmitted through the cytoskeleton (Fig. 1a), making this an ideal system to explore the operating limits of a living cellularized material.

**Extended Data Fig. 1:**
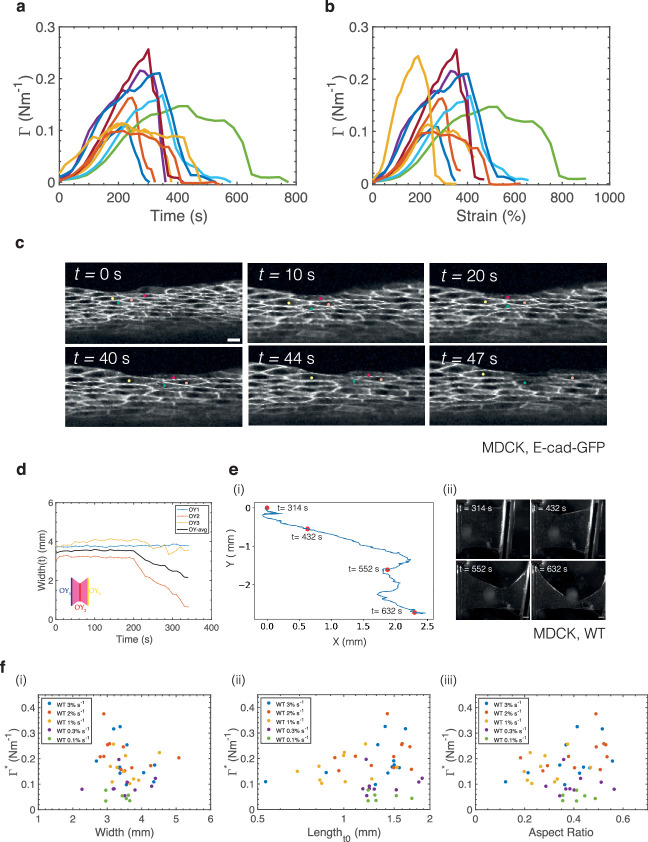
Cells detach from one another during fracture. (**a**) Tension as a function of time and (**b**) Tension as a function of strain for ramp experiments performed at 1% s^−1^ (n = 10 monolayers). Each curve corresponds to a separate monolayer. (**c**) Time series of crack propagation in a monolayer of MDCK cells expressing E-Cadherin-GFP. Several cells are marked by coloured dots in each frame. Time is indicated in the top left corner. Scale bar = 10 *μ*m. (**d**) Temporal evolution of monolayer average width for the monolayer shown in Fig. 1c. The average width of the monolayer OY_avg_ is shown in black and is computed from the width at each of the arms of the device, OY_1_, OY_3_ and the width at the middle of the monolayer OY_2_. These locations are indicated in the schematic diagram in the bottom left of the graph. The colour of the curves correspond to the position where the width is measured. (**e**) Representative propagation of the crack front in an MDCK monolayer subjected to a strain ramp performed at 1% s^−1^. (i) Crack trajectory in the referential of the camera. Each segment corresponds to 1s. The crack starts at the top left and propagates towards the bottom right. Red dots correspond to images in (ii). The timing of each dot is indicated on the graph. (ii) Time series of crack propagation in an MDCK monolayer. (**f**) Rupture tension Γ^*^ as a function of monolayer initial width (i), initial length (ii), and aspect ratio (iii). Each dot corresponds to a separate monolayer. Each colour corresponds to a different strain rate. Source data

**Extended Data Fig. 2:**
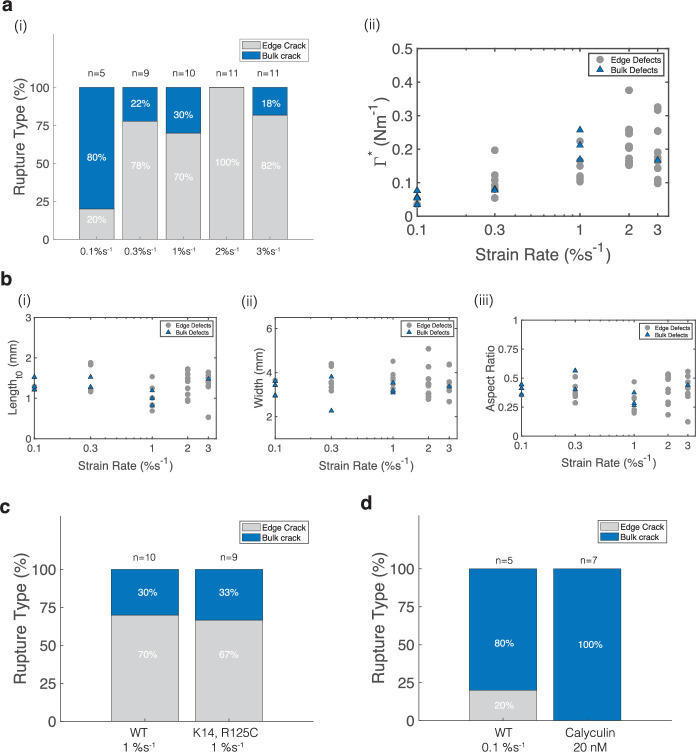
Edge and bulk ruptures in different conditions. Location of initial crack. In all bar charts, The number of monolayers examined is indicated above each bar. (**a**) Location of initial crack as a function of strain rate. (i) Location of first visible crack (bulk in blue vs edge in grey) as a function of strain rate. (ii) Rupture tension as a function of strain rate. Each marker corresponds to a monolayer and indicates the location of the crack: grey dot for edge defects and black triangles for bulk defects. (**b**) Initial length (i), width (ii) and aspect ratio (iii) as a function of strain rate. Each marker corresponds to a monolayer and indicates the location of the crack: grey dot for edge defects and black triangles for bulk defects. (**c**) Location of the first crack for WT and K14,R125C monolayers stretched at 1% s^−1^ strain rate. (**d**) Location of the first crack for WT monolayers stretched at 0.1% s^−1^ and monolayers treated with 20nM calyculin. Source data

After preconditioning (Methods), we subjected monolayers to a ramp in deformation applied at a constant strain rate of 1% s^−1^ (Fig. 1b and Supplementary Video 1) to minimize stress originating from viscoelastic contributions^13^, and we monitored stress as a function of time. Monolayers could withstand a more than threefold increase in length before the first crack appeared and failed for strains of ~300% (Fig. 1c,e). Most cracks first appeared at the free edges, although some also arose in the bulk of the material (Fig. 1c and Extended Data Figs. 1c and 2a). Once nucleated, the crack front propagated following a complex path with alternating periods of rapid propagation and pauses until complete failure (Fig. 1c, Extended Data Fig. 1e and Supplementary Video 1). As the monolayer length and width (in millimetres) are orders of magnitude larger than its thickness (~10 μm), we approximated the tissue to a thin sheet. As the strain in our experiments is far greater than the strain for which monolayer unfurling is observed (<75%) (ref. ^14^), we considered monolayers to have a constant width *w*_0_ and normalized the force *F* to *w*_0_ to generate tension–strain curves (Extended Data Fig. 1d). In response to deformation, tension first rose linearly up to ~50% strain before increasing more rapidly, until reaching a peak tension of Γ* = F*/*w*_0_ (Fig. 1e and Extended Data Fig. 1a,b). Beyond strain ε*, cracks became apparent and tension decreased until complete failure.

In many materials, the peak tension marks the onset of fracture. Therefore, we plotted the strain ε* at which Γ* was reached as a function of strain *ε*_1stdefects_ at which we observed the first monolayer defects in our time-lapse images (Fig. 1c). This revealed a clear correlation (Fig. 1d), suggesting that cracks initiate due to accumulated stress in the tissue. Γ* did not depend on monolayer dimensions, confirming that it represents a material property (Extended Data Fig. 1f). Therefore, in the following, we used Γ* and ε* as parameters to characterize the onset of fracture in epithelial monolayers.

## Cracks occur at cell–cell junctions

At the cell scale, cracks could, in principle, appear either because of cell lysis or detachment at intercellular junctions. Imaging revealed that cells were often very elongated at the free edge of the monolayer where most cracks initiate (Fig. 1g). As strain increased, cell–cell contacts progressively decreased in size perpendicular to the direction of applied stretch before the cells lost contact with one another. The crack front alternated phases of rapid propagation and pauses with no apparent preferred directionality (Extended Data Fig. 1e), but always followed cell–cell junctions, where cells appeared to peel apart. When we examined the localization of the intercellular adhesion protein E-cadherin fused to green fluorescent protein (GFP), its signal disappeared from the cell surface after cell–cell junction rupture (Extended Data Fig. 1c and Supplementary Video 2). In profile views of the monolayers, we observed a progressive decrease in the height of adherens junctions as the strain increased (Fig. 1f). However, what happens to intercellular adhesion proteins at the molecular scale during change in the intercellular junction height remains unclear.

## Monolayers can self-rupture by increasing contractility

In vivo, epithelia can rupture in response to an increase in contractility. In some cases, this compromises the viability of the organism^11^, whereas in others, it forms a part of normal development^15^. To study the response of tissues to active stresses generated by myosins, we treated epithelia with calyculin, a phosphatase inhibitor that increases monolayer tension^16^.

**Fig. 2:**
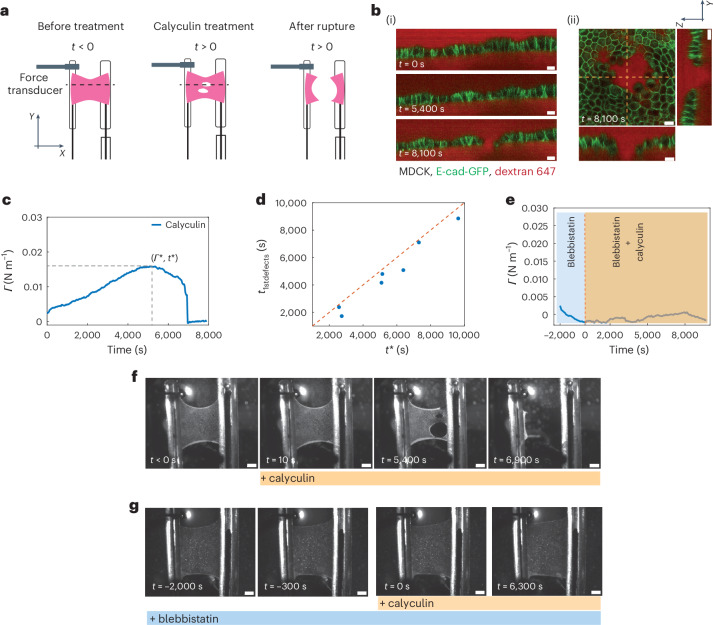
Monolayers can self-rupture by increasing their myosin contractility. **a**, Diagram of the experiment. Monolayers were treated with calyculin, an inhibitor of myosin phosphatase, at *t* = 0. The tension in the monolayer was measured over time and the length of the monolayer was kept constant by the micromanipulator. After some time, defects appeared in the monolayer and measurements were continued until the monolayer failed. The dashed black line indicates a representative position at which the monolayer profile would be imaged. **b**, Representative confocal images of a monolayer profile during calyculin treatment. Intercellular junctions are visualized with E-cadherin-GFP (green) and cells are visualized by dye exclusion of dextran-Alexa 647 (red) added to the medium. Scale bars, 10 μm. (i) Profile view over time. Time is indicated in the bottom-left corner of each image. (ii) *XY* view of a defect in a monolayer and its corresponding *XZ* and *YZ* profile views. The dashed yellow lines indicate the position of the profile views. Time is indicated in the bottom-left corner. **c**, Temporal evolution of tension for the calyculin-treated monolayer shown in **f**. The dashed lines indicate the maximum tension Γ* and its timing t*. **d**, Time at which the first defects *t*_1stdefects_ are observed as a function of the time at which the maximum tension is reached, t*. The dashed red line shows the line of slope 1. **e**, Temporal evolution of tension for a monolayer treated with blebbistatin and calyculin, as shown in **g**. The monolayer was first treated with blebbistatin alone for 2,000 s (blue-shaded region) before calyculin was added (orange-shaded region). **f**, Bright-field microscopy time series showing a representative calyculin-treated monolayer. Calyculin is added to the medium at time 0. Time is indicated in the bottom-left corner. Temporal evolution of tension is shown in **c**. Scale bars, 500 μm. **g**, Bright-field microscopy time series of a representative monolayer treated with blebbistatin and calyculin. Calyculin was added at time 0. Blebbistatin treatment was started at *t* = –2,000 s and was present throughout the experiment. Temporal evolution of tension is shown in **e**. Time is indicated in the bottom-left corner. Scale bars, 500 μm. Source data

**Extended Data Fig. 3:**
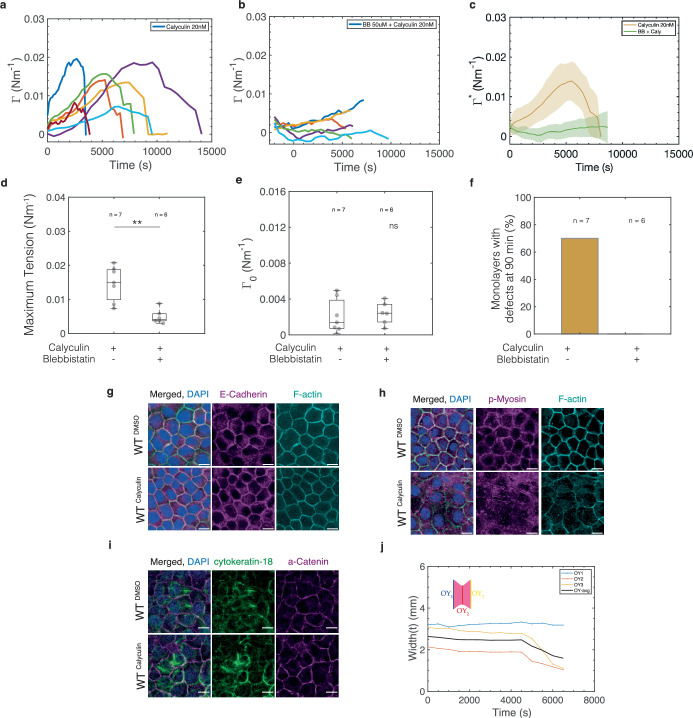
Increase in contractility by calyculin treatment leads to mono-layer rupture. In all box plots, the central mark indicates the median, and the bottom and top edges of the box indicate the 25th and 75th percentiles, respectively. The whiskers extend to 1.5 times the interquartile range from the lower or upper quartile. Data points appear as grey dots. Statistically significant difference: ns non-significant P > 0.05, *P < 0.05, ***P < 0.001, ****P < 0.0001, two-sided Kolmogorov-Smirnov test. (**a**) Temporal evolution of tension for calyculin-treated monolayers (n = 7). Each curve corresponds to a separate monolayer. (**b**) Temporal evolution of tension for monolayers treated with blebbistatin and calyculin (n = 6). Each curve corresponds to a separate monolayer. (**c**) Average temporal evolution of tension (thick lines) for monolayers treated with calyculin only (orange, n = 7) or treated with blebbistatin and calyculin (green, n= 6). Tension in monolayers pre-treated with blebbistatin (green) does not increase as in those treated with calyculin (orange). Shaded areas depict the standard deviation. (**d**) Maximum tension reached in monolayers treated with calyculin alone and calyculin + blebbistatin (p = 0.009). (**e**) Pre-tension in monolayers in each set of experiments. Pre-tension is measured before adding any drug (p = 0.5). (**f**) Percentage of monolayers that show defects during the first 90 min of calyculin treatment for monolayers treated with calyculin alone or calyculin + blebbistatin. (**g-i**) Immunostainings of wild-type MDCK cells treated with DMSO (first row) or calyculin 20nM (second row) for 15-20 min. In all panels, the leftmost column shows an overlay. Scale bars, 10 *μ*m. (**g**) Immunostaining against E-Cadherin (magenta) and F-actin (cyan). (**h**) Immunostaining against phospho-Myosin (p-Myosin, magenta) and F-actin (cyan). (**i**) Immunostaining against cytokeratin-18 (green) and alpha-catenin (magenta). (**j**) Temporal evolution of monolayer average width for the monolayer shown in Fig. 2f. The average width of the monolayer OY_avg_ is shown in black and is computed from the width at each of the arms of the device, OY_1_, OY_3_ and the width at the middle of the monolayer OY_2_. These locations are indicated in the schematic diagram in the top left of the graph. The colour of the curves correspond to the position where the width is measured. Source data

We incubated monolayers with 20 nM calyculin and monitored their tension as their morphology is imaged (Fig. 2a,f and Supplementary Video 3). Monolayers were unperturbed until ~90 min after calyculin addition, when holes appeared in the epithelium (Fig. 2b,f). These holes grew through complex crack propagation, merging together and eventually causing full rupture (Fig. 2f). As in ramp experiments, cracking occurred at intercellular junctions (Fig. 2b). Although E-cadherin was lost from the cell surfaces whose intercellular junctions had ruptured (Fig. 2b), we never observed the loss of E-cadherin preceding crack formation. In contrast to ramp experiments, all the cracks formed in the bulk of the tissue rather than at its edges (Fig. 2f and Extended Data Fig. 2d), perhaps due to the isotropic nature of cortical contractility. From the onset of treatment, tension gradually rose, reaching a peak after ~90 min before decreasing as tissue fracture progresses (Fig. 2c and Extended Data Fig. 3a,c). Similar to the ramp experiments, the time *t*_1stdefects_ at which the first defect was observed was correlated with time t* at which tissue tension was the maximum, further confirming Γ* and t* as good criteria for characterizing rupture onset (Fig. 2d). Intriguingly, Γ* was almost tenfold lower than that for ramps and t* was around tenfold longer.

As calyculin is a broad-spectrum phosphatase inhibitor, we confirmed that the monolayer rupture and the increase in tension are specific to myosin activity. First, using immunostaining, we verified that calyculin increased myosin phosphorylation but did not perturb E-cadherin or cytokeratins for treatment durations over which we typically observed increases in monolayer tension and rupture (Extended Data Fig. 3g–j). Next, we incubated the monolayers with a specific myosin inhibitor, blebbistatin, for 30 min before calyculin addition (Fig. 2g and Supplementary Video 4). This ensured that any effect of calyculin specific to myosin contractility was inhibited. When only blebbistatin was present, tension decreased, as expected^16^ (Fig. 2e, blue-shaded area). After calyculin addition, tension remained low and monolayers did not rupture over durations for which failure occurred when using calyculin alone (Fig. 2e,g, Extended Data Fig. 3b–f and Supplementary Video 5). In summary, increasing myosin contractility in suspended epithelial monolayers is sufficient to generate rupture.

## Rupture strain, tension and timescale with strain rate

**Extended Data Fig. 4:**
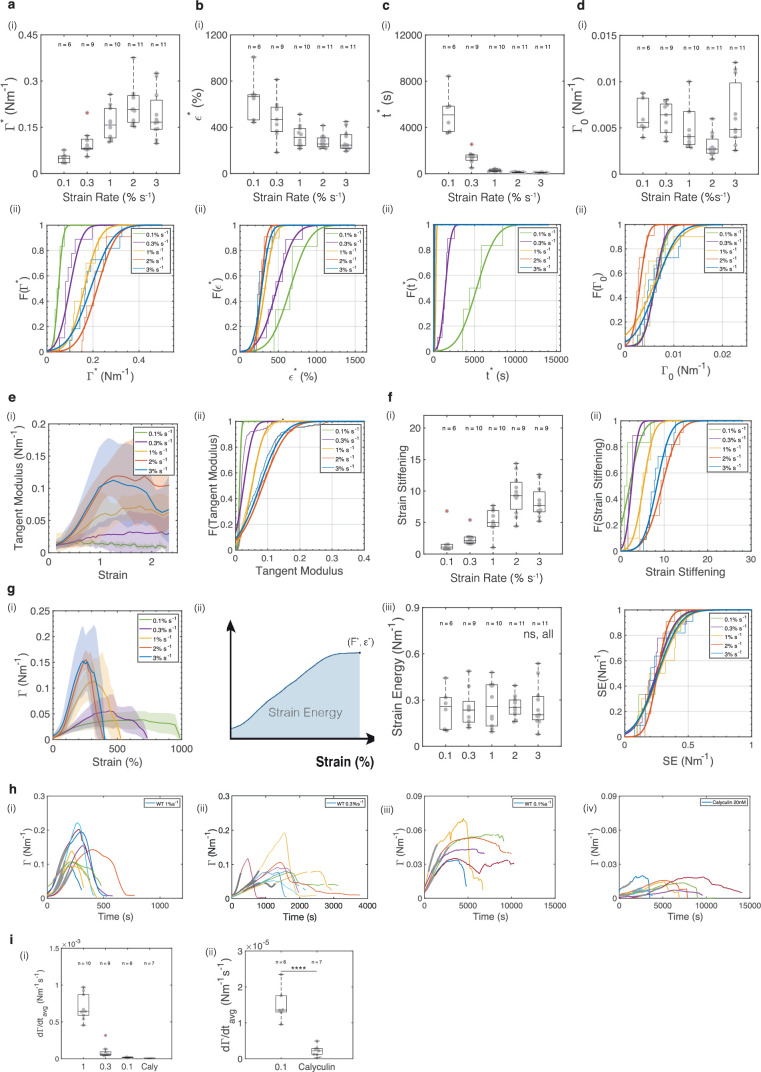
Monolayer rupture characteristics and strain-stiffening scale with strain rate. In all box plots, the central mark indicates the median, and the bottom and top edges of the box indicate the 25th and 75th percentiles, respectively. The whiskers extend to the most extreme data points that are not outliers. Data points appear as grey dots. Outliers are indicated with a red ’+’ symbol. Statistically significant difference: ns non-significant P> 0.05, *P < 0.05, ***P < 0.001, ****P < 0.0001, two-sided Kolmogorov-Smirnov test. Data was acquired from n = 6 monolayers for 0.1% s^−1^, n = 9 for 0.3% s^−1^, n = 10 for 1% s^−1^, n = 11 for 2% s^−1^, and n = 11 for 3% s^−1^. (**a**) Rupture tension, (**b**) rupture strain, (**c**) rupture time and (**d**) pre-tension for different strain rates. (i) Box plots. Statistical analysis for these box plots appear in Supplementary Tables 1 - 4. (ii) Cumulative distribution functions *F*(*x*), for the different rupture parameters *x*. Segmented lines represent the empirical cumulative distribution function whereas the solid curves show a fit. Fitting curves were determined using the cumulative distribution function for a Gaussian distribution evaluated on a given segment of the data. The parameters evaluated correspond to the ones shown in panels **a**-**d** (i). (**e**) Tangent modulus as a function of strain plotted for all strain rates. (i) Graph showing the average value of the gradient (thick lines) and its standard deviation (shaded area) for all strain rates. (ii) Cumulative distribution functions of the tension gradient. (**f**) Fold change in tangent modulus between 15% and 120% strain as a function of strain rate. (i) Box plots of the strain-stiffening between 15% and 120% strain for all strain rates. Statistical analysis appears in Supplementary Table 1. (ii) cumulative distribution functions. (**g**) Strain energy. (i) Evolution of tension as a function of strain for different strain rates. Thick lines indicate the average and shaded areas show the standard deviation. (ii) The strain energy is calculated as the integral of the tension-strain curve up until rupture (Γ^*^, ϵ^*^). (iii) Left: box plots of strain energy as a function of strain rate. Right: cumulative distribution functions. (**h**) Temporal evolution of tension for wild-type monolayers stretched at (i) 1% s^−1^, (ii) 0.3% s^−1^ (iii) 0.1% s^−1^, and (iv) treated with 20nM calyculin. Each line represents an individual monolayer. (**i**) Average rate of increase in tension for different strain rates and calyculin. The rate of increase in tension was calculated from the region indicated in grey in each tension-time curve in h. (ii) Same as in (i) but with a scale optimised for 0.1% s^−1^ strain rate and calyculin. Source data

During normal physiological function, epithelia experience strain rates of up to 100% s^−1^ (ref. ^17^). To explore strain rate dependency while minimizing viscous contributions, we examined rupture in monolayers subjected to ramps in deformation for strain rates of 0.1–3% s^−1^ (Extended Data Fig. 4g).

**Fig. 3:**
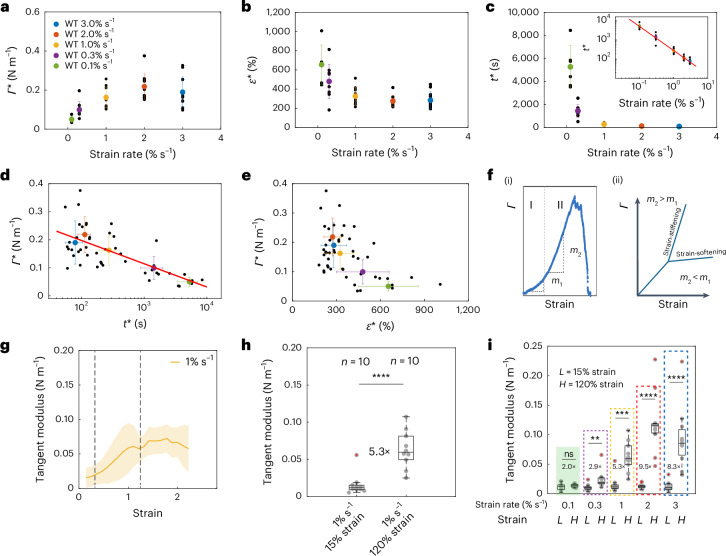
Rupture characteristics depend on strain rate. In the box plots, the central mark indicates the median, and the bottom and top of the box indicate the 25th and 75th percentiles, respectively. The whiskers extend to the most extreme data points that are not outliers. Data points are indicated by grey dots and outliers, as red + symbols. Statistics: two-sided Kolmogorov–Smirnov test; ns, non-significant; **P < 0.01, ***P < 0.001, ****P < 0.0001. Data from *n* = 6 monolayers for 0.1% s^−1^, *n* = 9 for 0.3% s^−1^, *n* = 10 for 1% s^−1^, *n* = 11 for 2% s^−1^ and *n* = 11 for 3% s^−1^. **a**–**c**, Rupture tension (**a**), rupture strain (**b**) and rupture time (**c**) as functions of the strain rate. The inset in **c** shows data on a log–log scale. **d**, Rupture tension as a function of the rupture time plotted on a semi-log scale. The red line is a linear fit to the experimental data. **e**, Rupture tension as a function of rupture strain. In **a**–**e**, the black dots represent individual monolayers, coloured dots represent the average value and whiskers indicate the standard deviations. **f**, Monolayers display strain-stiffening above 50% strain. (i) Representative tension–strain curve for a monolayer displaying strain-stiffening. The dashed line delimits the approximate change in regime. Slope *m*_2_ in region II is larger than slope *m*_1_ in region I. (ii) Example tension–strain curve for a material that strain-stiffens (slope increases) or strain-softens (slope decreases). **g**, Tangent modulus as a function of strain for ramps at 1% s^−1^. The thick line represents the average value and the shaded area, the standard deviation (*n* = 10). The dashed lines show the strains at which the tangent moduli were measured in **h**. **h**, Tangent modulus at low strain (*ε* = 15%) and high strain (*ε* = 120%) for experiments pooled in **g**. Fold change is indicated between the two strain magnitudes (*n* = 10). *P* = 0.00017. **i**, Tangent modulus at low strain (*L* ≃ 15%) and at high strain (*H* ≃ 120%) as a function of strain rate. Fold change is indicated between the two strain magnitudes (*n* = 10). *P* = 0.81, *P* = 0.003, *P* = 0.00017, *P* = 7 × 10^−6^ and *P* = 6 × 10^−5^ for ramps performed at 0.1, 0.3, 1, 2 and 3% s^−1^, respectively. Source data

For each monolayer, we characterized Γ* as well as ε* and t* and plotted our data as a function of the strain rate. Γ* increased with strain rate, from 0.04 N m^−1^ at 0.1% s^−1^ until seemingly reaching a plateau of ~0.20 N m^−1^ for strain rates above 2% s^−1^ (Fig. 3a and Extended Data Fig. 4a–d). In contrast, both ε* and t* decreased with increasing strain rate. Rupture strain decreased from ~700% at 0.1% s^−1^ until reaching a plateau of ~250% for larger strain rates (Fig. 3b and Extended Data Fig. 4b). Rupture time t* spanned nearly two orders of magnitude decreasing from 5 × 10^3^ s to 10^2^ s (Fig. 3c and Extended Data Fig. 4c). As no cell rearrangements and only few divisions take place over hour-long durations in our suspended monolayers^18^, these large deformations are probably accommodated through stretching and remodelling of the cytoskeleton and adhesive complexes. Interestingly, for the lowest strain rate (0.1% s^−1^), Γ* and t* were comparable to those observed in response to calyculin treatment, suggesting that rupture due to active stresses generated by myosin and passive stresses due to deformation of the cytoskeleton may arise from the same biophysical processes.

**Extended Data Fig. 5:**
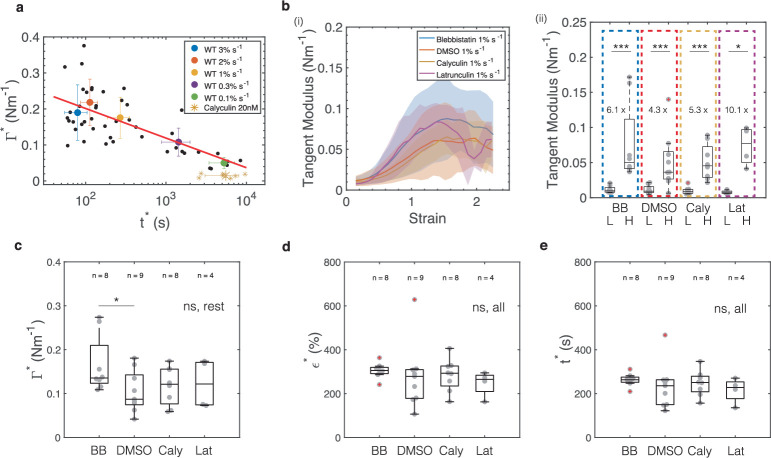
Strain stiffening does not depend on the actomyosin cytoskeleton. In all box plots, the central mark indicates the median, and the bottom and top edges of the box indicate the 25th and 75th percentiles, respectively. The whiskers extend to the most extreme data points that are not outliers. Data points appear as grey dots. Outliers are indicated with a red ’+’ symbol. Statistically significant difference: ns non-significant P > 0.05, *P < 0.05, ***P < 0.001, ****P< 0.0001, two-sided Kolmogorov-Smirnov test. (**a**) Semi-log graph of the rupture tension as a function of the rupture time for all strain rates and for calyculin-treated monolayers. Black dots represent individual monolayers, except for calyculin treated monolayers that are represented by yellow asterisks. Coloured dots represent the average value and whiskers represent the standard deviations, except for calyculin treated monolayers that are represented by a cross. Note that calyculin treated monolayers cluster very close to the trend line expected for experiments in which strain rate is varied. (**b**) Tangent modulus for monolayers in which actomyosin has been perturbed. (i) Average tangent modulus (thick lines) and standard deviation (shaded area) as a function of strain for ramp experiments performed at 0.3% s^−1^ for monolayers pre-incubated with blebbistatin 50 *μ*M (BB, blue, n = 8), DMSO (red, n = 9), calyculin 20 nM (Caly, yellow, n = 8) and latrunculin 1 *μ*M (Lat, purple, n = 4). (ii) Boxplots comparing the tension gradient at low strain (L ~ 15% strain) and high strain (H ~ 120 % strain) for monolayers treated with drugs perturbing the actomyosin cytoskeleton. (c-e) Boxplots showing the (**c**) rupture tension (p = 0.02 between BB and DMSO monolayers, p = 0.5 between BB and calyculin-treated monolayers, p = 0.4 between BB and latrunculin-treated monolayers, p = 0.7 between DMSO and calyculin-treated monolayers, p = 0.7 between DMSO and latrunculin-treated monolayers, and p = 0.7 between calyculin and latrunculin-treated monolayers), (**d**) rupture strain (p = 0.3 between BB and DMSO monolayers, p = 0.5 between BB and calyculin-treated monolayers, p = 0.05 between BB and latrunculin-treated monolayers, p = 0.8 between DMSO and calyculin-treated monolayers, p = 0.8 between DMSO and latrunculin-treated monolayers, and p = 0.4 between calyculin and latrunculin-treated monolayers), and (**e**) rupture time for monolayers pre-incubated with different treatments perturbing the actomyosin cytoskeleton (p = 0.3 between BB and DMSO monolayers, p = 0.5 between BB and calyculin-treated monolayers, p = 0.2 between BB and latrunculin-treated monolayers, p = 0.6 between DMSO and calyculin-treated monolayers, p = 0.99 between DMSO and latrunculin-treated monolayers, and p = 0.7 between calyculin and latrunculin-treated monolayers). Source data

To gain an insight into the failure mechanism, we plotted rupture tension as a function of rupture time and rupture strain. This revealed that Γ* scaled as ~log(1/t*) (Fig. 3d), reminiscent of the failure dynamics of groups of bonds subjected to force^19,20^. Experiments increasing contractility clustered close to those from 0.1% s^−1^ ramps (Extended Data Fig. 5a). However, the rate of increase in tension dΓ*/d*t* was several fold larger in ramp experiments (Extended Data Fig. 4h,i), suggesting fundamental differences between these experimental conditions. In deformation experiments, Γ* decreased linearly with ε* (Fig. 3e) and the strain energy at rupture onset remained approximately constant across the strain rates (Extended Data Fig. 4g). The decrease in Γ* with ε* is surprising because our previous work suggested that monolayers behave as elastic solids for strain rates lower than 1% s^−1^ (ref. ^13^). In elastic solids, tension increases with strain, contrary to what is observed for rupture tension in our experiments (Fig. 3e). Such discrepancy may be due to the very large deformations used in the current study.

## Monolayers display a strain-rate-dependent strain-stiffening

One potential origin for this counter-intuitive behaviour could involve a change in the mechanical response of the monolayer with strain rate. In monolayers stretched at 1% s^−1^, the slope of the tension–strain curve visibly increased for strains greater than 50% (Fig. 1e). This feature, known as strain-stiffening (Fig. 3f), also occurs in biopolymer networks, allowing them to limit deformation^21^. Therefore, we examined how strain-stiffening changed with the strain rate. When we computed the gradient of the tension–strain curve (that is, the tangent modulus) for monolayers subjected to ramps at 1% s^−1^, we observed three distinct regimes: until ~30% strain, the tangent modulus was constant; then, between ~50% and 100%, it increased monotonically with strain; and finally, from around 100% strain, it reached a plateau (Fig. 3g), with a value almost fivefold larger than that in the first regime (Fig. 3h). Intriguingly, strain-stiffening was dependent on the strain rate (Fig. 3i). At the lowest strain rate (0.1% s^−1^), the tangent modulus did not change at high strain, but from 0.3% s^−1^, it increased with the strain rate, saturating for rates above 2% s^−1^—a behaviour known as shear-stiffening (Extended Data Fig. 4e,f)^22,23^.

## Keratin networks control tissue strength and strain-stiffening

After showing that strain-stiffening did not depend on mechanotransductory processes or actomyosin (Supplementary Results and Extended Data Fig. 5), we focused our attention on keratin intermediate filaments. These form entangled networks around the nucleus with wavy filament bundles radiating out towards the cell periphery where they connect to neighbouring cells via specialized complexes known as desmosomes. These comprise desmosomal cadherins whose extracellular domains bind to counterparts on adjacent cells as their cytoplasmic domains connect to keratin filaments via anchor proteins, such as desmoplakin^24^. Keratins can stretch multiple times their original length before rupture, can bear high tensile loads and form networks that strain-stiffen in vitro^25,26^, making them good candidates to maintain tissue integrity and control monolayer rheology at high strain. Previous work has shown that as epithelia are stretched, desmosomes become progressively load bearing^27^. Furthermore, mutations in keratins and desmosomal proteins give rise to skin-blistering disorders that display symptoms of mechanical fragility^3^. Thus, the supracellular network formed by keratins and desmosomes may contribute to tissue strength.

**Fig. 4:**
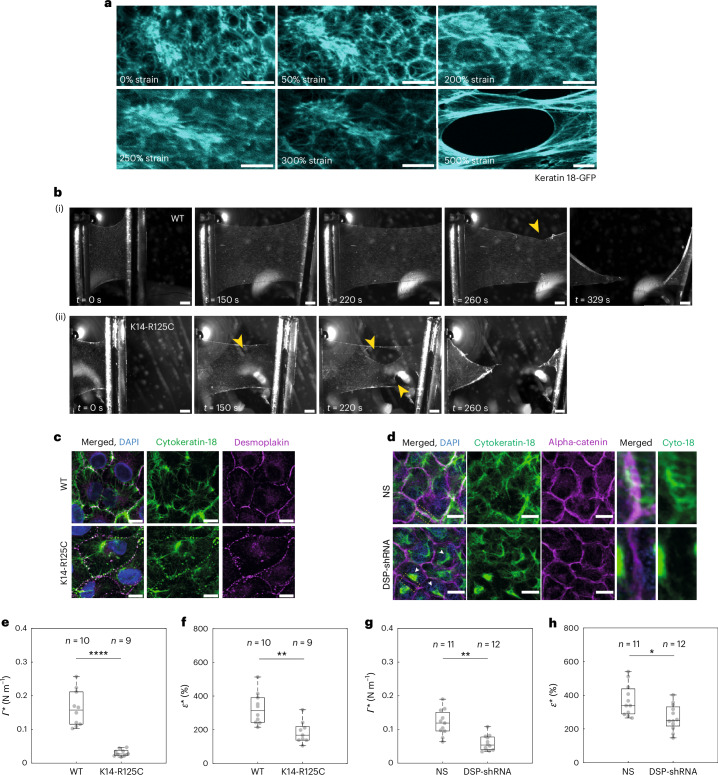
Perturbation of the keratin intermediate filament network fragilizes monolayers. **a**, Image series of keratin 18-GFP localization in cells of suspended monolayers subjected to increasing deformation. The strain is indicated in the bottom-left corner. Scale bars, 10 μm. **b**, Bright-field microscopy time series of representative WT (i) and K14-R125C (ii) monolayers during a ramp experiment performed at 1% s^−1^. The arrowheads indicate the start and growth of cracks. Time is indicated in the bottom-left corner. Scale bars, 500 μm. **c**, Immunostaining of cytokeratin-18 (green) and desmoplakin (magenta) in WT (top row) and K14-R125C monolayers (bottom row). Scale bars, 10 μm. **d**, Immunostaining of cytokeratin-18 (green) and desmoplakin-alpha-catenin (magenta) in NS (top row) and desmoplakin-shRNA monolayers (DSP-shRNA, bottom row). Scale bars, 10 μm. The 4th and 5th columns show the zoomed-in views of an intercellular junction. **e**–**h**, In all the box plots, the central mark indicates the median, and the bottom and top edges of the box indicate the 25th and 75th percentiles, respectively. The whiskers extend to the most extreme data points that are not outliers. Data points appear as grey dots. Statistically significant difference: *P* > 0.05, *P < 0.05, **P < 0.01, ****P < 0.0001, two-sided Kolmogorov–Smirnov test. **e**,**f**, Box plots comparing the rupture tension (*P* = 3 × 10^−5^) (**e**) and rupture strain (*P* = 0.003) (**f**) between WT and K14-R125C monolayers. **g**,**h**, Box plots comparing the rupture tension (*P* = 0.0017) (**g**) and rupture strain (*P* = 0.02) (**h**) between NS and DSP-shRNA monolayers. Source data

We first imaged the response of the keratin network to deformation in monolayers stably expressing K18-GFP. At low strain, keratin bundles extending towards the cell periphery displayed wavy morphologies, indicating they were not under tension (Fig. 4a). As the strain increased, the network became progressively stretched in the direction of deformation, suggesting that keratin bundles become loaded as previously reported^28,29^. Even above 300% strain, the network retained its structural integrity.

Next, we perturbed keratins and desmosomes in turn and assessed the impact on tissue mechanics. To study the contribution of keratins, we overexpressed a dominant mutation of keratin 14, namely, K14-R125C, identified in some epidermolysis bullosa patients^3,30,31^. This mutation leads to keratin aggregation, a highly disrupted keratin network and very few connections of keratins to desmosomes compared with control monolayers, consistent with previous reports^30^ (Fig. 4c). To determine if the connection between cellular keratin networks is important for tissue mechanics, we also perturbed desmosomes by stably depleting desmoplakin (DSP-shRNA). In DSP-shRNA cells, the keratin network failed to attach to intercellular junctions (Fig. 4d, zoomed-in region) and often appeared collapsed around the nucleus (Fig. 4d, arrowheads).

**Fig. 5:**
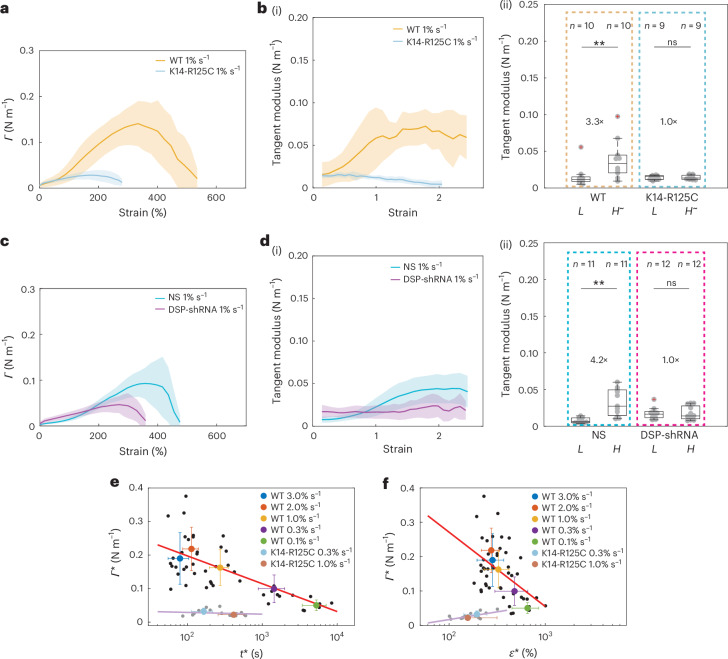
Keratin networks control tissue strain-stiffening and strength. In the box plots, the central mark indicates the median, and the bottom and top of the box indicate the 25th and 75th percentiles, respectively. The whiskers extend to the most extreme data points that are not outliers. The data points are indicated by grey dots and the outliers, by red ‘+’ symbols. Statistics: two-sided Kolmogorov–Smirnov test, **P < 0.01. Data from *n* = 10 WT, *n* = 9 K14-R125C, *n* = 11 NS and *n* = 12 DSP-shRNA monolayers. **a**–**d**, The solid lines represent the average value and the shaded areas, the standard deviation. **a**, Tension as a function of strain for ramps at 1% s^−1^ for WT (orange) and K14-R125C (blue) monolayers. **b**, (i) Tangent modulus as a function of strain for ramps at 1% s^−1^ for WT (orange) and K14-R125C (blue) monolayers. (ii) Box plots of the tangent modulus at low strain (*L* ≃ 15%) and at high strain (*H* ≃ 70%) in WT and K14-R125C monolayers. Fold change is indicated between the box plots. *P* = 0.007 and *P* = 0.96 for WT and K14-125 monolayers, respectively. **c**, Tension as a function of strain for ramps at 1% s^−1^ for NS (blue) and DSP-shRNA (magenta) monolayers. **d**, (i) Tangent modulus as a function of strain for monolayers subjected to ramps at 1% s^−1^ for NS (blue) and DSP-shRNA (magenta). (ii) Box plots of the tangent modulus at low strain (*L* ≃ 15%) and at high strain (*H*^~^ ≃ 120%) in NS-shRNA and DSP-shRNA monolayers. Fold change is indicated between the box plots. *P* = 0.003 and *P* = 0.8 for NS and DSP-shRNA monolayers, respectively. **e**,**f**, Data points acquired for different strain rates. The black dots show individual WT and the grey dots, individual K14-R125C monolayers. The coloured dots show the population average for a given strain rate. The whiskers indicate the standard deviation. The red and pink lines are linear regressions to the data. **e**, Rupture tension as a function of rupture time. **f**, Rupture tension as a function of rupture strain. Source data

**Extended Data Fig. 6:**
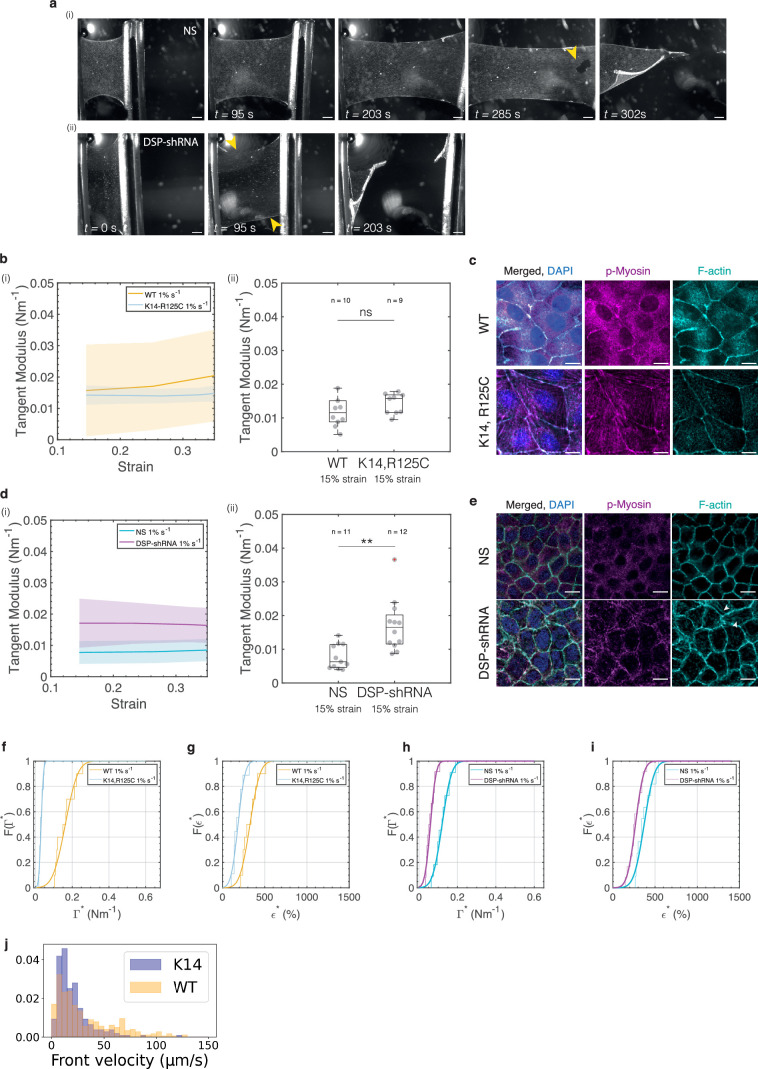
Strain stiffening arises from the intermediate filament cytoskeleton. In all box plots, the central mark indicates the medi-an, and the bottom and top edges of the box indicate the 25th and 75th percentiles, respectively. The whiskers extend to the most extreme data points that are not outliers. Data points appear as grey dots. Outliers are indicated with a red ’+’ symbol. Statistically significant difference: ns non-significant P > 0.05, *P < 0.05, ***P < 0.001, ****P < 0.0001, two-sided Kolmogorov-Smirnov test. (**a**) Bright-field time series of representative (i) non-silencing shRNA (NS) and (ii) desmoplakin-shRNA (DSP-shRNA) monolayers during a ramp experiment performed at 1% s^−1^. Arrowheads indicate the onset of rupture. (**b**) Tangent modulus at low strain for WT (yellow, n = 10) and K14,R125C (blue, n = 9) monolayers subjected to a ramp at 1% s^−1^. (i) Tangent modulus as a function of strain. Solid lines indicate the average value of the tension gradient over strain and shaded areas show the standard deviation. (ii) Box plots of the tangent modulus at 15% strain in WT and K14,R125C monolayers (p = 0.4). The number of monolayers examined is indicated above the box plots. (**c**) Immunostaining against p-myosin (magenta) and F-actin (cyan) for WT (top row) and K14,R125C (bottom row) monolayers. Scale bar= 10*μ*m. (**d**) Tangent modulus at low strain in NS control (cyan, n= 11) and DSP-shRNA (magenta, n = 12) monolayers subjected to a ramp in deformation at 1% s^−1^. (i) Tangent modulus as a function of strain. Solid lines represent the average value and shaded areas show the standard deviation of the distribution. (ii) Box plots of the tangent modulus at 15% strain for NS control and DSP-shRNA monolayers (p = 0.0017). (**e**) Immunostaining against p-myosin (magenta) and F-actin (cyan) for NS-shRNA (top row) and DSP-shRNA (bottom row) monolayers. Scale bar= 10 *μ*m. (**f-i**) Cumulative distribution functions computed from the box plots in Fig. 4e-h. (**f-g**) Cumulative distribution function for the rupture tension (**f**) and rupture strain (**g**) for WT and K14,R125C monolayers. (**h-i**) Cumulative distribution functions of the rupture tension (**h**) and rupture strain (**i**) for NS-shRNA control and DSP-shRNA monolayers. (**j**) Distribution of crack front velocities for wild-type monolayers (WT, orange) and K14,R125C monolayers (K14, purple). Source data

**Extended Data Fig. 7:**
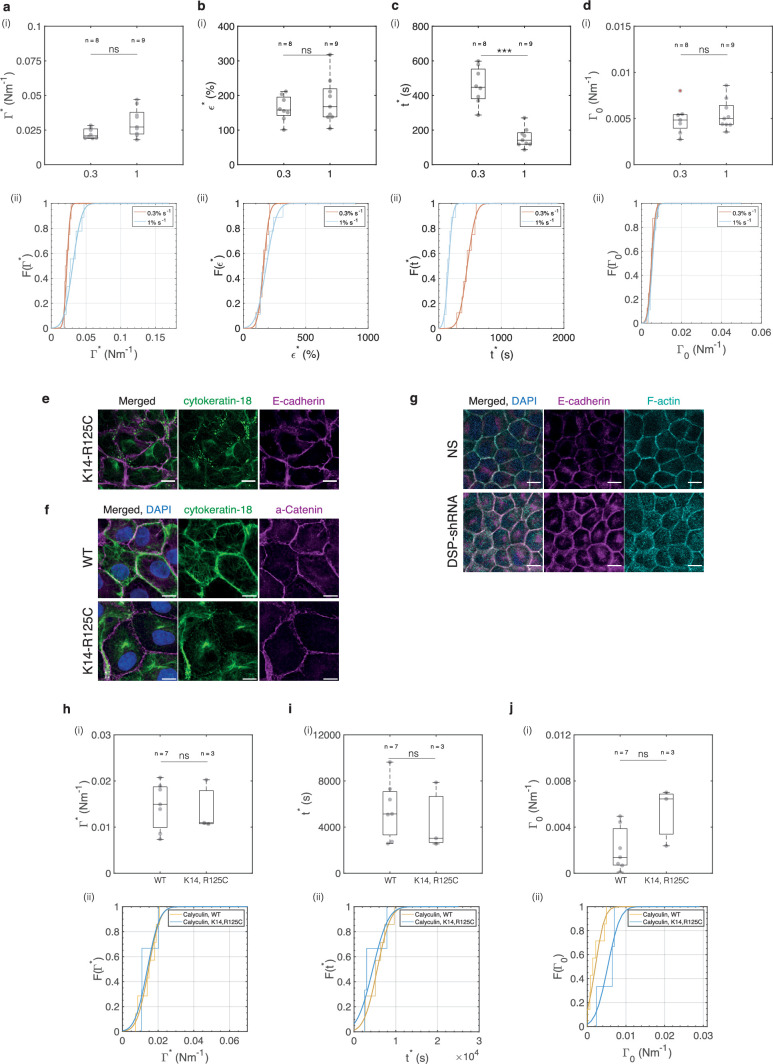
Strain rate dependency of rupture characteristics depends on intermediate filaments. In all box plots, the central mark indicates the median, and the bottom and top edges of the box indicate the 25th and 75th percentiles, respectively. The whiskers extend to the most extreme data points that are not outliers. Data points appear as grey dots. Outliers are indicated with a red ’+’ symbol. Statistically significant difference, ns non-significant P > 0.05, *P < 0.05, ***P < 0.001, ****P < 0.0001, two-sided Kolmogorov-Smirnov test. (**a**) Rupture tension (p = 0.3), (**b**) rupture strain (p = 0.97), (**c**) rupture time (p = 0.0001), and (**d**) pre-tension (p = 0.9) in K14,R125C monolayers subjected to a ramp in deformation at 0.3% s^−1^ and 1%s^−1^. (i) Box plots. The number of monolayers is indicated above each box. (ii) Cumulative distribution functions. In these plots, data from monolayers subjected to ramps with a strain rate of 0.3% s^−1^ are shown in orange and those at 1% s^−1^ are shown in blue. (**e-g**) Left panels are merges of middle and right panels. In some, DAPI nuclear staining is also overlaid. Scale bar = 10 *μ*m. (**e**) Immunostaining of cytokeratin-18 (green) and E-cadherin (magenta) in K14,R125C monolayers. (**f**) Immunostaining of cytokeratin-18 (green) and alpha-Catenin (magenta) in wild-type (WT, top row) and K14,R125C (bottom row) monolayers. (**g**) Immunostaining of E-cadherin (magenta) and F-actin (cyan) in NS control (top row) and DSP-shRNA monolayers (bottom row). (**h**) Rupture tension (p = 0.8), (**i**) rupture time (p = 0.8), and (**j**) pre-tension (p = 0.13) in WT and K14,R125C monolayers treated with 20nM calyculin. (i) Box plots. The number of monolayers is indicated above each box. (ii) Cumulative distribution functions computed from box plots in (i). In these plots, data from WT monolayers treated with calyculin are shown in orange and those from K14,R125C monolayers treated with calyculin are shown in blue. Source data

**Extended Data Fig. 8:**
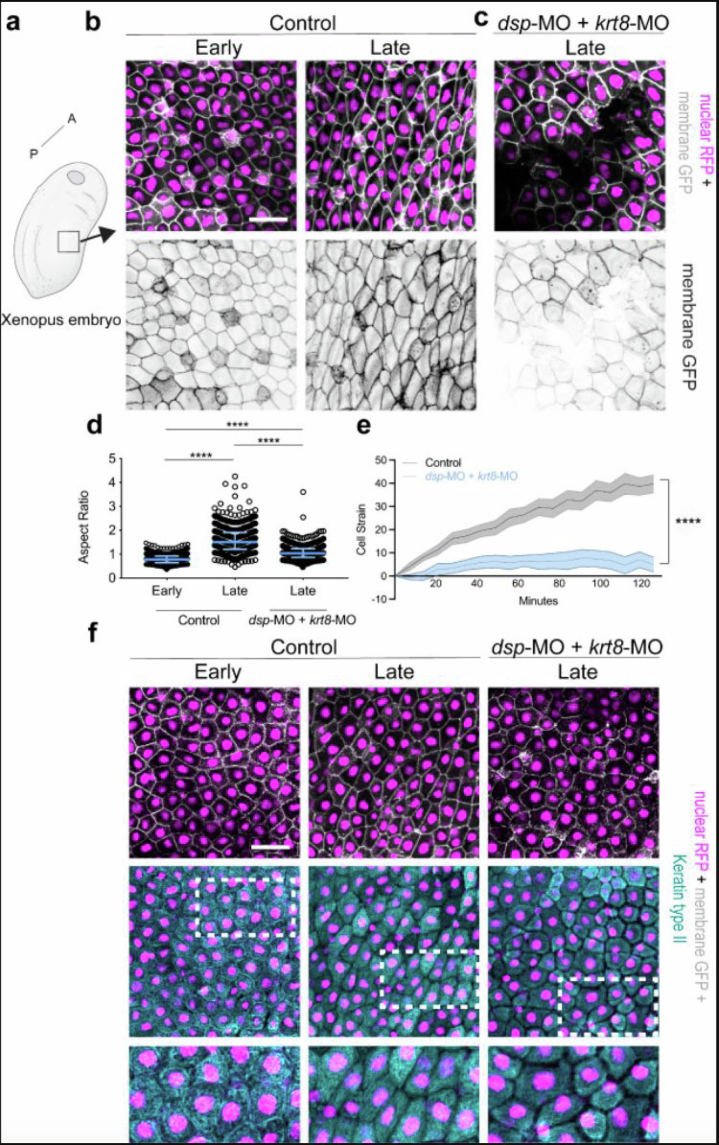
Perturbation of the keratin intermediate filament network in *Xenopus laevis* embryos leads to tissue rupture during development. (**a**) Schematic representation of a *Xenopus laevis* embryo. A, anterior; P, posterior. The black box in the lateral epidermis indicates the region of interest imaged in b, c, and f. (**b**) Representative confocal projections of the epidermal cells at early (stage 16) and late (stage 21-22) stages of body axis elongation. (**c**) Representative confocal projections showing the impact of desmoplakin and keratin 8 knockdown in the lateral epidermis at late stages of body axis elongation. (**b**, **c**) Top row: composite image showing the nucleus in magenta and the membrane in white. Bottom row: membrane GFP. (**d**) Aspect ratio in early and late control embryos and in late embryos depleted in desmoplakin and keratin 8. The bar represents the median and the whiskers indicate the interquartile ranges; individual comparisons two-sided Mann Whitney test. p=0.0001 Early vs Late control, p=0.0001 Late control vs Late dsp-MO+krt8-MO. (**e**) Cell strain as a function of time for control (black) and knockdown (blue) embryos. Solid lines represent the average value and shaded areas show the standard deviation of the distribution. Unpaired two-tailed t-test p=0.0001. (**f**) Confocal projections of embryos immunostained against keratin type II. Left: control embryos at early and late stages. Right: Embryos depleted in desmoplakin and keratin 8 at late stages of body axis elongation. Top row: overlay of membrane-GFP (white) and nucleic acids (magenta). Middle row: Overlay of cytokeratin (cyan, immunostaining) and nucleic acids (magenta). Bottom row: Zoom of the region in the dashed white box in the middle row. Scale bars in b, c, and f: 40 *μ*m. b,c,f are representative examples of at least three independent experiments, C.I. 95%. Source data

We then examined their impact on the tissue mechanical response by performing ramp experiments at 1% s^−1^ (Fig. 4b, Extended Data Fig. 6a and Supplementary Videos 1 and 10–12). Both perturbations led to earlier rupture than in controls (Fig. 4b and Extended Data Fig. 6a (arrowheads)), but neither the crack propagation velocity (Extended Data Fig. 6j) nor the location of crack nucleation were affected (Extended Data Fig. 2c). Quantitatively, rupture strain ε* and rupture tension Γ* were significantly lower in the perturbed tissues (Fig. 4e–h and Extended Data Fig. 6f–i). These data confirm that keratin networks connected across cells are necessary for monolayers to resist large deformations. We next investigated the impact on strain-stiffening by computing the tangent modulus (Fig. 5a–d). With either perturbation, there was no increase in the tangent modulus with strain in contrast to controls (Fig. 5b,d). Furthermore, the strain rate dependency of rupture characteristics was lost with keratin depletion (Supplementary Results and Extended Data Fig. 7). We also verified that changes in actomyosin did not perturb the keratin networks or rupture characteristics (Extended Data Fig. 6b–e, Supplementary Results and Supplementary Fig. 4). Furthermore, we showed that a supracellular network of keratins linked by desmosomes is critical during body-axis elongation in *Xenopus laevis* (Supplementary Results and Extended Data Fig. 8).

Overall, we concluded that keratin networks are primarily responsible for load bearing at high strain as well as for strain-stiffening.

### Rheology and intercellular adhesion control tissue strength

The onset of rupture is associated with the separation of cell junctions. At the molecular level, this implies unstable dynamics with bonds dissociating more frequently than associating. Simple models of interfaces linked by dynamic bonds have already demonstrated that a molecular slip bond behaviour leads to a finite time of separation that decreases with the applied mechanical force^19,20^. Such a trade-off is consistent with our observation that monolayers reach higher rupture tension Γ* and shorter rupture times t* at large strain rates (Fig. 3a,c). However, we also observed that ε* decreased with strain rate (Fig. 3b): the higher the rupture tension, the lower the strain at rupture (Fig. 3e). Remarkably, this qualitative trend disappears when the keratin network is disrupted (Fig. 5e). Although the separation of a junction is primarily controlled by the tension applied to it, in our experiments, this tension is not controlled directly; rather, it arises indirectly from the material’s rheology because of the applied deformation ramp. Thus, the onset of rupture involves the interplay between the tissue-scale rheology and the molecular-scale dynamics of bonds.

**Fig. 6:**
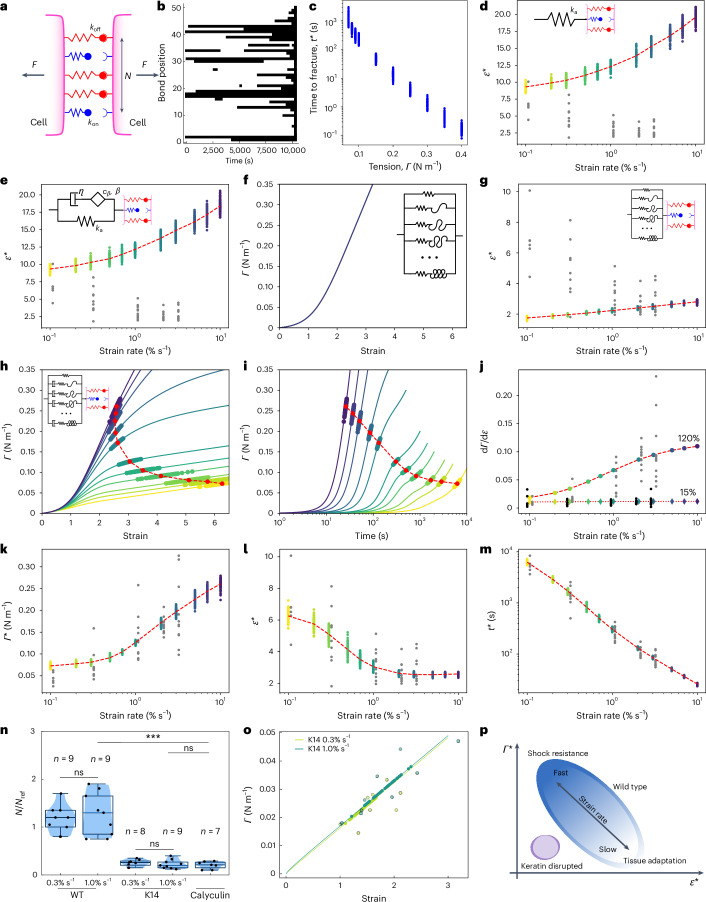
Multiscale modelling of rupture onset. In **d**, **e** and **g**–**m**, the coloured dots represent the simulation runs (100 runs per strain rate). Yellow denotes low strain rates and dark blue denotes high strain rates. The grey dots represent experimental data points and the red dots indicate the mean for a series of simulations. The dashed red lines link the mean values to show the trend. In **d**–**h**, the insets show the rheological model. **a**, Cell surfaces are subjected to force *F* and linked by *N* independent linkers with slip bond dynamics. Each linker can associate with a counterpart with rate *k*_on_ and dissociate with rate *k*_off_ that depends on the force *f* applied to it: *k*_off_ = *k*_off,0_e^*f*/*f*0^, where *f*0 is a model parameter. *F* is assumed to be equally shared between closed bonds. **b**, Typical temporal evolution of the states of 50 linkers subjected to constant force. Black indicates an unbound state. The simulation ends when all the linkers are unbound, defining *t* = t*. **c**, t* as a function of tension when subjected to a constant tension. **d**,**e**, ε* as a function of strain rate for a linear elastic material (**d**) and a linear viscoelastic material (**e**). **f**, Tension–strain relationship for a strain-stiffening material. **g**, ε* as a function of strain rate for a strain-stiffening material. **h**,**i**, Tension–strain (**h**) and tension–time (**i**) characteristics (solid lines) for a shear-stiffening material. Each curve represents a different strain rate. **j**, Tangent modulus at 15% and 120% strain as a function of strain rate for a shear-stiffening material. The rheological model is presented as the inset in **h**. The grey dots show the experimental data at 120% and black dots at 15% strain. **k**–**m**, Graphs showing the tension (**k**), strain (**l**) and time (**m**) at rupture as a function of the strain rate for shear-stiffening rheology. **n**, Normalized linker numbers required to fit the WT, calyculin-treated WT and K14-R125C monolayers. In the box plots, the central mark indicates the median. The bottom and top of the box indicate the 25th and 75th percentiles, respectively. The whiskers extend to the most extreme data points that are not outliers. Data points are indicated by grey dots. Statistics: two-sided Wilcoxon rank-sum test, ***P < 0.001. *P* = 0.75 between WT at 0.3% s^−1^ and 1% s^−1^, *P* = 0.001 between K14 at 0.3% s^−1^ and 1% s^−1^, *P* = 0.44 between K14 at 1% s^−1^ and calyculin, and *P* = 0.87 between WT at 1% s^−1^ and calyculin. **o**, Tension as a function of strain for the linear viscoelastic model presented in **e**, adjusted in stiffness to match the experimental stress values for the K14-R125C monolayers. Stochastic bond model parameters from **n** are used to predict Γ* for ramps at 0.3% s^−1^ and 1% s^−1^ (filled green circles). The open circles represent the experimental Γ* and ε* values for K14-R125C monolayers. **p**, Summary diagram showing the range of behaviours observed. Source data

To dissect the respective contributions of rheology and adhesion, we interfaced a stochastic slip bond dynamics model that predicts fracture in response to a time-dependent force^19,20^, with a rheological model that outputs the force in the material in response to a time-dependent deformation (Methods). The stochastic bond model consists of two surfaces representing an intercellular junction connected by a population of *N* independent linkers that can exist in two states: bound or unbound (Fig. 6a). They bind at a fixed rate *k*_on_, but unbind at a rate *k*_off_ that increases exponentially with mechanical load with scale *f*_0_. In our implementation, all the bound links bear an equal share of the applied load. Rupture occurs when all the links detach (Fig. 6b). After validating this model (Fig. 6c), we examined its response when combined with a rheological model representing the tissue. We first implemented commonly used cell rheologies (elastic, viscoelastic and power law), but all of them led to a monotonically increasing relationship between ε* and strain rate, contrary to our observations (Supplementary Results and Fig. 6d,e).

Our experiments show that the material stiffens for strains greater than 50%, but also that stiffening is larger for large strain rates. Strain-stiffening is a classical feature of random fibre networks^32^. Conceptually, as the strain increases, more and more fibres align along the direction of deformation and become taut. Thus, more fibres carry the mechanical load and the stiffness increases. In our case, keratin bundles are responsible for both strain-stiffening and shear-stiffening (Fig. 5). As keratin filaments do not intrinsically strain-stiffen^33^, we hypothesize that the progressive recruitment of keratin bundles to bear load underlies the strain-stiffening. To mimic this, we introduce a nonlinear elastic model where springs are progressively recruited as the deformation increases (Fig. 6f (inset) and Methods), leading to strain-stiffening (Fig. 6f). Although the model can be calibrated to provide good agreement with the experimental stress at high strain rates (Fig. 6g), it still fails to capture the qualitative relationship between ε* and strain rate (Fig. 6g).

The strain-rate-dependent strain-stiffening (or shear-stiffening), therefore, appears necessary to account for the inverse correlation between tension and strain at rupture. A possible origin for shear-stiffening is that the keratin bundle network behaves not as an elastic component but as a viscoelastic Maxwell-like fluid, as a first-order approximation. If we stretch faster than the relaxation timescale of this system, all the bundles can be recruited and strain-stiffening becomes visible. However, if we stretch slower, relaxation in load-bearing bundles occurs faster than the rate at which new bundles are recruited and stiffening is not observed. To mimic this, we implemented a material model consisting of many Maxwell branches in parallel, all identical but becoming load-bearing at different strain thresholds (Fig. 6h, inset). This nonlinear viscoelastic model reproduces the experimentally observed shear-stiffening behaviour (Fig. 6h–j). After calibration, the combined rheological and bond model shows a remarkable agreement with the experimental data for Γ*, ε* and t* (Fig. 6k–m). Interestingly, simulations result in some variability (~20% of the mean) that arises from the stochastic nature of the bond model. Although the amplitude of this variability appears to depend on the strain rate, it remains far smaller than the experimental variability (~50%–75% of the mean), indicating that biological variability also contributes.

**Extended Data Fig. 9:**
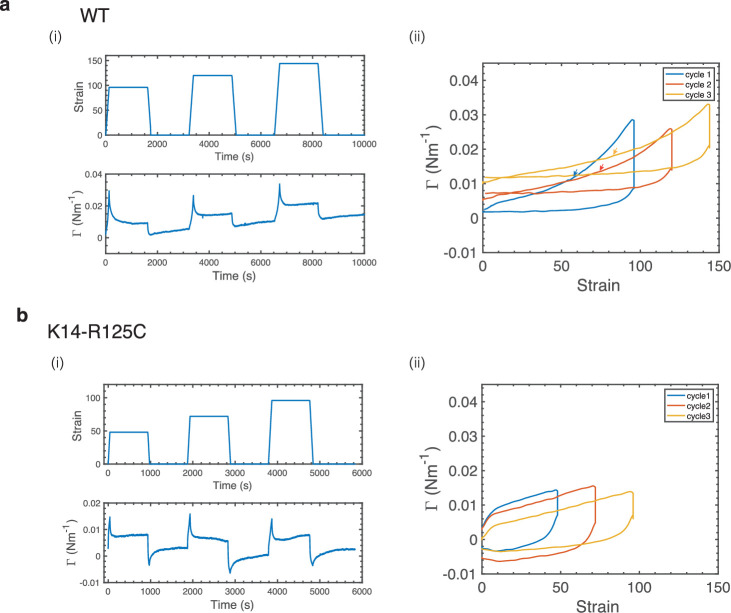
The strain stiffening threshold depends on strain history. (**a**) Wild type monolayer subjected to successive cycles of stretch and unloading at 1% s^−1^ strain rate. (i) Top: Strain imposed as a function of time. Bottom: Tension as a function of time. (ii) Tension as a function of strain for all cycles. Each cycle appears in a different colour and an arrow indicates the threshold of strain stiffening. (**b**) K14,R125C monolayers were subjected to successive cycles of stretch and unloading at 1%s^−1^ strain rate. (i) Top: Strain imposed as a function of time. Bottom: Tension as a function of time. (ii) Tension as a function of strain for all cycles. Each cycle appears in a different colour. Source data

One fundamental hypothesis of our model is that keratin filament bundles relax stress to give rise to a shear-stiffening behaviour. In vitro, keratin bundles dissipate stress through interfilament sliding^34^. One implication is that the strain-stiffening threshold observed in our experiments should depend on the strain history when the keratin network is present and we confirmed this prediction experimentally (Supplementary Results and Extended Data Fig. 9).

The relationship between Γ* and ε* (Fig. 6h) connects two qualitatively different domains. At high rates, the rupture tension is large and the rupture strain is relatively low and constant. This is a regime in which keratin bundles are massively recruited and cannot relax, forcefully breaking intercellular bonds. At slow rates, the rupture tension is smaller but the material can deform more before failing because the keratin network dissipates a significant amount of stress, delaying rupture.

## Keratins and desmosomes protect monolayers against rupture

**Extended Data Fig. 10:**
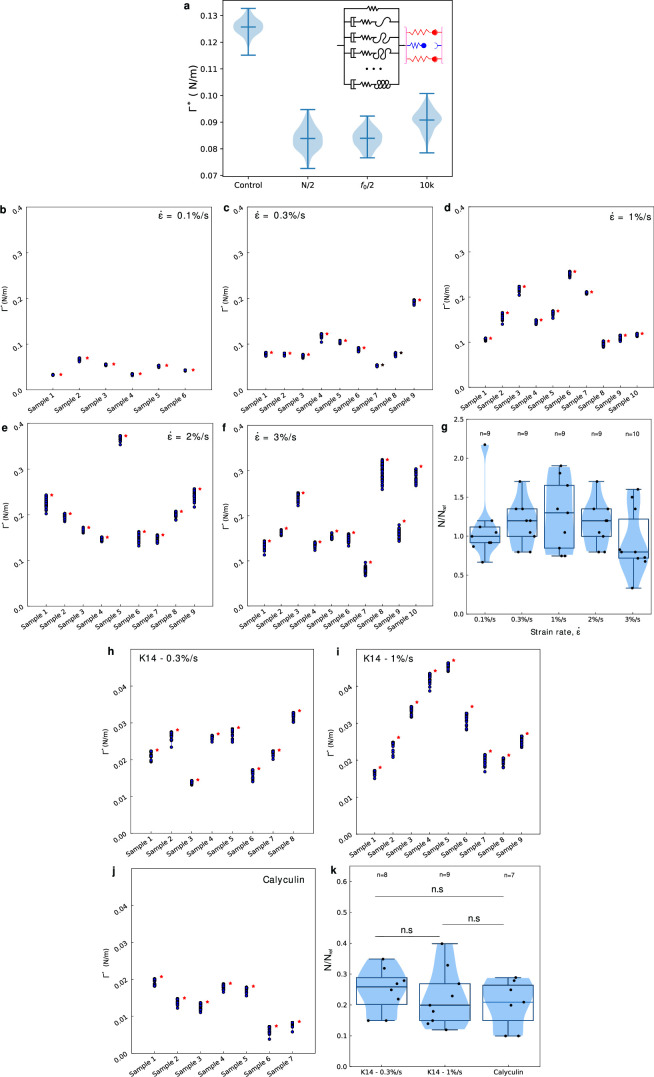
Relative linker number statistics for WT, K14 mutant and ca-lyculin treated tissues. In all box plots, the central mark indicates the median, and the bottom and top edges of the box indicate the 25th and 75th percentiles, respectively. Whiskers extend to the most extreme data points that are not outliers. (**a**) Violin plots showing the effect of varying the bond model parameters on the resulting rupture tension Γ^*^, for an imposed ramp at 1% s^−1^. The middle line shows the median and the whiskers the extremes. The outcome of the WT model parameters are compared to the outcome of the following situations: reducing the number of bonds per junction *N* by a factor 2 (N/2), reducing the force threshold f_0_ in the slip bond behaviour by a factor two (f_0/2_), or increasing the rates k_on_ and k_off,0_ by a factor 10 (10k) all lead to a significant reduction of the rupture tension. (**b**-**f**) Distributions of rupture tensions for the bond model when fitting the WT stress time series, experiment by experiment, by adjusting the linker number *N* while keeping all the other model parameters at their reference values (see Supplementary Table 7). The red asterisks show individual experimental measurements, and blue dots show simulated rupture points for the optimal N value. (**g**) Summary statistics of the resulting *N* distributions across all loading rates in WT monolayers. Each black dot represents the analysis of an individual monolayer. No significant differences between conditions were found (two-sided Wilcoxon Rank Sum test). (**h**-**j**) Similar to (**a**) but now for monolayers expressing K14,R125C (**h**-**i**) and calyculin-treated WT monolayers (**j**). (**k**) Summary distributions of N values for K14,R125C monolayers and calyculin-treated WT monolayers. Statistically significant difference: ns non-significant P > 0.05, two-sided Wilcoxon Rank Sum test. Each black dot represents the analysis of an individual monolayer. Source data

How the keratin supracellular network connected by desmosomes protects monolayers against rupture and why monolayers rupture at low stress when they increase myosin contractility remain unclear. We investigated this by probing the strength of adhesion between cells using our stochastic bond model, parameterized by the dissociation constant *k*_off_/*k*_on_ of unloaded linkers, the force dependence of the slip bond behaviour *f*_0_ and the number of linkers *N*. All of these parameters influence the strength of the monolayer (Extended Data Fig. 10a), but as the first step, we only varied *N* when adjusting our model to fit the experimental observations (Methods). For this, we subjected our junction model to the experimentally measured stress temporal evolution and adjusted *N* such that ruptures had a 50% probability of occurring before the experimentally observed Γ* and 50% after, signifying that we had reached the correct adhesive strength. This approach allows to probe the strength of intercellular adhesion between cells in experiments without necessitating the knowledge of tissue rheology with the limitation that *N* provides a relative scale rather than an absolute number of linkers.

First, we determined if the adhesion strength depends on the strain rate in wild-type (WT) monolayers. We reasoned that as the deformation is large in all the experiments, desmosomes should contribute to intercellular adhesion at all the strain rates. Consistent with this, our model revealed no significant differences in *N* (Extended Data Fig. 10b–g). Next, we compared adhesion strength in WT monolayers to those with a disrupted keratin network (K14-R125C) subjected to ramps of deformation at 1% s^−1^. *N* was almost threefold larger in control monolayers than in K14-R125C monolayers (Fig. 6n), suggesting that the disruption of the keratin network decreases the contribution of desmosomes to intercellular adhesion. Again, *N* in K14-R125C monolayers was independent of the strain rate (Extended Data Fig. 10h,i,k). Together, these data suggest that the application of strain to WT monolayers engages an additional cytoskeletal network involving keratin filaments and increases intercellular adhesion by recruiting additional bonds, probably desmosomal cadherins.

Next, we reasoned that desmosomes would not contribute in WT monolayers treated with calyculin because no deformation is applied. Therefore, intercellular adhesion strength in calyculin-treated WT monolayers should be similar to K14-R125C monolayers. When we determined the adhesion strength in calyculin-treated WT monolayers, *N* was not significantly different from that in K14-R125C monolayers subjected to deformation, but significantly lower than that in WT monolayers subjected to deformation (Fig. 6n and Extended Data Fig. 10j). An implication is that the rupture of K14-R125C monolayers in response to calyculin treatment should be similar to that of WT monolayers. Consistent with this, we found no significant differences in Γ* or t* between K14-R125C and WT monolayers treated with calyculin (Extended Data Fig. 7h–j). This suggests that the response of both K14-R125C and calyculin-treated WT monolayers is dominated by actomyosin and cadherins. This implies that rheological models calibrated for actomyosin combined with the K14/calyculin bond model should also predict the correct distribution of Γ* and ε*. Indeed, only a small adjustment of the parameters of the actomyosin rheological model is required to fit the experimental data (Fig. 6o and Fig. 6e (inset)).

## Outlook

We have characterized rupture in epithelial monolayers and shown that they are remarkably strong, withstanding several-fold increases in length before the initiation of cracking. We reveal that their mechanics at high strain and high strain rate are dominated by a supracellular network of keratin filaments linked by desmosomes. This network protects monolayers from rupture by limiting deformation through strain-stiffening and may also increase the effective intercellular adhesion when it is mechanically loaded. Finally, we show that rupture onset depends both on tissue rheology and collective bond dynamics under force. One limitation of our study is that most epithelia are bound to a basement membrane, whose mechanical properties probably influence the rupture of epithelia in vivo.

Our experiments revealed a key role for keratin filaments in tissues under large deformations: they governed strain-stiffening and protected monolayers against early rupture. Importantly, perturbing keratins directly or disrupting their interfacing to desmosomes had the same qualitative effect, signifying that it is the supracellular network that is crucial for tissue strength and strain-stiffening. These data paint a picture in which actomyosin controls tissue rheology for deformations smaller than ~50% and keratin intermediate filaments dominate for strains above 100%. Interestingly, strain-stiffening was not observed in previous work examining the response of epithelia to large deformation^28^, perhaps because of the much longer timescales over which strain was applied (several hours). The exact mechanism through which strain-stiffening arises remains to be determined. However, we hypothesize that it is due to the progressive tensile loading of keratin bundles with increasing deformation—a mechanism commonly observed in random fibre networks^35^ and proposed to play a role in tissues^36^. During physiological function, strain-stiffening may help epithelia limit how much they deform in response to an external force. Indeed, with strain-stiffening, each additional increment in deformation necessitates the application of a larger increment in force.

Our experiments show that strain-stiffening is strain rate dependent, a phenomenon known as shear-stiffening. Shear-stiffening enables the tissue to respond very differently depending on the strain rate (Fig. 6p). The tissue responds to a fast, shock-like perturbation by stiffening, thereby limiting the deformation and maximizing the force at which the material fails. However, when subjected to a slow and steady deformation, the material can tolerate very large stretch without failure. This is, to the best of our knowledge, the first characterization of such a dynamic transition with regard to rupture behaviour. The molecular mechanism underlying strain rate dependency remains unclear. However, our experiments indicate that it depends on an intact keratin network and may therefore involve the molecular turnover of proteins within the keratin–desmosome force chain or interfilament sliding within keratin bundles.

Together, our experiments and modelling linked the rupture of bonds at the molecular scale to cellular forces arising from tissue-scale deformation. Although a trade-off between force and lifetime was expected from previous work^19^, our experiments indicate that tissue rheology plays an integral part in defining rupture onset. Our data pose an intriguing question. When subjected to high strain at a high strain rate, WT monolayers strain-stiffen, and as a result, their tension is larger than monolayers with a perturbed keratin network. If the number of intercellular linkers was the same in both conditions, the force that each bond would bear would be larger in WT tissues than in keratin-compromised monolayers. As a consequence, WT tissues should rupture for lower strains than keratin-compromised ones, contrary to what we observe. One potential explanation may be that desmosomal cadherins or desmosomal proteins possess catch-bond properties, similar to E-cadherin and alpha-catenin^37,38^. At zero force, catch bonds have a short lifetime, but as the applied force increases, their lifetime lengthens to an optimum before shortening again. Under resting conditions in adherent monolayers, adherens junctions are under tension but not desmosomes^27,39^. Thus, at low strain when the keratin bundles are unloaded, desmosomal cadherins may have a short lifetime, leading to a negligible contribution to intercellular adhesion. As the strain increases, keratin bundles become progressively loaded, exerting tension on desmosomes^27^. This increases the lifetime of desmosomal cadherins as well as their contribution to load bearing. Thus, stretch would lead to both strain-stiffening and an increase in effective intercellular adhesion. Consistent with this, our model predicts that rupture tension decreases with decreasing number of linkers (Extended Data Fig. 10a) and that WT monolayers have more intercellular linkers than monolayers with disrupted keratin networks (Fig. 6n). Furthermore, transcriptomic data indicate that the number of transcripts for E-cadherin, desmoglein 2 and desmocollin 2 are comparable in MDCK cells, signifying that desmosomal cadherins could potentially provide these extra linkers (Supplementary Table 6). However, we note that other changes to the model parameters can also lead to similar changes in rupture characteristics (Extended Data Fig. 10a). Future work will be needed to thoroughly investigate the mechanism of adhesive strength reinforcement and experimentally characterize the associated physical and biological parameters.

## Methods

### Cell culture

MDCK II cells (a kind gift from Y. Fujita, Kyoto University) were cultured at 37 °C in an atmosphere of 5% CO_2_ in Dulbecco’s modified Eagle’s medium (1×) + GlutaMAX (Thermo Fisher Scientific) supplemented with 10% foetal bovine serum (Sigma-Aldrich), 2.5% of 1 M HEPES buffer (Sigma-Aldrich) and 1% penicillin–streptomycin (Thermo Fisher Scientific). Cells were passaged at 1:5 ratio every 4 days using standard cell culture protocols and disposed off after 30 passages. Mechanical experiments were performed in Leibovitz’s L15 without phenol red (Thermo Fisher Scientific) supplemented with 10% foetal bovine serum, 2.5% of 1 M HEPES buffer and 1% penicillin–streptomycin. For imaging and mechanical testing, the culture medium was exchanged for the imaging medium that consisted of Leibovitz L15 without phenol red supplemented with 10% foetal bovine serum.

To visualize junctional and cytoskeletal structures, we used stable lines expressing E-cadherin-GFP, vinculin-GFP, EPLIN-GFP and keratin 18-GFP. Details about their generation are given elsewhere^13,29^. A stable expression of the tagged protein of interest was ensured by antibiotic selection using either 250 ng ml^–1^ puromycin or 1 mg ml^–1^ G418.

To study the role of intermediate filaments, we generated cell lines stably expressing keratin 14-R125C, which leads to a disruption of the keratin network. The cDNA-encoding keratin 14-R125C-YFP was a kind gift from T. Magin (University of Leipzig) and was cloned into a retroviral vector (pTRE, Takara Clontech). The retrovirus was generated as described elsewhere^29^ and transduced into MDCK cells. After 2 weeks of selection with hygromycin (400 μg ml^–1^), cells were sorted to achieve a homogeneous level of fluorescence.

To deplete desmoplakin, we purchased shRNAs targeting dog desmoplakin in a lentiviral vector (V3LHS 302846 and 302847, pGIPZ vector, Horizon Discovery, PerkinElmer). We generated lentiviral particles following the manufacturer’s instructions, transduced MDCK cells, selected cells with puromycin and sorted the cells with flow cytometry to achieve a homogeneous knockdown. As a control, we also generated a cell line expressing non-silencing shRNA (NS) using the same methods. Protein depletion was verified by immunoblotting (Supplementary Fig. 5). The antibodies used were anti-desmoplakin (Progen, #65146, 1:200 dilution) and with anti-GAPDH as a loading control (Abcam, ab8245, 1:2,000 dilution). Appropriate horseradish-peroxidase-coupled secondary antibodies (dilution 1:10,000) were obtained from Cytiva (Cytiva NXA931). The shRNA used in this work was V3LHS302847 (Supplementary Fig. 5).

None of the cell lines in this study were found in the database of commonly misidentified cell lines maintained by the International Cell Line Authentication Committee and National Center for Biotechnology Information Biosample.

All the lines were routinely screened for the presence of mycoplasma using the MycoAlert kit (Lonza).

### RNA sequencing of MDCK cells

We used mRNA sequencing to quantify the normalized expression of mRNA transcripts for proteins in subcellular structures^13^. Briefly, to prepare the total RNA samples, MDCK cells were cultured for 3 days to reach confluence. This provides sufficient time for the junctions to mature and for the mRNA content to be regulated. Next, the total RNA was extracted using TRI reagent (Sigma-Aldrich) following the manufacturer’s protocol. Samples were processed using Illumina’s TruSeq Stranded mRNA LT sample preparation kit (p/n RS-122-2101) according to the manufacturer’s instructions. Samples were sequenced on the NextSeq 500 instrument (Illumina) using a 43-base-pair paired-end run, resulting in over 15 million reads per sample. Run data were demultiplexed and converted into files in the FASTQ format using Illumina’s bcl2fastq conversion software (v. 2.16). The files in the FASTQ format were then aligned to the CanFam3.1 assembly released by the Dog Genome Sequencing Consortium using TopHat 2.014 and then deduplicated using Picard Tools 1.79. Reads per transcript were counted using HTSeq, and the normalized expression for each mRNA transcript was estimated using the BioConductor package DESeq2.

### Stress measurement devices

The stress measurement devices were an adaptation of the force measurement device described in another study^12^. Briefly, two nickel–titanium wires (EUROFLEX) with different stiffnesses were glued into a bent glass capillary (Sutter Instruments). The arm with the stiffer wire was covered by a glass capillary to create a reference rod and two Tygon cylinders were glued to the extremities of both wires.

### Generation of suspended epithelial monolayers

Suspended epithelial monolayers were generated as described elsewhere^12^. Briefly, mechanical devices were glued into 50-mm-diameter petri dishes, placing a glass capillary underneath them to prevent contact between the device and the bottom of the petri dish, which creates friction. To maintain the distance between the rods constant during the preparation procedure, a custom-designed 3D printed plastic holder was placed in between them.

Collagen was reconstituted on ice in the following v/v proportions: 50% collagen (Cellmatrix type I-A, Nitta Gelatin), 20% 5× Dulbecco’s modified Eagle’s medium, 20% sterile water and 10% reconstitution buffer (50 mM NaOH solution in sterile water, 200 mM HEPES and 262 mM NaHCO_3_) following manufacturer’s instructions. A 10 μl drop of collagen was placed between the rods and left to solidify in a dry incubator at 37 °C for 1–1.5 h. Once a solid collagen scaffold was formed, it was rehydrated by placing an 8 μl drop of cell culture medium onto it and two 250 μl drops in the bottom of the dish. The dish was then placed for 30 min inside a humidified incubator at 37 °C. During the rehydration time, confluent flasks of MDCK cells were trypsinized for 20 min. Cells were then resuspended to a final concentration of 3 × 10^4^ cells per 10 μl. After rehydration, a 10 μl drop of the resuspended cells was placed on top of the collagen scaffold; cells were left to settle onto the collagen for 30 min inside the incubator. After this time, 8 ml of the medium was added to each petri dish, and both the V-shaped glass capillary and the holder separator were gently removed. The devices were left in the incubator for 48–72 h to allow cells to grow to confluence, covering the collagen scaffold and part of the Tygon cylinders on each test rod.

### Removal of the collagen substrate

Immediately before experimentation, a collagenase solution was prepared by mixing collagenase type II (Worthington Biochemical) with the imaging medium to reach a final concentration of 250 U ml^–1^. This solution was gradually exchanged with the cell culture medium in the petri dishes containing the devices and then left for 1 h at 37 °C to allow for full enzymatic digestion of the collagen. Finally, the collagenase solution was gradually replaced with the imaging medium. The device was then ready to be used for experiments.

### Mechanical testing procedure

The mechanical setup was mounted on the stage of an inverted microscope (Olympus IX71) and is described elsewhere^13^. The stiffer rod of the device was brought into contact with the arm of a motorized manipulator (M126-DG1) controlled through a C-863 controller (Physik Instrumente), and the softer arm of the device was attached to the tip of the force transducer (SI-KG7A, World Precision Instruments) held in position by a manual micromanipulator. Both motorized manipulator and force transducer were mounted onto magnetic plates to secure them firmly onto the microscope stage. The motorized manipulator allowed us to subject monolayers to different strains with precise control of the strain rate. Stretched monolayers exerted restoring forces on the flexible rod, deflecting the force transducer. This deflection was transformed into a voltage that was converted into a digital signal using a data acquisition system (USB-1608G, Measurement Computing) and recorded on a computer. The motorized manipulator was controlled using a custom-written code in LabVIEW (version 12.0.1f5, National Instruments). During the experiment, images of the monolayer were taken every 1 s using a ×2 objective (PLN 2X, Olympus) and a GS3-U3-60QS6M-C Point Grey camera.

### Quantification of tissue strain

Quantification of tissue strain was carried out as described in refs. ^12,13,29^. Briefly, the initial length of the monolayer *l*_0_ was determined by measuring the distance from the centre of the fixed rod to the centre of the flexible rod close to the contact point between the force transducer and the flexible rod. The motorized manipulator was then used to displace the flexible rod by distance *l*_m_. The engineering strain was then computed as *ε* = *l*_m_/*l*_0_. Our previous work has shown that the strain in the monolayer is quasi-uniform with a value tightly distributed around the value of the imposed engineering strain and that the cellular-level strain matched the tissue-level strain^29^.

### Quantification of tissue tension

In experiments using suspended epithelial monolayers, the output force measured by the transducer is in volts and it must be converted into newtons. To do this, each experiment was individually calibrated. After each experiment, monolayers were broken if they had not already failed. In these conditions, all of the force measured by the force transducer is due to the deflection of the soft wire, *d*_W_, and it can be determined using the force–deflection equation for a simple cantilever beam:

1 $$F=k{d}_{{\rm{W}}},$$

where *k* is the stiffness of the wire and is defined as

2 $$k=\frac{3EI}{{L}^{3}}.$$

Here *E* is the elastic modulus of the wire (previously determined in ref. ^12^), *I* is its moment of inertia and *L* is its length.

To determine the conversion between volts and newtons, for each experiment, we collected six voltage–deflection pairs (*V*, *d*_W_) and fitted them to a linear function. Using this procedure, we could determine the conversion factor *α* to convert volts into newtons:

3 $$\alpha =\frac{1}{V}\frac{3EI}{{L}^{3}}{d}_{{\rm{W}}}.$$

In our mechanical characterization of monolayers, we decided to approximate the tissue into a thin two-dimensional sheet and normalized the force *F* exerted on the monolayer to the average width of the monolayer before stretch *w*_0_:

4 $$\frac{F}{{w}_{0}}=\frac{\alpha V}{{w}_{0}},$$

where *w*_0_ was sampled from three positions in the monolayers and computed as

5 $${w}_{0}=\frac{{l}_{1}+2{l}_{2}+{l}_{3}}{4},$$

where *l*_1_ and *l*_3_ correspond to the width of the monolayer on each of the sides where it contacts the rods and *l*_2_ is the width at the middle point of the monolayer (Extended Data Fig. 1d, inset). This definition of width was chosen because the location of the first rupture was unpredictable and did not always coincide with where the width was the minimum.

All the tension measurements in this Article have been calculated using the initial width of the monolayer *w*_0_, as defined in equation (5), unless otherwise specified. Generally, tension measurements were smoothed with a moving average sliding fixed-time window around the time point of interest (30 points for strain rates above 1% s^−1^, 100 for 0.3% s^−1^ and 300 for 0.1% s^−1^).

Strain energy measurements were determined by integrating the area of the tension–strain curves up to the rupture point.

The entire analysis was implemented in MATLAB R2019a (MathWorks).

### Pre-tension measurements

Epithelial monolayers are intrinsically under tension due to forces generated by myosin contractility in the cells^13,16^. These intrinsic forces generate a deflection on the flexible arm of the device that corresponds to the pre-tension of the monolayer *Γ*_0_. This pre-tension was determined from two bright-field microscopy images, one acquired at the beginning of the experiment and the other at the end once the monolayer was broken. Both images were taken when no parts of the mechanical testing system contacted any of the rods of the device. A stack of both these images was generated and a region of 250 × 150 pixel^2^ was cropped around the flexible arm to measure its displacement Δ*x* using a custom-written script in MATLAB. Using Hooke’s law, the pre-tension of the monolayers is

6 $$\frac{{F}_{0}}{{w}_{0}}=-k\frac{\Delta\;x}{{w}_{0}},$$

where *k* is given by equation (2).

### Tangent modulus measurements

To determine the tangent modulus of the monolayers, we first fitted a smoothing spline to the experimental tension–strain curves. We then computed the derivative of this curve before fitting it with a smoothing spline to reduce noise. These operations were implemented in MATLAB.

### High-magnification imaging devices

Devices used for confocal imaging to determine protein localization as well as cell-shape changes were similar to those described elsewhere^18^. Briefly, a glass capillary was bent into a U shape using a small blow-torch. One of the arms of the U-shaped capillary was cut at ~5 mm from its base; in this arm, a nickel–titanium wire (EUROFLEX) was inserted to act as a hinge and covered by another piece of glass capillary. Glass coverslips (VWR) were affixed using an ultraviolet-curing glue (LOCTITE Glassbond, 447 Henkel) to the extremities of the glass capillaries to act as a substrate for cells to grow on. For precise control when stretching the monolayers, another piece of glass capillary was glued onto the end of the flexible arm at an angle to allow continuous contact with the manipulator arm (Supplementary Fig. 1).

### Confocal imaging of tissues and mechanical manipulation

Epithelial monolayers were imaged at room temperature in the imaging medium. To visualize the cell membranes, tissues were incubated for 10 min with CellMask orange membrane stain following the manufacturer’s protocol (Thermo Fisher Scientific). To visualize cell-shape changes by dye exclusion, Alexa Fluor 647-conjugated dextran (*M*_W_, 10,000; Thermo Fisher Scientific) was added to the imaging medium to a final concentration of 20 μg ml^−1^. Confocal images were acquired using a ×60 silicon objective (numerical aperture, 1.3; UPLSAPO-S, Olympus) mounted on an Olympus IX83 inverted microscope equipped with a scanning laser confocal head (Olympus FV-1200) and running the FV-10 software (FV-10ASW, version 4.2b, Olympus). Images consisted of a *Z* stack acquired at a spatial interval of 0.5–1 μm. To generate the time series, stacks were acquired every 50 s during stretching experiments and every 5 min in experiments in which monolayers were treated with calyculin. High-magnification bright-field microscopy images (Fig. 1g) were taken using a ×40 objective (Olympus, LUCPlanFL N) on an inverted Olympus IX71 microscope equipped with a GS3-U3-60QS6M-C Point Grey camera.

### Immunohistochemistry assays

To visualize the organization of the cytoskeleton and junctional proteins, we used immunostaining. For the imaging of intermediate filaments, cells were fixed with a 1:1 mix of methanol and acetone at –20 °C for 10 min. For all the other proteins, cells were fixed in 4% paraformaldehyde diluted in Dulbecco’s modified Eagle’s medium without phenol red for 15 min. Cells were then washed three times with phosphate-buffered saline (PBS) to remove any fixative. Cells were permeabilized with 0.5% Triton X-100 in PBS for 5 min at room temperature. After permeabilization, cells were washed three times with PBS and blocked with 10% horse serum in PBS for 1 h at room temperature, changing the blocking buffer every 15 min. Next, cells were incubated with primary antibodies for 2 h at room temperature in a solution of 10% horse serum in PBS. After washing three times with PBS, the cells were incubated with phalloidin 647 or 568 (Life Technologies, A22287 and A12380, 1:500 dilution) along with appropriate Alexa-conjugated secondary antibodies for 1 h at room temperature in a solution of 10% horse serum in PBS. Finally, cells were washed three times with PBS. The following primary antibodies were used: mouse anti-phospho-myosin light chain 2 (S19) (Cell Signaling 3675S, 1:100 dilution), mouse anti-E-cadherin (BD Biosciences 610181, 1:200 dilution), mouse anti-cytokeratin-18 (abcam ab668, 1:100 dilution), rabbit anti-alpha-catenin (Sigma-Aldrich C-2081, 1:200) and rabbit anti-desmoplakin (abcam ab71690, 1:100). The following secondary antibodies were used: goat anti-mouse Alexa 568 (Life Technologies, A11031, 1:200 dilution) and goat anti-rabbit Alexa 647 (Life Technologies, A27040, 1:200 dilution).

### Drug treatments

To block myosin contractility, blebbistatin (Sigma-Aldrich) was added at 50 μM concentration. To increase the myosin contractility, we inhibited phosphatases using calyculin A (Sigma-Aldrich) at 20 nM. To block actin polymerization, latrunculin B (Calbiochem) was used at 1 μM. Dimethyl sulfoxide was added to control the monolayers accordingly. Drug treatments were started 20 min before performing the ramps in deformation. To study ruptures caused by increases in internal contractility, calyculin was added at time 0 until full rupture of the monolayer. In immunostainings, drugs were added 15 min before fixation.

### Measurement of junctional protein recruitment in response to strain fluorescence intensity measurements in *XYZ*–*t* images

*Z* stacks were acquired at 1 min intervals on a confocal microscope, starting 5 min before stretch, and continuing for 3–80 min. The planes in which we measured the fluorescence intensity were selected by comparing a maximum intensity projection image with all the planes at each time point. This was done using two-dimensional cross-correlation. Alignment between the last time point before stretch and the first one after stretch was done using two-dimensional cross-correlation to ensure similar fields of view were compared. After this, a video with the optimum *Z* planes is created and saved for further processing. Segmentation of cell membranes was carried out with a packing analyser to generate a mask containing all the cell junctions. These masks were then processed in MATLAB. A second registration is performed to ensure the masks overlap correctly with each of the images of the video. Finally, the intensity of each pixel within the mask was extracted from the aligned videos and averaged to output the mean fluorescence in the region of interest at each time point. All the processing after segmentation was performed in MATLAB (Supplementary Fig. 2).

### Frog manipulation, embryo generation and maintenance

Animal procedures were approved by the Ethics Committee and Animal Welfare Body (ORBEA) of the Instituto Gulbenkian de Ciência (IGC), and complied with the Portuguese (Decreto-Lei no. 113/2013) and European (Directive 2010/63/EU) legislations. *X. laevis* oocytes were collected by inducing the superovulation of mature females with human chorionic gonadotropin (Chorulon)^40^. Briefly, oocytes were fertilized using a sperm solution in Marc’s modified ringer 0.1× medium (MMR) (10 mM NaCl, 0.2 mM CaCl_2_⋅2H_2_O, 0.2 mM KCl, 0.1 mM MgCl_2_⋅6H_2_O and 0.5 mM HEPES at pH 7.1–7.2). After de-jellying, embryos were kept in 0.1× MMR at 12–21 °C. The developmental stage of the embryos was constantly monitored and defined by following established developmental tables^41^.

### Microinjection of frog embryos

Embryos were transferred into 5% Ficoll (Sigma, P7798)/0.45× MMR (w/v) before injection and morpholinos (MO) or mRNA were injected in the dorsal and ventral blastomeres at the four-cell stage. All the microinjections were performed using calibrated glass needles mounted onto a cell microinjector (MDI, PM1000) programmed to deliver 10 nl in a pulse of 0.2 s. To visualize the nuclei and membranes of epithelial cells in vivo, 250 pg of nuclear RFP and membrane GFP mRNA were injected per blastomere. Furthermore, to knockdown keratin 8 and desmoplakin, previously validated MO^42,43^ were co-injected with membrane and nuclear markers. krt8-MO and dsp-MO were injected at a concentration of 300 μM per blastomere.

### Epidermis RNA library preparation and analysis

Epidermis of *X. laevis* was isolated and processed for RNA extraction. Briefly, RNA quality was assessed in a horse serum RNA screen tape analysis (Agilent Technologies), and mRNA libraries were prepared using SMART-Seq2 kits. Illumina libraries were generated with the Nextera standard protocol. Library quality was assessed in a fragment analyser (AATI). Sequencing was carried out in a NextSeq 500 Sequencer (Illumina) using 75 SE high-throughput kit. Sequences were extracted in the FastQ format using bcl2fastq v. 2.19.1.403 (Illumina). After filtering for ribosomal contamination, sequences were mapped against the reference genome of *X. laevis* XENLA-9.2-Xenbase.gtf (v. 9.2) (https://ftp.xenbase.org/pub/Genomics/JGI/Xenla9.2/). Gene expression tables were imported into R v. 3.6.3 to normalize the gene expression with the trimmed mean of *M* values procedure^44,45^ by using the NOISeq R package (v. 2.30.0)^46^.

### Frog immunofluorescence

Embryos were fixed with Dent’s fixative (20% dimethyl sulfoxide and 80% Methanol) for 2 h at room temperature with gentle agitation. After fixation, embryos were permeabilized with 1× PBS and 0.3% Triton X-100 (v/v) for 30 min and blocked with 10% normal goat serum in 1× PBS for 30 min at room temperature. Embryos were then incubated overnight at 4 °C with 1:50 primary antibody (keratin type II, 1h5, Developmental Studies Hybridoma Bank). Embryos were washed with 1× PBS and 0.3% Tween-20 three times and incubated with secondary antibody (anti-mouse Alexa Fluor 647) at 1:350 and DAPI solution (62249, Thermo) at 1:1,000 for 2 h at room temperature. Embryos were then washed three times and fixed with PBS and 4% formaldehyde for 10 min at room temperature before imaging in a confocal microscope (described below).

### Frog embryo mounting, microscopy and time-lapse imaging

Embryo mounting: embryos were mounted and imaged in agarose wells. Wells were shaped using 1.5-mm-outer-diameter borosilicate glass capillaries in solidifying 1% agar in 0.1× MMR. After the solidification of agar, the capillaries were carefully removed, and wells were filled with 3% methyl cellulose solution (in 0.1× MMR). Plates were then filled with 0.1× MMR and embryos were placed with the anterior part (head) pointing towards the end of the well. Fixed embryos were mounted in similar wells but filled with PBS.

Embryo extension live imaging: *Z* stacks of live embryos were acquired on a Leica Stellaris 5 upright system using either an HC APO L U-V-I ×10/0.30 NA WATER (Leica) or an HC FLUOTAR L VISIR ×25/0.95 NA WATER (Leica) objective and DPSS 561 and OPSL 488 lasers. Confocal image stacks of the embryos were acquired for 5 h at intervals of 7.5 min. The system was controlled by LAS X (Leica).

Immunofluorescence and fixed embryo imaging: *Z* stacks of live and fixed embryos were acquired on a Leica Stellaris 5 upright system using an HC FLUOTAR L VISIR ×25/0.95 NA WATER (Leica) or an HC APO L U-V-I ×40/0.80 NA WATER (Leica) objective and Diode 405, Diode 638, DPSS 561 and OPSL 488 lasers. The system was controlled by LAS X (Leica). Digital zoom was used in some cases.

### Frog image processing and data processing

Image treatment and processing: image-level adjustment, morphological segmentation, stack projection and time-lapse videos were performed using Fiji ImageJ built-in plugins (version 2.14.0/1.54k). Photoshop and Illustrator (Adobe 2023) were used to generate the final figures.

Aspect ratio calculation: the aspect ratio was calculated using frames of early and late control or MO-injected embryos (imaged as described above). For all the above-mentioned conditions, the GFP-tagged membranes of embryos constituted the input image and each individual cell was segmented using the morphological segmentation plugin in Fiji. Cells not automatically recognized by the segmentation plugin were manually segmented using the ROI manager. After proper segmentation, the aspect ratios were accessed for each cell through the minimum bounding rectangle method.

Cell strain calculation: the maximum strain was assessed at each time point in elongating live embryos by measuring the change in dimension of the cells along the anterior–posterior axis (deformation axis) using the following formula: strain (*t*) = (*X*_*t*_ – *X*_i_)/*X*_i_, where *X*_*t*_ is the cellular anterior–posterior length at time *t* and *X*_i_ is the initial cellular anterior–posterior length. All the lengths were obtained using Fiji for each time point.

### Statistics

#### Suspended monolayers

Statistical analyses were performed using MATLAB.

Box plots show the median of the distributions with a central bar, the 25th (first quartile, Q1) and 75th percentiles (third quartile, Q3) are represented by the bounding boxes, and the most extreme data points without the outliers are represented by the whiskers. Outliers are defined as being either larger than Q3 + 1.5× IQR or smaller than Q1 − 1.5× IQR, with IQR = Q3 − Q1. They appear outside the range of the whiskers and are represented by the symbol ‘+’ in red. In all the box plots, statistically significant differences are marked as follows: ns, non-significant *P* > 0.05, *P < 0.05, **P < 0.01, ***P < 0.001, ****P < 0.0001. Statistical significances were computed using a two-sided Kolmogorov–Smirnov test. The number of monolayers examined in each condition is indicated above each box plot or in the figure legend. For all the conditions examined, experiments were performed on at least two separate days.

#### Frog embryos

Data were represented and tested for normality and significance using Prism 10 (GraphPad). Datasets were tested for normality using the D'Agostino–Pearson and/or Shapiro–Wilk test. When the distributions followed a normal distribution, significance was accessed using a Student’s *t*-test (two tailed, unequal variances). When they did not, significance was calculated using a Kolmogorov–Smirnov test (two tailed, unequal variances).

### Reproducibility

The number of experimental data points (*n*) and the number of independent days (*N*) on which experiments were carried out are summarized in Supplementary Table 9.

### Computational model

#### Molecular bond dynamics model

The rupture of a junction is modelled from the dynamics of a population of *N* links, each having a slip bond behaviour, that is, a rate of binding *k*_on_ that is assumed to be constant, and a rate of unbinding *k*_off_ that is force dependent: *k*_off_ = *k*_off,0_exp(*f*/*f*_0_), where *f* is the force on the link^47^. In this implementation, the force on the junction *F* is distributed evenly over all the bound links. If, at a certain time, there is a number *n*_b_ of bound links, the force per link is *f* = *F*/*n*_b_.

The system is first initialized with a random distribution of states across the *N* links, with a probability to be bound given by *k*_on_/*k*_off,0_. The overall force over time *F*(*t*) follows precalculated curves based on the selected rheology or on the experimental data. Here it is assumed that stress is homogeneously distributed in all the intercellular junctions, and therefore, the junction force *F* is simply proportional to the tissue tension *Γ*.

To simulate the evolution of the number of bound links within the population, a small time step d*t* and discrete probabilities that junctions change state are defined at each time point based on the current force. The state of the system then evolves in a stochastic manner based on these probabilities. The model runs until the end time point is reached, or until all the links are unbound, which corresponds to the rupture time t*. The model has four parameters. We fixed two parameters in the bulk of the analysis: the number of linkers *N* = *N*_ref_ = 100 and set the ratio *k*_on_/*k*_off,0_ = 10. The values of force *f*_0_ and *k*_on_ were then varied to best match the experimental data for Γ* and ε* as a function of strain rate, once combined with the nonlinear viscoelastic model presented below. The parameter values are presented in Supplementary Table 7.

However, the model parameters predicted above do not capture the variability from one curve to the next. Therefore, we hypothesized that some biological variability existed in some of the model parameters. All the parameters influence the strength of the monolayer (Extended Data Fig. 10a). As there is considerable homology between the ectodomains 1–2 of E-cadherin and desmosomal cadherins^48,49^, we assumed that the dissociation constant was the same for all the intercellular bonds. As E-cadherins and desmosomal cadherins show very similar slip bond behaviours above a threshold force of 30 pN (refs. ^37,50^), we chose *f*_0_ to be identical for all the bonds. As a consequence, we only varied the number of bonds *N* when adjusting our model to fit the experimental observations. To estimate this variability, we considered individual experiments, and looked for the value of *N* that would best predict their rupture tension. In this situation, we only look at the data for *Γ*(*t*), and change *N* until the model predicts that 50% of the simulations fail before the experiment, that is, we look for the value of *N* such that the experimental data are the median of the model distribution. We have to use such a criterion because if the model does not fail when Γ* is reached, there is no experimental data to extrapolate the behaviour. This approach was validated by demonstrating that the values of *N* obtained on all the WT curve data were distributed about our reference value of *N*_ref_ = 100, with no systematic trend as the rate is varied (Extended Data Fig. 10b–g). When the same approach is deployed on perturbed monolayers (expressing K14-R125C or treated with calyculin), we, however, find that a significantly lower value for *N* is predicted in all these cases (Extended Data Fig. 10h–k).

#### Linear and nonlinear rheology

In the experiments and the model, the strain is controlled, and the mechanical tension *Γ*(*t*) of the epithelium is calculated using a rheological model. For a given strain rate $\dot{\epsilon }$, the strain function is given by *ε*(*t* < 0) = 0 and $\epsilon (t\ge 0)=\dot{\epsilon }t$. For the linear spring model, the tension is proportional to the strain: *Γ* = *k*_a_*ε*. For the fractional model, the calculation of the tension follows the method presented elsewhere^51^, calculated using the software package RHEOS^52^.

The nonlinear spring model corresponds to a monotonically increasing relationship between *Γ* and *ε*. To define this relationship, we assume that strain-stiffening arises from the progressive recruitment of intermediate filaments as the strain increases (in addition to the linear term defined in the previous paragraph). The strains at which fibres are recruited are modelled as a compact triangular distribution *p*_s_(*ε*) over a domain [0, 2.2], with the maximum in the middle (Supplementary Fig. 7d). These bounds are based on the range of strains for which strain-stiffening is observed in the experiments at high strain rates. The tension in the tissue is then calculated by superposition:

7 $$\varGamma (\epsilon )=k\mathop{\int}\nolimits_{\!0}^{\epsilon }{p}_{{\rm{s}}}({\epsilon }^{{\prime} })\left(\epsilon -{\epsilon }^{{\prime} }\right){\rm{d}}{\epsilon }^{{\prime} }+{k}_{{\rm{a}}}\epsilon .$$

The nonlinear viscoelastic model builds on the previous description, but assuming that once loaded, each filament behaves in a Maxwell-like manner. For a single Maxwell model with spring *k* and dashpot *η*, the response to the ramp in strain with rate $\dot{\epsilon }$ is given by $\eta \dot{\epsilon }(1-\exp (-t/\tau ))$, where *τ* = *η*/*k*. The full response is again obtained by superposition:

8 $$\varGamma (t)=\eta \dot{\epsilon }\mathop{\int}\nolimits_{\!0}^{\dot{\epsilon }t}{p}_{{\rm{s}}}({\epsilon }^{{\prime} })\left(1-\exp \left(-\frac{\dot{\epsilon }t-{\epsilon }^{{\prime} }}{\dot{\epsilon }\tau }\right)\right){\rm{d}}{\epsilon }^{{\prime} }+{k}_{{\rm{a}}}\dot{\epsilon }t.$$

The distribution *p*_s_ is adjusted to mimic the high-strain-rate limit, identical to the nonlinear spring model (Supplementary Fig. 7d). The extra parameter *η* (or equivalently *τ*) is adjusted to account for the observed timescale associated with the shear-stiffening behaviour. The parameter values used in the different rheological models are presented in Supplementary Table 7.

### Reporting summary

Further information on research design is available in the Nature Portfolio Reporting Summary linked to this article.

## Online content

Any methods, additional references, Nature Portfolio reporting summaries, source data, extended data, supplementary information, acknowledgements, peer review information; details of author contributions and competing interests; and statements of data and code availability are available at 10.1038/s41563-024-02027-3.

## Supplementary information

Supplementary Information Supplementary Figs. 1–7, Tables 1–9, captions for Supplementary Videos 1–14, Results and discussion.

Reporting Summary

Supplementary Video 1 Suspended MDCK WT monolayer subjected to a ramp in deformation at 1% s^−1^. Time 0 corresponds to the onset of stretch. Time is in seconds. Scale bar, 500 μm.

Supplementary Video 2 Suspended MDCK E-cadherin-GFP monolayer subjected to constant stretch imaged by phase contrast microscopy at ×40 magnification. Time is in seconds. Scale bar, 10 μm.

Supplementary Video 3 Suspended MDCK WT monolayer treated with calyculin 20 nM. Calyculin is added at time 0. Time is in seconds. Scale bar, 500 μm.

Supplementary Video 4 Suspended MDCK WT monolayer pretreated with blebbistatin 50 μM for 30 min. Blebbistatin is added at time 0. Time is in seconds. Scale bar, 500 μm.

Supplementary Video 5 Suspended MDCK WT monolayer treated with calyculin. The monolayer was previously incubated with blebbistatin for 30 min (Supplementary Video 4). Calyculin is added at time 0. Time is in seconds. Scale bar, 500 μm.

Supplementary Video 6 Suspended MDCK WT monolayer treated with blebbistatin 50 μM for 20 min and subjected to a ramp in deformation at 1% s^−1^ starting at time 0. Time is in seconds. Scale bar, 500 μm.

Supplementary Video 7 Suspended MDCK WT monolayer treated with DMSO for 20 min and subjected to a ramp in deformation at 1% s^−1^ starting at time 0. Time is in seconds. Scale bar, 500 μm.

Supplementary Video 8 Suspended MDCK WT monolayer treated with calyculin 20 nM for 20 min and subjected to a ramp in deformation at 1% s^−1^ starting at time 0. Drug added at time 0. Time is in seconds. Scale bar, 500 μm.

Supplementary Video 9 Suspended MDCK WT monolayer treated with latrunculin B 1 μM for 20 min and subjected to a ramp in deformation at 1% s^−1^ starting at time 0. Time is in seconds. Scale bar, 500 μm.

Supplementary Video 10 Suspended MDCK monolayer overexpressing keratin 14-R125C subjected to a ramp in deformation at 1% s^−1^. Time 0 corresponds to the onset of stretch. Time is in seconds. Scale bar, 500 μm.

Supplementary Video 11 Suspended MDCK NS-shRNA monolayer subjected to a ramp in deformation at 1% s^−1^. Time 0 corresponds to the onset of stretch. Time is in seconds. Scale bar, 500 μm.

Supplementary Video 12 Suspended MDCK DSP-shRNA monolayer subjected to a ramp in deformation at 1% s^−1^. Time 0 corresponds to the onset of stretch. Time is in seconds. Scale bar, 500 μm.

Supplementary Video 13 Body-axis elongation in a *X. laevis* embryo. The anterior–posterior axis is oriented vertically. Lateral epidermis cells expressing a GFP membrane marker (black) elongate as development proceeds (see the right side of the embryo). Frame interval, 7.5 min; 20 frames are shown. Scale bar, 500 μm. Representative example of three independent experiments.

Supplementary Video 14 Effect of the impact of desmoplakin and keratin 8 knockdown on cell shape during body-axis elongation. Cells express a GFP membrane marker (black). Left: cells within the control embryos. Middle: cells within an embryo microinjected with MO against desmoplakin and keratin 8 displaying a mild phenotype. Right: cells with an embryo microinjected with MO against desmoplakin and keratin 8 displaying a severe phenotype. Frame interval, 7.5 min; 20 frames are shown. Scale bar, 50 μm. Representative of three independent experiments.

## Source data

Source Data Fig. 1 Data for Fig. 1 (statistics included, if applicable).

Source Data Fig. 2 Data for Fig. 2 (statistics included, if applicable).

Source Data Fig. 3 Data for Fig. 3 (statistics included, if applicable).

Source Data Fig. 4 Data for Fig. 4 (statistics included, if applicable).

Source Data Fig. 5 Data for Fig. 5 (statistics included, if applicable).

Source Data Fig. 6 Results of the computational simulations plotted in Fig. 6 and link to the UCL repository.

Source Data Extended Data Fig. 1 Data for Extended Data Fig. 1 (statistics included, if applicable).

Source Data Extended Data Fig. 2 Data for Extended Data Fig. 2 (statistics included, if applicable).

Source Data Extended Data Fig. 3 Data for Extended Data Fig. 3 (statistics included, if applicable).

Source Data Extended Data Fig. 4 Data for Extended Data Fig. 4 (statistics included, if applicable).

Source Data Extended Data Fig. 5 Data for Extended Data Fig. 5 (statistics included, if applicable).

Source Data Extended Data Fig. 6 Data for Extended Data Fig. 6 (statistics included, if applicable).

Source Data Extended Data Fig. 7 Data for Extended Data Fig. 7 (statistics included, if applicable).

Source Data Extended Data Fig. 8 Data for Extended Data Fig. 8 (statistics included, if applicable).

Source Data Extended Data Fig. 9 Data for Extended Data Fig. 9 (statistics included, if applicable).

Source Data Extended Data Fig. 10 Results of the computational simulations plotted in Extended Data Fig. 10 and link to the UCL repository.

## Data Availability

The data that support the findings of this study are included in the article and its Supplementary Information and are available via the UCL research data repository (https://rdr.ucl.ac.uk/) at 10.5522/04/21407160 or from the corresponding authors upon request. Source data are provided with this paper.

## Code availability

All the computational models developed in this work were implemented in the Julia programming language. The rupture stress, strain and time for all the rheological models were calculated using the adhesion model available via GitHub at https://github.com/ComputationalMechanobiology/CellAdhesion.jl (commit ID: 8cc2ea). The stress and strain curves for the fractional viscoelastic models were calculated using RHEOS v0.9.4, which is available as a Julia package and via GitHub at https://github.com/JuliaRheology/RHEOS.jl. The stress and strain curves for the nonlinear models were calculated using code available via GitHub at https://github.com/ComputationalMechanobiology/IFTissueRheol (commit ID: cb750ca).
