# Supplementary material for: Rupture strength of living cell monolayers
Source: Nat Mater. 2024 Oct 28;23(11):1563–74. doi: 10.1038/s41563-024-02027-3 (PMC11525174; doi:10.1038/s41563-024-02027-3)
Supplement: Supplementary file 1 — Supplementary Figs. 1–7, Tables 1–9, captions for Supplementary Videos 1–14, Results and discussion. [file 41563_2024_2027_MOESM1_ESM.pdf]

nature materials

Supplementary information https://doi.org/10.1038/s41563-024-02027-3

Rupture strength of living cell monolayers

In the format provided by the authors and unedited

Supplementary Material

Supplementary Movies

Supplementary Video 1. Suspended MDCK wild-type monolayer subjected to a ramp in deformation at 1% s^{-1}. Time 0 corresponds to the onset of stretch. Time is in seconds. Scale bar is 500 µm.
Supplementary Video 2. Suspended MDCK E-cad-GFP monolayer subjected to constant stretch imaged by phase contrast microscopy at 40X high magnification. Time is in seconds. Scale bar is 10 µm.
Supplementary Video 3. Suspended MDCK wild-type monolayer treated with calyculin 20 nM. Calyculin is added at time 0. Time is in seconds. Scale bar is 500 µm.
Supplementary Video 4. Suspended MDCK wild-type monolayer pretreated with blebbistatin 50 µM for 30 min. Blebbistatin is added at time 0. Time is in seconds. Scale bar is 500 µm.
Supplementary Video 5. Suspended MDCK wild-type monolayer treated with calyculin. The monolayer was previously incubated with blebbistatin for 30 min as shown in **Supplementary Video 4**. Calyculin is added at time 0. Time is in seconds. Scale bar is 500 µm.
Supplementary Video 6. Suspended MDCK wild-type monolayer treated with blebbistatin 50 µM for 20 min and subjected to a ramp in deformation at 1% s^{-1} starting at time 0. Time is in seconds. Scale bar is 500 µm.
Supplementary Video 7. Suspended MDCK wild-type monolayer treated with DMSO for 20 min and subjected to a ramp in deformation at 1% s^{-1} starting at time 0. Time is in seconds. Scale bar is 500 µm.
Supplementary Video 8. Suspended MDCK wild-type monolayer treated with calyculin 20 nM for 20 min and subjected to a ramp in deformation at 1% s^{-1} starting at time 0. Drug added at time 0. Time is in seconds. Scale bar is 500 µm.
Supplementary Video 9. Suspended MDCK wild-type monolayer treated with Latrunculin A 1µM for 20 min and subjected to a ramp in deformation at 1% s^{-1} starting at time 0. Time is in seconds. Scale bar is 500 µm.
Supplementary Video 10. Suspended MDCK monolayer overexpressing Keratin 14-R125C subjected to a ramp in deformation at 1% s^{-1}. Time 0 corresponds to the onset of stretch. Time is in seconds. Scale bar is 500 µm.
Supplementary Video 11. Suspended MDCK non-silencing shRNA monolayer subjected to a ramp in deformation at 1% s^{-1}. Time 0 corresponds to the onset of stretch. Time is in seconds. Scale bar is 500 µm.
Supplementary Video 12. Suspended MDCK Desmoplakin-shRNA monolayer subjected to a ramp in deformation at 1% s^{-1}. Time 0 corresponds to the onset of stretch. Time is in seconds. Scale bar is 500 µm.
Supplementary Video 13. Body axis elongation in a *Xenopus laevis* embryo. The anteroposterior (AP) axis is oriented vertically. Lateral epidermis cells expressing a GFP membrane marker (black) elongate as development proceeds, see right hand side of the embryo. Frame interval 7.5 minutes and 20 frames are shown. Scale bar is 500 µm. Representative example of three independent experiments.
Supplementary Video 14. Effect of the impact of desmoplakin and keratin 8 knockdown on

cell shape during during body axis elongation. Cells express a GFP membrane marker (black). Left: cells within control embryos. Middle: cells within an embryo microinjected with morpholinos against desmoplakin and keratin 8 displaying a mild phenotype. Right: cells with an embryo microinjected with morpholinos against desmoplakin and keratin 8 displaying a severe phenotype. Frame interval 7.5 minutes and 20 frames are shown. Scale bar is 50 µm. Representative of three independent experiments.

Supplementary results

Strain stiffening does not depend on mechanotransductory processes

Strain stiffening might arise from mechanotransductory processes or from the intrinsic organisation of the cytoskeleton and cells within monolayers. Stiffening occurred for strain rates above 0.3% s^{-1} with the most pronounced observed for 3% s^{-1}, signifying that a hypothetical mechanotransductory process should take place over durations shorter than $\sim$ 300 seconds.

Strain-stiffening and fracture are independent of actomyosin

Actomyosin is arguably the main determinant of cell shape and a key player in cell mechanics. In tissues subjected to low strain (less than 30%, before strain stiffening is observed), the actomyosin cortex controls monolayer rheology [13] and increases in cortical contractility lead to stress stiffening [16]. Therefore, we examined the role of myosin in strain stiffening by repeating our ramp deformation experiments at 1% s^{-1} for monolayers in which myosin contractility had been inhibited or enhanced (**Extended Data Figure 5b**, **Supplementary Figure 4d**). For this, we blocked myosin activity with blebbistatin, and we increased phospho-myosin levels by treating monolayers with calyculin for 20 minutes, a duration sufficient to increase pre-tension Γ_0 significantly but not to cause rupture (**Fig. 2c, Supplementary Videos 6, 8**). Neither treatment affected strain stiffening (**Extended Data Figure 5b**). Next, to examine the role of F-actin, we treated monolayers with latrunculin, an inhibitor of actin polymerisation that leads to global loss of F-actin (**Supplementary Figure 4f, Supplementary Video 9**). Similar to myosin, F-actin appeared to play no role in strain-stiffening (**Extended Data Figure 5b**, **Supplementary Figure 4d**). Interestingly, none of these treatments affected the rupture characteristics of monolayers (**Extended Data Figure 5c - e, Supplementary Figure 4a - c**). Hence, the strength of epithelia appears independent of actomyosin.

Strain-stiffening requires the keratin supracellular network

We next investigated the contribution of keratin filament networks to strain-stiffening by computing the tangent modulus in K14-R125C and DSP-shRNA monolayers subjected to ramps at 1% s^{-1} (**Fig. 5a-d**). These presented two main features that differed from controls. First, in both perturbations, there was no increase in the tangent modulus with strain in contrast to controls (**Fig. 5b, d**). This indicated that both the keratin network and its attachment to cell junctions are necessary for monolayers to strain-stiffen. Second, in the DSP-shRNA monolayers, we detected larger values of the tangent modulus at low strains compared to control (**Fig. 5d**, **Extended Data Figure 6d**). Although this was surprising, we reasoned this may arise because of a compensatory mechanism. For example, when tight junction proteins ZO-1 and ZO-2 are depleted, cells become more contractile[48].

To investigate the molecular mechanism underlying the increase in tangent modulus, we immunostained DSP-shRNA and control monolayers against phospho-myosin (pMLC), F-actin, and E-cadherin. In DSP-shRNA monolayers, we visually observed an increase in pMLC and F-actin

as well as E-cadherin (**Extended Data Figure 6e**, **7g** respectively). This suggests that an increase in myosin contractility underlies the higher tangent modulus of DSP-shRNA monolayers at low strain, consistent with the stress-stiffening behaviour observed at low strain in response to short duration calyculin treatment reported in our previous work [16]. However, DSP-shRNA monolayers were more fragile at high strain despite having more F-actin, pMLC and E-cadherin (**Extended Data Figure 6e**, **7g**).

When we perturbed actomyosin organisation, we could not observe any impact on strain stiffening (**Extended Data Figure 5**) or the rupture characteristics of monolayers **(Extended Data Figure 5c - e)**, implying that the structure that controls these properties is not affected by treatment with latrunculin, blebbistatin or calyculin. Therefore, we verified by immunostaining that these drugs did not affect the keratin network **(Extended Data Figure3i, Supplementary Figure 4e)**. Overall these results confirm that strain-stiffening is dependent on the presence of a supracellular keratin network, pointing to its key role in governing the mechanical response of monolayers at high strains.

Finally, we investigated if intermediate filament perturbation affected the strain rate dependency of rupture tension and strain (**Extended Data Figure 7a - d**). For this, we compared K14-R125C monolayers subjected to ramps at two different strain rates, 1% s^{-1} and 0.3% s^{-1}. Neither rupture tension nor rupture strain changed with strain rate (**Extended Data Figure 7a, b**), in contrast to control monolayers (**Fig. 3a, b**). When we plotted these experiments in rupture tension- rupture strain or rupture tension- rupture time plots, we found that they presented a trend very different from the ramp experiments performed on WT monolayers (**Fig. 5e, f**).

Keratin networks prevent epithelial rupture in embryogenesis

To determine the importance of keratin networks and desmosomes in physiological conditions, we examined their role in the epidermis of *Xenopus laevis* during body axis elongation, a process occurring at a very low strain rate driven by long range tensile forces [**?**, 49, 50]. We first characterised strain in wild type embryos to determine regions exposed to high strain, which may be affected by perturbation to keratins and desmosomes. For this, we microinjected fluorescent markers to visualise the membrane and the nucleus of epidermal cells by live microscopy. Our temporal analyses of cell morphology revealed that lateral epidermal cells undergo large deformation along the anteroposterior axis (**Extended Data Figure 8a, b, d**; **Supplementary Video 13**). After identifying the relevant desmosomal and keratin proteins expressed in the elongating lateral epidermis (**Supplementary Figure 6**), we analysed the impact of depleting desmoplakin and keratin 8 by microinjecting anti-sense oligonucleotides previously validated in *Xenopus laevis* [40, 41]. Cells in control embryos became visibly elongated in the anteroposterior axis between the beginning and end of body axis elongation (**Extended Data Figure 8b**), leading to cells with a significantly larger aspect ratio at late stages (**Extended Data Figure 8d**). Over the course of body axis elongation, average cell strain increased at a constant rate, reaching ∼40% by the end of this process (**Extended Data Figure 8e).** Whereas a clear keratin network was visible within the cells of wild-type embryos **(Extended Data Figure 8f)**, it became less well defined and

no longer appeared connected across cells in knockdown embryos (**Extended Data Figure 8f**, compare the membrane and the cytokeratin localisations). Morphologically, cells within knockdown tissues had a significantly lower aspect ratio than wild-type cells at the same stage (**Extended Data Figure 8d**), perhaps because the keratin network no longer transmitted long range tensile forces. Consistent with this idea, cell strain remained low over the course of body axis elongation (**Extended Data Figure 8e**, **Supplementary Video 14** middle). When the keratin supracellular network was perturbed, cells often detached from their neighbours creating small defects and these occasionally gave rise to larger cracks (**Extended Data Figure 8c**, **Supplementary Video 14** right). Crack area remained much smaller than in suspended monolayers, likely because of adhesion to underlying cell layers limits crack growth. Although knockdown embryos developed until late neurula stages, they died at later larval stages. In summary, a supracellular network of keratins linked by desmosomes is critical during development but the presence of additional boundary conditions on the basal side may limit crack growth.

A computational model for examining the interplay between rheology and adhesion

The onset of rupture is associated with the separation of cell junctions. At the molecular level, this implies unstable dynamics with bonds dissociating more frequently than they associate. Simple models of interfaces linked by dynamic bonds have already demonstrated that a molecular slip bond behaviour may lead to a finite time of separation that decreases with the mechanical tension applied to the system [19, 20]. Such a trade-off between time-to-fracture and applied mechanical force is consistent with our observation that monolayers reach higher rupture tension Γ^* and shorter rupture times t^* at large strain rates (**Fig. 3a, c**). However, we also observed that the strain at the onset of fracture decreased with strain rate (**Fig. 3b**): the higher the rupture tension, the lower the strain at rupture (**Fig. 3e**). Remarkably, this qualitative trend is not present when the intermediate filament network is disrupted and the shear-stiffening behaviour of the monolayer is abrogated (**Fig. 5e**). Although separation of a junction is primarily controlled by the tension it is subjected to, this tension results from a multitude of processes, ranging from externally imposed deformations or forces due to active contractile behaviours. Furthermore, the boundary conditions influence the resulting mechanical state of the tissue. The onset of monolayer rupture therefore likely involves the interplay between the tissue-scale monolayer rheology and the molecular-scale dynamics of bonds. We hypothesise here that this interplay is responsible for the range of behaviours reported in this study.

A stochastic slip bond dynamics model inspired by the work of [19] and [20] combined with a range of material models enabled us to explore the qualitative and quantitative agreement of this hypothesis with our data (see methods). In essence, two surfaces representing an intercellular junction are connected by a population of N independent linkers that can exist in two states, bound or unbound (**Fig. 6a**). They bind at a fixed rate k_{on}, but unbind at a rate k_{off} that increases exponentially with mechanical load with a scale f_0. In our implementation, all bound links bear an equal share of the tension applied to the surfaces. Rupture is assumed to be triggered when the system transitions to a state where all links detach (**Fig. 6b**). When a constant force is applied to a junction, we find as expected that the larger the applied force is, the shorter the time to rupture (**Fig. 6c**).

In our experiments, the force is not constant but instead results from ramps of deformation applied at different rates. The tension in the tissue for a given strain rate is a function of time that depends on the material's rheology. For a range of strain rates and material models, we can calculate the temporal evolution of the applied tension and the bond dynamics can then be simulated a large number of times to get statistics for the rupture parameters Γ^* and t^*. The corresponding value of ε^* is then determined using the rheological model.

To grasp the role of rheology in the fracture behaviour, we first considered the case of a linear elastic relationship between stress and strain, which corresponds to the low strain rate limit identified in [13, 46] for relatively small deformations (less than 30%). As expected, the rupture load increases with strain rate (**Supplementary Figure 7a**), but so does the strain at rupture (**Fig. 6d**); this arises because stress and strain are proportionally related to each other through the constitutive equation of the material. A more complex material model takes into account the rich time-dependent linear rheological behaviour that we previously validated at low deformation [46] and that is controlled by actomyosin [13] (**Supplementary Figure 7b**). However, even in this case, the relationship between rupture strain and strain rate remains a monotonically increasing function (**Fig. 6**e).

The strain stiffening threshold depends on strain history

One fundamental hypothesis of our model is that keratin filament bundles relax stress to give rise to a shear stiffening behaviour. In vitro, keratin bundles have been shown to dissipate stress through interfilament sliding [34]. One implication is that the strain stiffening threshold should depend upon the strain history when the keratin supracellular network is present.

To test this, we subjected monolayers to a ramp of deformation to 100% strain at 1% s^{-1} strain rate, maintained them stretched for 20 min, and returned them to their initial length. We then repeated this procedure increasing the deformation to 125% and then 150% in cycles 2 and 3 (**Extended Data Figure 9a**-(i)). If our rheological model is conceptually correct, we expect that the threshold strain for stiffening will increase in cycles 2 and 3 to a strain close to the holding strain in the previous cycle because some interfilament sliding has taken place (or equivalently because dashpots have had time to flow in our rheological model, **Fig. 6h**), effectively adding extra length to the slack. In our experiments, the monolayers displayed strain stiffening around 50% deformation in cycle 1 and their stress relaxed by $\sim$ 60% over a period of $\sim$ 10 min (**Extended Data Figure 9a**-(i)). Consistent with our hypothesis, strain stiffening occurred around 75% in cycle 2 and around 85% in cycle 3 (arrows, **Extended Data Figure 9a**-(ii)). Stress relaxation with similar characteristics to the first cycle could be observed while the monolayers were maintained stretched in cycles 2 and 3 (**Extended Data Figure 9a**-(i)). In contrast, no such behaviours were observed in monolayers expressing K14-R125C (**Extended Data Figure 9b**), confirming that it was linked to the presence of an intact keratin supracellular network. Overall, these experiments suggested that our rheological model was qualitatively correct.

Supplementary discussion

We have characterised fracture in epithelial monolayers and showed that they are remarkably strong, withstanding several-fold increases in length before the initiation of cracking. Remarkably, increases in cell contractility also ruptured monolayers in the absence of any applied deformation. By systematically varying strain rate, we reveal a trade-off between rupture tension and rupture time with large tensions leading to short lifetimes. Importantly, we unravelled a role for the supracellular keratin filament network in controlling the rheology and strength of tissues both in vitro and in vivo. Finally, using computational modelling, we show that rupture onset depends strongly on tissue rheology and collective bond dynamics under force.

Our experiments revealed a key role for keratin intermediate filaments in the response of tissues to large deformations: they governed strain stiffening and protected monolayers against early rupture. Importantly, perturbing the keratin intermediate filaments directly or indirectly by disrupting their interfacing to desmosomes had the same qualitative effect, signifying that it is the supracellular network linking individual cellular keratin networks that is crucial for tissue strength and strain stiffening. Consistent with our in vitro experiments, combined depletion of keratin 8 and desmoplakin in Xenopus embryos fragilised the epidermis, leading to the formation of cracks during body axis elongation. Whereas the actomyosin cytoskeleton is central to cell and tissue mechanics at low strain [51, 52, 13], its perturbation did not affect strain stiffening nor the strength of tissues. Most epithelia are bound to a basement membrane and, as a consequence, rupture of the epithelium *in vivo* will likely be influenced by the mechanical properties of the basement membrane. Future work will be necessary to characterise the strength epithelium-basement membrane composite.
These data paint a picture in which the actomyosin cytoskeleton controls tissue rheology for deformations smaller than $\sim 50\%$ and keratin intermediate filaments dominate for strains above 100%, consistent with previous work [28]. This transition was manifested by a progressive stiffening of monolayers for strain above 50%. Interestingly, such a mechanism was not observed in previous work examining the response of epithelia to large deformation [28], perhaps because of the much longer time-scales over which strain was applied (tens of hours) resulting in a very low strain rate. The exact mechanism through which strain stiffening arises remains to be determined. However, we hypothesise that it is due to progressive tensile loading of keratin bundles with increasing deformation, a mechanism commonly observed in random fibre networks [35, 53, 54] and proposed to play a role in tissues [36]. Previous work has shown that, at low strain, keratin intermediate filament bundles appear wavy but straighten when deformation increases, indicative of a transition from unloaded to loaded [28, 29]. Within cells, the keratin network radiates from the perinuclear region towards the cell junctions [55] (**Fig. 4a, e**). In response to uniaxial stretch, keratin filaments aligned with the direction of stretch will straighten first and, as deformation increases, filaments at increasingly larger angles from this direction will straighten. Thus, more and more mechanical elements bear load as deformation increases. During physiological function, strain stiffening may help epithelia limit how much they deform in response to an external force. Indeed, with strain stiffening, each additional increment in deformation necessitates the application of a larger increment in force.

Our experiments show that strain stiffening is strain rate dependent. This signifies that, at low strain rate, strain stiffening is absent and this may allow tissues to deform substantially. Experi-

ments in which we subjected monolayers to cycles of increasingly large deformation suggested that the keratin network remodels over tens of minutes to dissipate stress (**Extended Data Figure 9**). The molecular mechanism underlying strain rate dependency remains unclear; however, it might involve molecular turnover of proteins within the keratin-desmosome force chain or sliding between filaments within the keratin bundles. Indeed, experiments examining the mechanical response of keratin bundles in cells and in vitro both indicate the presence of sliding between filament subunits in response to stretch [56, 34]. Alternatively, turnover of proteins within the cytoskeleton has been shown to dissipate stress. At low strain, cells and tissues dissipate stress on minute time-scales due to turnover of actomyosin [57, 13]. Monolayers subjected to 0.1% s^{-1} do not strain-stiffen, indicating that potential dissipatory turnover mechanisms act faster than the $\sim$ 10 - 20 minutes necessary to reach the strain magnitudes at which stiffening is observed. While keratins and desmoplakin turn over with characteristic times of $\sim$ 1h [58, 13], the desmosomal cadherin desmoglein 2 is reported to turn over on a time-scale of $\sim$ 20 minutes [59] and plakophilin turns over substantially within 5 minutes [60]. Future work will be necessary to determine which of interfilament sliding or molecular turnover contributes most to stress dissipation in keratin networks.

Together our experiments and modelling allowed to link rupture of bonds at the molecular-scale to cellular forces arising from tissue-scale deformation of the monolayer. While the trade-off between force and lifetime was expected from previous work [19], our experiments indicate that tissue rheology plays an integral part in defining rupture onset. Remarkably, our model shows that prediction of rupture tension and strain necessitates the implementation of a realistic tissue rheology that incorporates shear stiffening. Our data poses an intriguing question. At high strain imposed at sufficiently high strain rate, wild-type monolayers strain stiffen and, as a result, their tension is larger than monolayers with a perturbed keratin network. If the number of adhesion proteins linking the cells is the same in both conditions, the force that each bond must bear will be larger in wild-type tissues than in K14-R125C monolayers. As a consequence, we would expect wild-type tissues to rupture at lower strains than keratin compromised ones, the contrary of what we observe. One potential explanation may be that desmosomal cadherins or desmosomal proteins possess catch-bond properties, similar to E-cadherin and alpha-catenin [37, 38]. At zero force, catch bonds have a very short lifetime but, as applied force increases, their lifetime grows to an optimum before decreasing again. Work using FRET tension sensors has shown that, under resting conditions in adherent monolayers, adherens junctions are under tension but that desmosomes are not [39, 27]. Thus, at low strain when keratin filaments are unloaded, desmosomal cadherins may not sense any force and have a lifetime so short that their contribution to intercellular adhesion is minimal. As strain increases, keratin filaments become progressively loaded, exerting tension on desmosomes [27] and potentially increasing the lifetime of desmosomal cadherins as well as their contribution to load bearing. Thus, stretch would lead to both strain stiffening and an increase in the effective intercellular adhesion. Consistent with this hypothesis, our computational model of intercellular junctions predicts that rupture stress decreases with decreasing number of bonds (**Extended Data Figure 10a**) and that wild-type monolayers have more intercellular adhesive bonds than monolayers with disrupted keratin networks (**Fig. 6n**). Furthermore, transcriptomic data indicates that the number of transcripts for E-cadherin, desmoglein 2, and desmocollin 2 are comparable in MDCK cells, signifying that desmosomal cadherins could potentially provide these extra bonds (**Supplementary Table 6**). Conversely, when stress arises from an increase in

myosin contractility rather than deformation, no additional bond recruitment takes place in wild-type monolayers (**Fig. 6n**). Together these data suggest that the effective number of intercellular adhesion bonds increases when deformation is applied to monolayers in which a supracellular keratin network is present. However, we note that other changes to the model parameters (such as the slip bond characteristic force f_0 or the dissociation constant) can also lead to similar changes in rupture characteristics (**Extended Data Figure 10a**). Future work will be needed to thoroughly investigate the mechanism of adhesive strength reinforcement and experimentally characterise the associated physical and biological parameters.

From a physiological point of view, the shear-stiffening behaviour enables the tissue to respond very differently to mechanical perturbations depending on the strain rate (**Fig. 6p**). The tissue responds to a fast, shock-like, perturbation by stiffening and increasing effective adhesive strength, therefore limiting the deformation and maximising the force at which the material fails. However, when subjected to a slow and steady deformation, the material can tolerate very large stretch without failure. This is, to our knowledge, the first characterisation of such a dynamic transition with regards to rupture behaviour but the exact biophysical mechanisms underlying it remain unclear. Our computational model assumes that the number of intercellular adhesive proteins within a junction does not change during deformation. Yet, imaging reveals that the height of intercellular junctions visibly decreases with strain (**Fig. 1f**). How changes in junction shape and size affect the number of E-cadherins and desmosomal cadherins engaged in intercellular adhesion as well as their stability is unclear. Previous work examining shrinkage of intercellular junctions in response to myosin contractility have reported an increase in the density of E-cadherins, suggesting that the overall number of E-cadherin links present at the junction remains constant [61]. In addition, application of stress can modulate the stability of intercellular junctions by decreasing cadherin turnover [62], increasing the life time of proteins within the cadherin-catenin complexes (e.g. E-cadherin and alpha-catenin, [37, 38, 63]), and recruiting proteins to reinforce intercellular junctions (e.g. vinculin) [64]. Finally, our study further suggests that application of deformation increases the effective number of intercellular bonds by putting the keratin intermediate filament network and desmosomes under mechanical load. Thus, in principle, deformation could lead to changes in the number of intercellular bonds N, their dissociation constant, and their sensitivity to force f_0. Further work will therefore be necessary to determine the exact contribution of each of these mechanisms to the overall strength of tissues and its modulation by strain application.

In summary, our work has shown that the mechanics of tissues at high strain and high strain rate is dominated by a supracellular network of keratin intermediate filaments linked by desmosomes. This network protects monolayers from rupture by limiting deformation through strain stiffening and may also increase effective intercellular adhesion when it is mechanically loaded. One implication of our work is that the rupture characteristics of monolayers expressing K14-R125C reflect the strength of actomyosin and adherens junctions, while the rupture characteristics of monolayers treated with latrunculin reflect the strength of keratins and desmosomes. In the present study, we only examined the onset of rupture and future work will be needed to investigate crack propagation in the plane of the tissue.

Appendix

Supplementary Figures

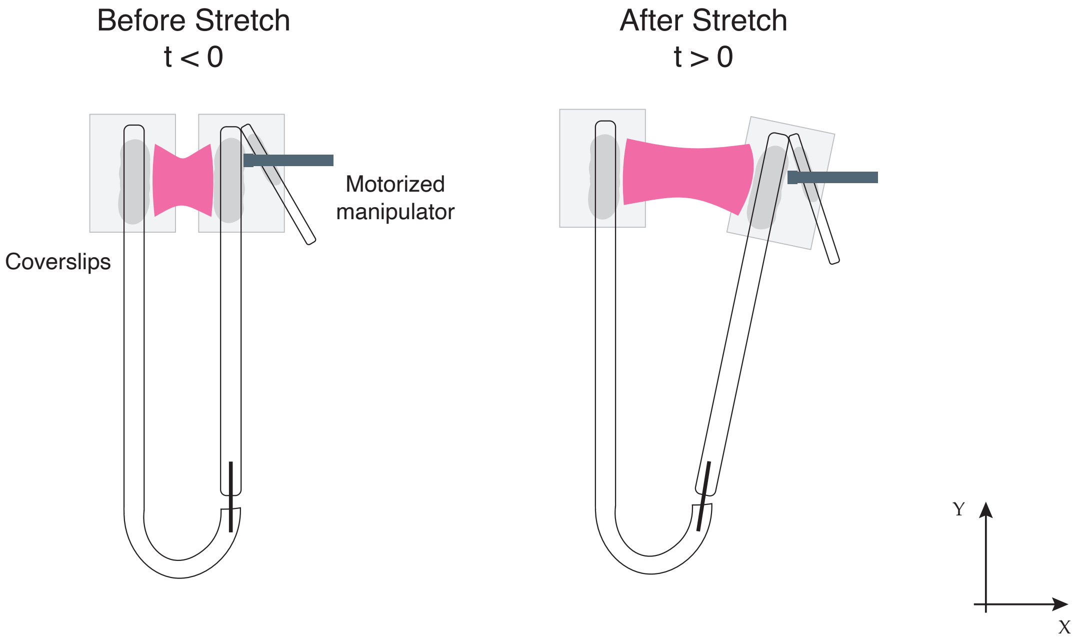

Supplementary Figure 1: Stretching device. Example of a high magnification imaging stretching device. Shaded areas in dark gray depict the UV-glue used to affix the glass capillaries to the coverslips.

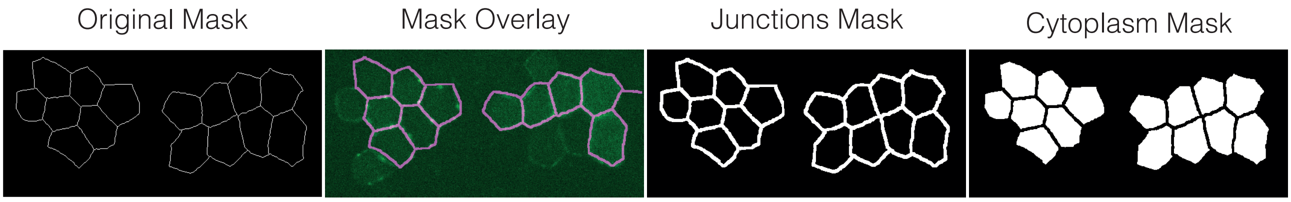

Supplementary Figure 2: Protein enrichment measurements. Processing of segmentation masks to perform fluorescence intensity measurements in either the junctions or the cytoplasm.

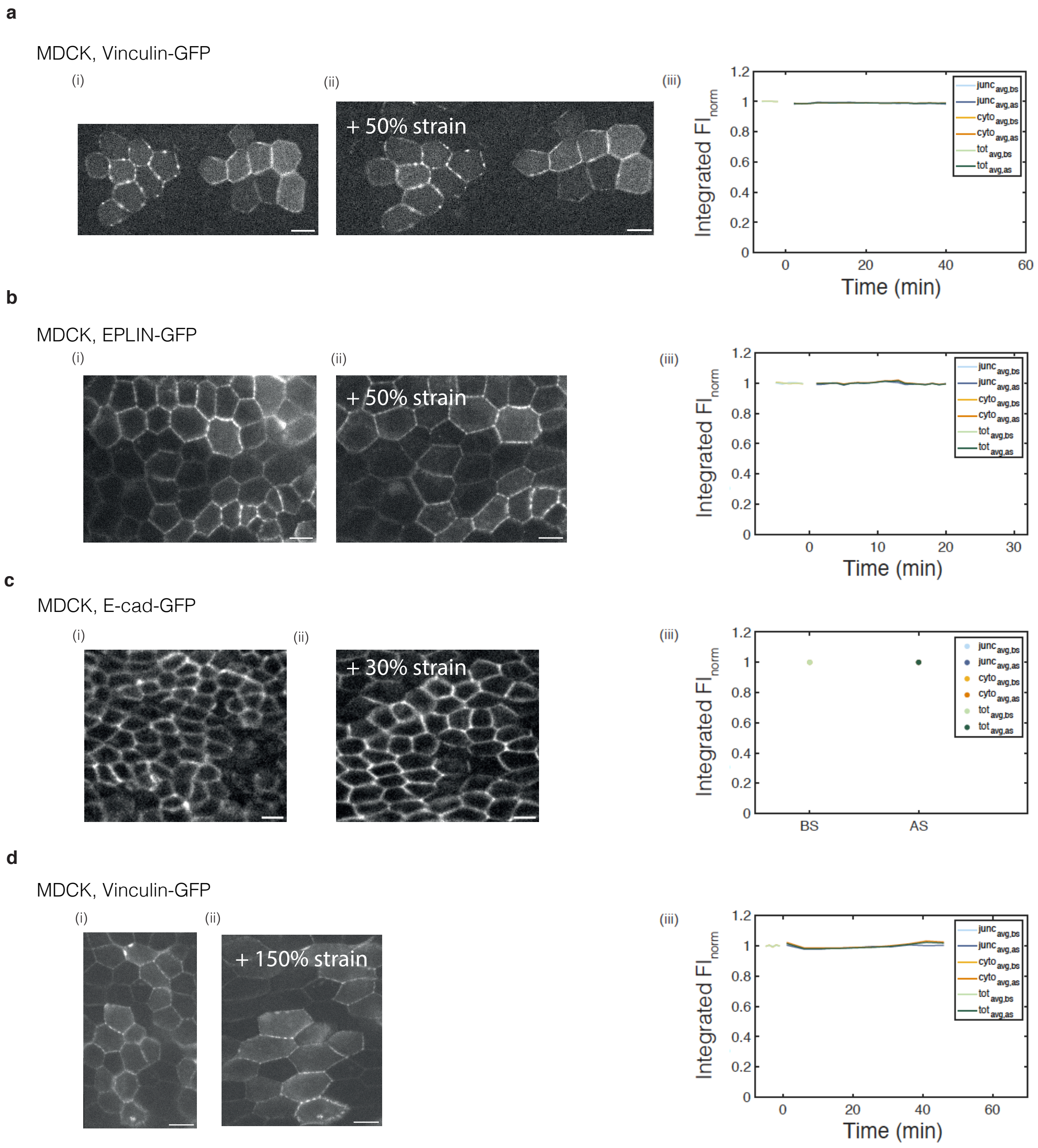

Supplementary Figure 3: Protein enrichment before and during stretch. Single plane confocal time series of different mechanosensitive proteins before and during stretch. Strain is indicated in the top left corner. Scale bars= 10 µm. **(a)** Vinculin-GFP, (i) before and (ii) after 50% strain applied for 40 min. **(b)** EPLIN-GFP, (i) before and (ii) after 50% strain applied for 20 min. **(c)** E-cadherin-GFP, (i) before and (ii) after 30% strain applied for 40 minutes. **(d)** Vinculin-GFP, (i) before and (ii) after 150% strain applied for 40 min. **(a-d)** (iii) Temporal evolution of the average fluorescence intensity of the protein of interest. Images were segmented to measure only junctional fluorescence. Stretch is applied at time 0. In the fluorescence intensity graphs, all time points were normalised to the mean value of the average fluorescence intensity before stretch.

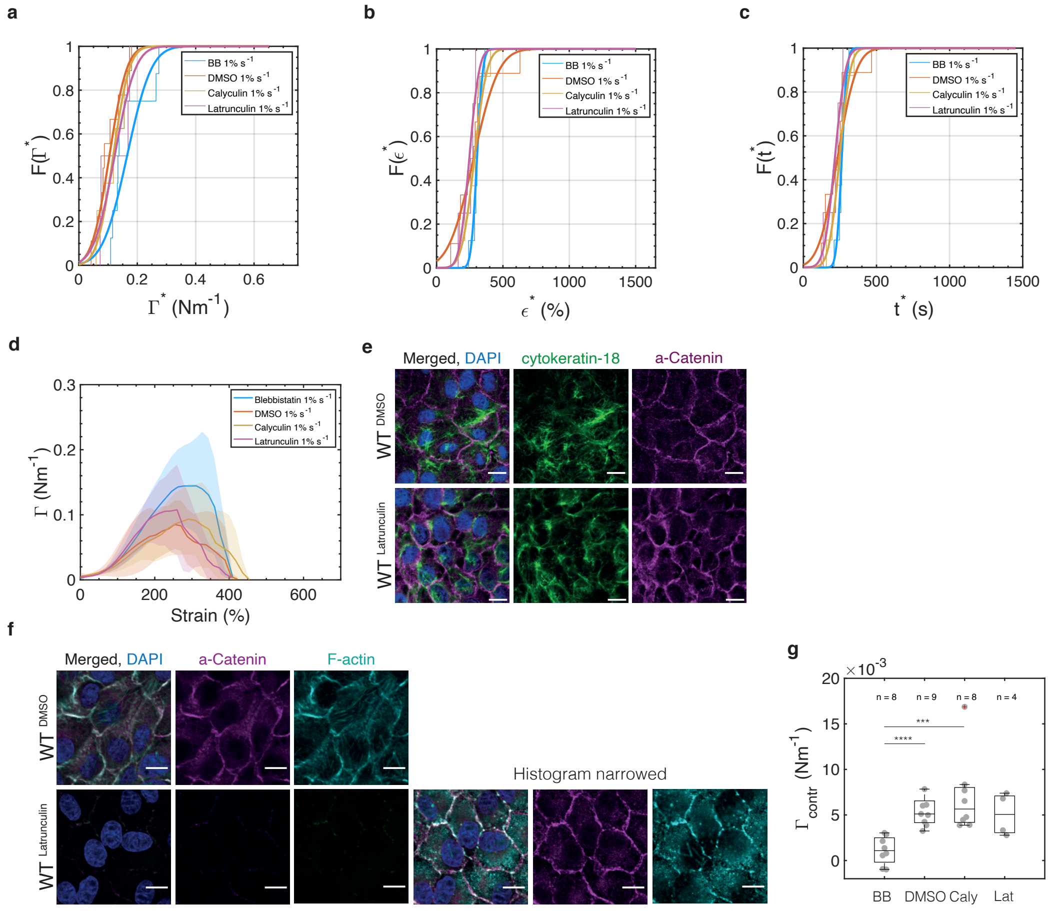

Supplementary Figure 4: Perturbations of the actin cytoskeleton and myosins do not alter keratins. Cumulative distribution functions for the **(a)** rupture tension, **(b)** rupture strain and **(c)** rupture time in monolayers pre-incubated with different treatments perturbing the actomyosin cytoskeleton. Distributions are computed from the box plots shown in **Extended Data Figure 5c-e**. **(d)** Tension versus strain curves for monolayers treated with blebbistatin (blue), DMSO (red), calyculin (yellow), and latrunculin (purple). Monolayers were subjected to ramp experiments performed at 1% s^{-1}. Thick lines indicate the average and the shading indicates the standard deviation. **(a-d, g)** Data was acquired from n = 8 monolayers for blebbistatin, n = 9 for DMSO, n = 8 for calyculin, and n = 4 for latrunculin. **(e-f)** Immunostainings showing the effect of latrunculin 1µM and DMSO. Scale bars, 10 µm. **(e)** Immunostaining against cytokeratin-18 (green) and alpha-catenin (magenta) in WT monolayers treated with DMSO (top row) and latrunculin (bottom row). **(f)** Left: Immunostaining against alpha-Catenin (magenta) and F-actin (cyan) in WT monolayers treated with DMSO (top row) and latrunculin (bottom row). Bottom right: the same images are shown as on the bottom left but with a narrower histogram for alpha-catenin and phalloidin to allow visualisation of the remaining F-actin and alpha-catenin. **(g)** Tension due to contractility in response to different treatments perturbing the actomyosin cytoskeleton. In the box plots, the central mark indicates the median, and the bottom and top edges of the box indicate the 25th and 75th percentiles, respectively. The whiskers extend to the most extreme data points that are not outliers. Data points appear as grey dots. Outliers are indicated with a red '+' symbol. Statistically significant difference: $p = 9\ 10^{-5}$ between BB and DMSO-treated monolayers, and p = 0.00016 between BB and Calyculin-treated monolayers, p = 0.05 between BB and Latrunculin-treated monolayers, p = 0.8 between DMSO and Calyculin-treated monolayers, p = 0.7 between DMSO and Latrunculin-treated monolayers, p = 0.4 between Calyculin and Latrunculin-treated monolayers, two-sided Kolmogorov-Smirnov test.

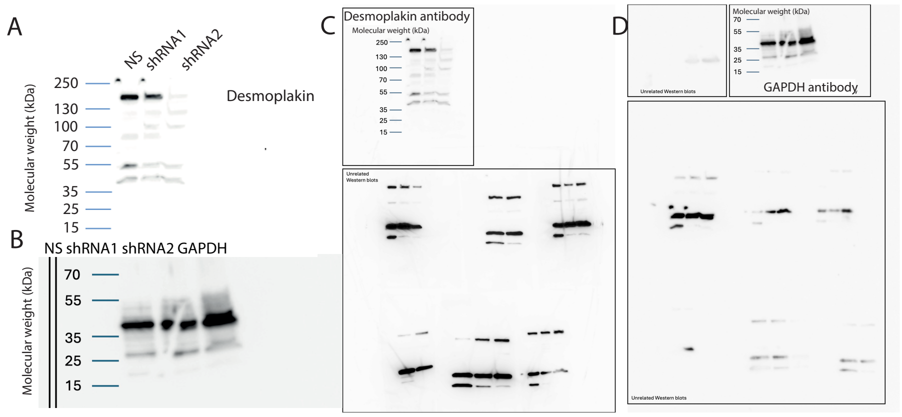

Supplementary Figure 5: Immunoblot of MDCK cells stably expressing non-silencing (NS) and shRNAs targeting desmoplakin. Reduced levels of desmoplakin expression were observed in MDCK cells expressing shRNA2 compared to cells expressing the non-silencing control plasmid pGIPZ. Immunoblot was probed with anti-desmoplakin and anti-GAPDH antibodies. MDCK cells expressing shRNA2 were used in the experiments presented in **Figs 4, 5**, and **Extended Data Figure 6, 7**.

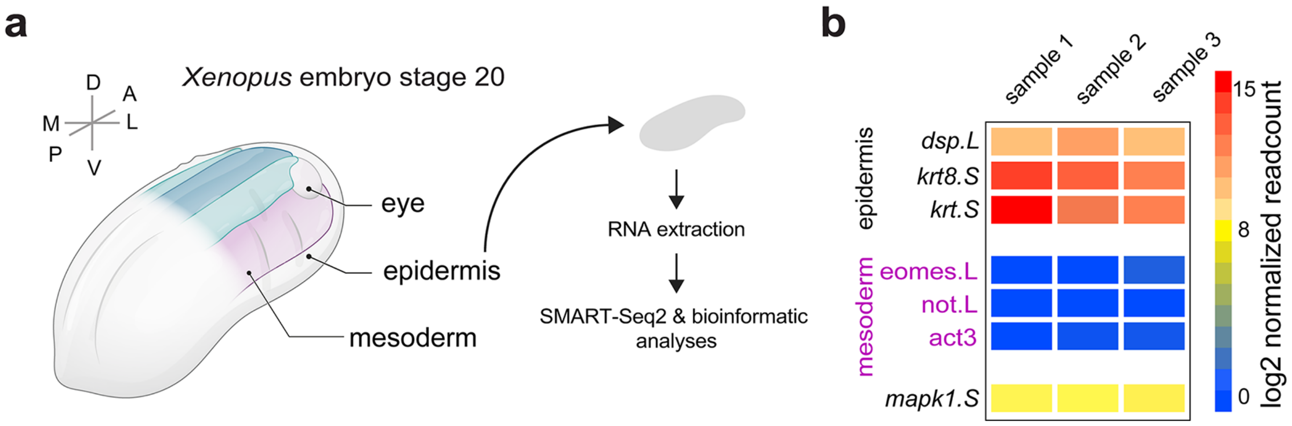

Supplementary Figure 6: Transcriptomics in *Xenopus laevis* embryos. Transcriptomics shows that desmoplakin and keratin 8 are highly expressd in *Xenopus laevis* epidermis. **(a)** Schematic representation of the anatomy of a *Xenopus laevis* embryo. Epidermis was collected from embryos and processed for RNA extraction, SMART-Seq2 and bioinformatics analysis. **(b)** Sample quality control showing that desmoplakin (dsp.L0 and keratin 8 (krt8.S) are enriched in libraries from all epidermis samples. Mesoderm-specific genes (magenta) are present at low levels confirming that most of the cells are epidermal. The final line (mapk1.S) shows a housekeeping gene. Three independent experiments are shown.

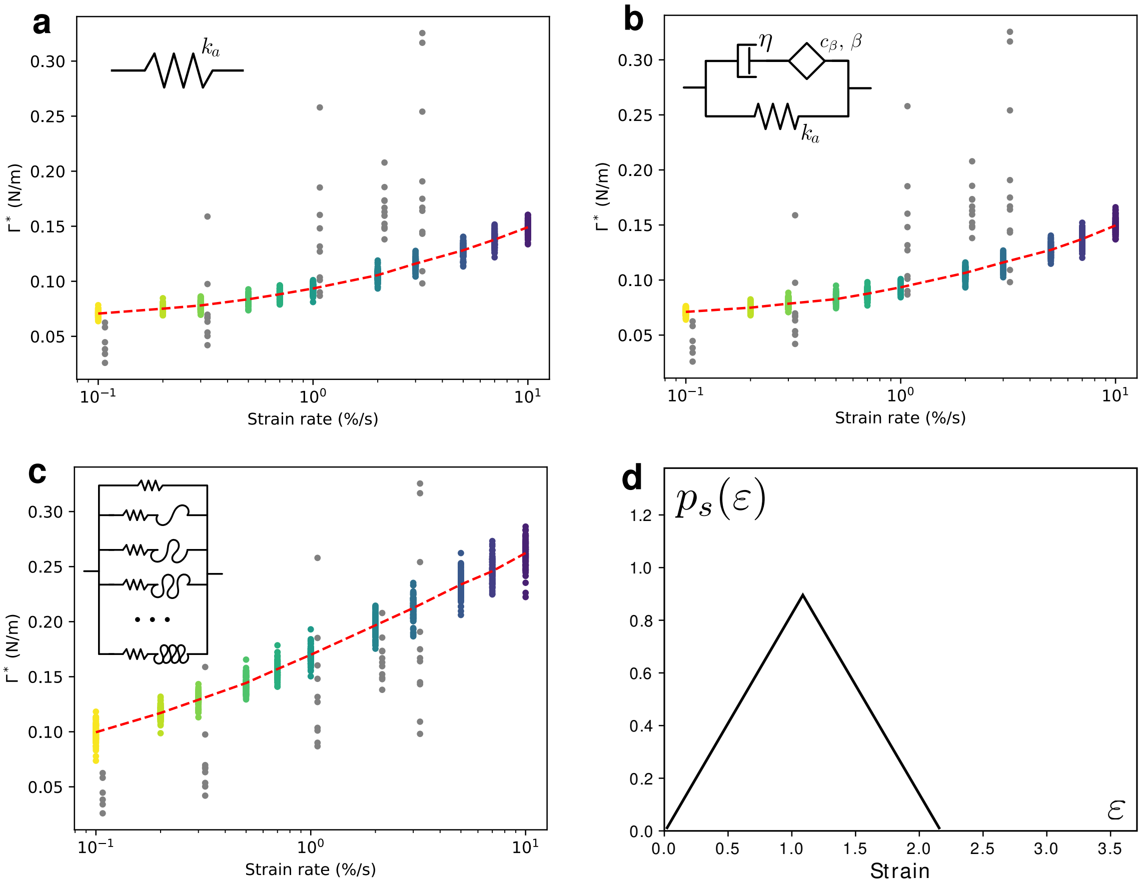

Supplementary Figure 7: Multiscale modelling of fracture onset. Each coloured dot represents a simulation run. 100 simulations were run for each strain rate. Each grey dot represents an experimental data point. The dashed and dotted red lines link the mean value for each strain rate to show the trend. **a - c,** Rupture tension as a function of the strain rate for the three rheological models presented in **Fig. 6 d, e, g** respectively. **d,** Distribution p_s of strains at which keratin bundles are recruited to bear load.

List of Tables

1 Statistical analysis of the rupture tension of monolayers subjected to ramps in deformation at different strain rates (0.1 % s^{-1} - 3 % s^{-1}) . . . 64
2 Statistical analysis of the rupture strain of monolayers subjected to ramps in deformation at different strain rates (0.1 % s^{-1} - 3 % s^{-1}) . . . 65
3 Statistical analysis of the rupture time of monolayers subjected to ramps in deformation at different strain rates (0.1 % s^{-1} - 3 % s^{-1}) . . . 65
4 Statistical analysis of the pre-tension of monolayers subjected to ramps in deformation at different strain rates (0.1 % s^{-1} - 3 % s^{-1}) . . . 65
5 Statistical analysis of the strain stiffening between 15% strain and 120% strain displayed by monolayers subjected to ramps in deformation at different strain rates (0.1 % s^{-1} - 3 % s^{-1}). . . . 66
6 mRNA transcript abundance in wild-type MDCK cells. . . . 66
7 Parameters for the stochastic bond model. . . . 66
8 Parameters for the rheological models shown in **Fig. 6**. . . . 67
9 Table reporting experiment numbers. . . . 72

Statistical Analysis for ramps performed at different strain rates in wild-type monolayers

F^*/w_0	0.1	0.3	1	2	3
0.1	1				
0.3	0.002	1			
1	0.0003	0.013	1		
2	0.0002	0.0002	0.098	1	
3	0.0002	0.006	0.94	0.37	1

Supplementary Table 1: Statistical analysis of the rupture tension of monolayers subjected to ramps in deformation at different strain rates (0.1 % s^{-1} - 3 % s^{-1}). p-values are indicated in the blue shaded area. Statistically significant difference was determined using a two-sided Kolmogorov-Smirnov test: ns P > 0.05, *P < 0.05, **P < 0.01, ***P < 0.001.

ε^*	0.1				
0.1	1	0.3			
0.3	0.14	1	1		
1	0.0014	0.06	1	2	
2	0.0002	0.0013	0.3	1	3
3	9.9e-4	0.006	0.4	0.7	1

Supplementary Table 2: Statistical analysis of the rupture strain of monolayers subjected to ramps in deformation at different strain rates (0.1 % s^{-1} - 3 % s^{-1}). p-values are indicated in the blue shaded area. Statistically significant difference was determined using a two-sided Kolmogorov-Smirnov test: ns $P > 0.05$, $**P < 0.01$.

t^*	0.1				
0.1	1	0.3			
0.3	0.0004	1	1		
1	0.0003	3e-5	1	2	
2	0.0002	2e-5	1.1e-5	1	3
3	0.0002	2e-5	1.1e-5	0.012	1

Supplementary Table 3: Statistical analysis of the rupture time of monolayers subjected to ramps in deformation at different strain rates (0.1 % s^{-1} - 3 % s^{-1}). p-values are indicated in the blue shaded area. Statistically significant difference was determined using a two-sided Kolmogorov-Smirnov test: $*P < 0.05$, $***P < 0.001$, $****P < 0.0001$.

F_0/w_0	0.1				
0.1	1	0.3			
0.3	0.7	1	1		
1	0.16	0.3	1	2	
2	0.012	0.005	0.015	1	3
3	0.5	0.8	0.6	0.012	1

Supplementary Table 4: Statistical analysis of the pre-tension of monolayers subjected to ramps in deformation at different strain rates (0.1 % s^{-1} - 3 % s^{-1}). p-values are indicated in the blue shaded area. Statistically significant difference was determined using a two-sided Kolmogorov-Smirnov test: ns $P > 0.05$, $*P < 0.05$.

Strain Stiffening	0.1				
0.1	1	0.3			
0.3	0.005	1	1		
1	0.016	0.002	1	2	
2	0.003	0.00015	0.003	1	3
3	0.003	0.00015	0.03	0.15	1

Supplementary Table 5: Statistical analysis of the strain stiffening between 15% strain and 120% strain displayed by monolayers subjected to ramps in deformation at different strain rates (0.1 % s^{-1} - 3 % s^{-1}). p-values are indicated in the blue shaded area. Statistically significant difference was determined using a two-sided Kolmogorov-Smirnov test: ns $P > 0.05$, *$P < 0.05$, **$P < 0.01$, ***$P < 0.001$, ****$P < 0.0001$.

Name	Gene	Avg. FPKM
Keratin 8	KRT8	613.2749
Keratin 7	KRT7	382.5791
Keratin 18	KRT18	271.027
Desmoplakin	DSP	55.76
Desmocollin 2	DSC2	88.84
Desmoglein 2	DSG2	137.43
Desmoglein 3	DSG3	16
Desmocollin 3	DSC3	15.56
Cadherin 6	CDH6	132.16
E-cadherin	CDH1	114.12
Cadherin 17	CDH17	47.15
Alpha Catenin	CTNNA1	444.74
Beta Catenin	BCAT1	352.86

Supplementary Table 6: RNA-seq analysis in MDCK cells. The first column indicates the names of the proteins, in the second column appear the gene names, and the third column shows the mRNA abundance obtained by RNA-seq in fragments per kilobase million (FPKM).

Number of bonds, N_{ref}	100
k_{on} $(1/s)$	3e-3
$k_{off,0}$ $(1/s)$	3e-4
f_0 (N)	0.055

Supplementary Table 7: Parameters for the stochastic bond model.

Linear elastic model			
k_a (Pa)	-	-	-
760	-	-	-

Linear viscoelastic model			
k_a(Pa)	c_β (Pa $\cdot$ s$^\beta$)	β	η (Pa $\cdot$ s)
760	1360	0.22	1e4

Nonlinear spring			
k (Pa)	ϵ_{start}	ϵ_{end}	k_a (Pa)
15e3	0	2.2	1e3

Nonlinear viscoelastic model			
τ (s)	ϵ_{start}	ϵ_{end}	k_a (Pa)
55	0	2.2	1e3

Linear viscoelastic model (K14-R125C)			
k_a(Pa)	c_β (Pa $\cdot$ s$^\beta$)	β	η (Pa $\cdot$ s)
1600	1360	0.22	1e4

Supplementary Table 8: Parameters for the rheological models shown in **Fig. 6**.

**Figure**	**Panel**	**Condition (s)**	**Data points**	**Independent days**
Figure 1	d	1%s^{-1}	10	5
Figure 2	d	Calyculin 20nM	7	6
Figure 3	a - e, g - i	• 0.1%s^{-1}<br>• 0.3%s^{-1}<br>• 1%s^{-1}<br>• 2%s^{-1}<br>• 3%s^{-1}	• 6<br>• 9<br>• 10<br>• 11<br>• 11	• 5<br>• 4<br>• 5<br>• 5<br>• 6
Figure 4	c	WT vs K14,R125C - cyto18, DSP	1	2
Figure 4	d	NS vs DSPshRNA - cyto18, aCat	1	2

Figure 4	e, f	• WT 1%s^{-1}<br>• K14,R125C 1%s^{-1}	• 10<br>• 9	• 5<br>• 5
Figure 4	g, h	• NS 1%s^{-1}<br>• DSPshRNA 1%s^{-1}	• 11<br>• 12	• 3<br>• 4
Figure 5	a, b	• WT 1%s^{-1}<br>• K14,R125C 1%s^{-1}	• 10<br>• 9	• 5<br>• 5
Figure 5	c, d	• NS 1%s^{-1}<br>• DSPshRNA 1%s^{-1}	• 11<br>• 12	• 3<br>• 4
Figure 5	e, f	• WT 0.1%s^{-1}<br>• WT 0.3%s^{-1}<br>• WT 1%s^{-1}<br>• WT 2%s^{-1}<br>• WT 3%s^{-1}<br>• K14,R125C 0.3%s^{-1}<br>• K14,R125C 1%s^{-1}	• 6<br>• 9<br>• 10<br>• 11<br>• 11<br>• 8<br>• 9	• 5<br>• 4<br>• 5<br>• 5<br>• 6<br>• 4<br>• 5

Figure 6	d-o	• WT 0.1%s^{-1}<br>• WT 0.3%s^{-1}<br>• WT 1%s^{-1}<br>• WT 2%s^{-1}<br>• WT 3%s^{-1}<br>• K14,R125C 0.3%s^{-1}<br>• K14,R125C 1%s^{-1}<br>• Calyculin 20nM	• 6<br>• 9<br>• 10<br>• 11<br>• 11<br>• 8<br>• 9<br>• 7	• 5<br>• 4<br>• 5<br>• 5<br>• 6<br>• 4<br>• 5<br>• 6
Extended Data Figure 1	a, b	WT 0.1%s^{-1}	10	5
• Extended Data Figure 1<br>• Extended Data Figure 2	• a, b<br>• f	• WT 0.1%s^{-1}<br>• WT 0.3%s^{-1}<br>• WT 1%s^{-1}<br>• WT 2%s^{-1}<br>• WT 3%s^{-1}	• 6<br>• 9<br>• 10<br>• 11<br>• 11	• 5<br>• 4<br>• 5<br>• 5<br>• 6
Extended Data Figure 2	c, d	• WT 1%s^{-1}<br>• K14, R125C 1%s^{-1}	• 10<br>• 9	• 5<br>• 5
Extended Data Figure 3	a, c, d	Calyculin 20nM	7	6
Extended Data Figure 3	b, c, e	BB, Calyculin 20nM	6	3

Extended Data Figure 3	g	WT, DMSO vs Calyculin - ECad, F-actin	7	3
Extended Data Figure 3	h	WT, DMSO vs Calyculin - pMyo, F-actin	9	3
Extended Data Figure 3	i	WT, DMSO vs Calyculin - cyto18, aCat	5	2
Extended Data Figure 4	a - i	• WT 0.1%s^{-1}<br>• WT 0.3%s^{-1}<br>• WT 1%s^{-1}<br>• WT 2%s^{-1}<br>• WT 3%s^{-1}<br>• Calyculin 20nM	• 6<br>• 9<br>• 10<br>• 11<br>• 11<br>• 7	• 5<br>• 4<br>• 5<br>• 5<br>• 6<br>• 6
Extended Data Figure 5	a	• WT 0.1%s^{-1}<br>• WT 0.3%s^{-1}<br>• WT 1%s^{-1}<br>• WT 2%s^{-1}<br>• WT 3%s^{-1}<br>• BB 50uM<br>• DMSO<br>• Calyculin 20nM<br>• Lat 1uM	• 6<br>• 9<br>• 10<br>• 11<br>• 11<br>• 8<br>• 9<br>• 7<br>• 4	• 5<br>• 4<br>• 5<br>• 5<br>• 6<br>• 7<br>• 7<br>• 6<br>• 3
Extended Data Figure 6	b, f, g, j	• WT 1%s^{-1}<br>• K14,R125C 1%s^{-1}	• 10<br>• 9	• 5<br>• 5

Extended Data Figure 6	c, h, i	• NS $1\%s^{-1}$<br>• DSPshRNA $1\%s^{-1}$	• 11<br>• 12	• 3<br>• 4
Extended Data Figure 6	c	WT vs K14,R125C - pMyo, F-actin	2	1
Extended Data Figure 6	e	NS vs DSPshRNA - pMyo, F-actin	6	2
Extended Data Figure 7	a - d, h - j	• K14,R125C $0.3\%s^{-1}$<br>• K14,R125C $1\%s^{-1}$<br>• WT $1\%s^{-1}$	• 8<br>• 9<br>• 10	• 4<br>• 5<br>• 5
Extended Data Figure 7	e	K14,R125C - cyto18, E-cad	3	1
Extended Data Figure 7	f	WT vs K14,R125C - cyto18, aCat	2	1
Extended Data Figure 7	g	NS vs DSPshRNA - E-cad, F-actin	6	3
Extended Data Figure 8	d	• Control, early<br>• Control, late<br>• DSP-MO * krt8 -MO, late	• 609<br>• 579<br>• 784	• 3<br>• 3<br>• 3
Extended Data Figure 8	e	• Control<br>• MO	• 30<br>• 30	• 3<br>• 3

Extended Data Figure 8	f	• Control, early<br>• Control, late<br>• MO, late	• 12<br>• 12<br>• 12	• 3<br>• 3<br>• 2
Extended Data Figure 9	a, b	• WT<br>• K14,R125C	• 1<br>• 1	• 1<br>• 1
Extended Data Figure 10	b - k	• WT 0.1%s^{-1}<br>• WT 0.3%s^{-1}<br>• WT 1%s^{-1}<br>• WT 2%s^{-1}<br>• WT 3%s^{-1}<br>• K14,R125C 0.3%s^{-1}<br>• K14,R125C 1%s^{-1}<br>• Calyculin 20nM	• 6<br>• 9<br>• 10<br>• 11<br>• 11<br>• 8<br>• 9<br>• 7	• 5<br>• 4<br>• 5<br>• 5<br>• 6<br>• 7<br>• 7<br>• 6
Supplementary Figure 4	e	WT, DMSO vs Lat - cyto18, aCat	1	3
Supplementary Figure 4	f	WT, DMSO vs Lat - aCat, F-actin	1	3

Supplementary Table 9: Table reporting experiment numbers. Number of data points collected, and number of independent days for each experiment in every condition.

[48] Wangsun Choi, Bipul R Acharya, Gr´egoire Peyret, Marc-Antoine Fardin, Ren´e-Marc M`ege,
Benoit Ladoux, Alpha S Yap, Alan S Fanning, and Mark Peifer. Remodeling the zonula adherens in response to tension and the role of afadin in this response. Journal of Cell Biology, 213(2):243–260, 2016.

[49] Xiaoguang Chen and G Wayne Brodland. Multi-scale finite element modeling allows the mechanics of amphibian neurulation to be elucidated. Physical Biology, 5(1):015003, 2008.
35

[50] Antone G Jacobson and Richard Gordon. Changes in the shape of the developing vertebrate
nervous system analyzed experimentally, mathematically and by computer simulation. Journal of Experimental Zoology, 197(2):191–246, 1976.

[51] Charl`ene Guillot and Thomas Lecuit. Adhesion disengagement uncouples intrinsic and extrinsic
forces to drive cytokinesis in epithelial tissues. Developmental cell, 24(3):227–241, 2013.

[52] Carl-Philipp Heisenberg and Yohanns Bella¨ıche. Forces in tissue morphogenesis and patterning.
Cell, 153(5):948–962, 2013.

[53] PR Onck, T Koeman, T Van Dillen, and Erik van der Giessen. Alternative explanation of sti↵ening in cross-linked semiflexible networks. Physical review letters, 95(17):178102, 2005.

[54] David A Head, Alex J Levine, and FC MacKintosh. Deformation of cross-linked semiflexible
polymer networks. Physical review letters, 91(10):108102, 2003.

[55] Roy A Quinlan, Nicole Schwarz, Reinhard Windo↵er, Christine Richardson, Tim Hawkins, Joshua A Broussard, Kathleen J Green, and Rudolf E Leube. A rim-and-spoke hypothesis to explain the biomechanical roles for cytoplasmic intermediate filament networks. Journal of cell science, 130(20):3437–3445, 2017.

[56] Jens-Friedrich Nolting, Wiebke M¨obius, and Sarah K¨oster. Mechanics of individual keratin
bundles in living cells. Biophysical journal, 107(11):2693–2699, 2014.

[57] Elisabeth Fischer-Friedrich, Yusuke Toyoda, Cedric J Cattin, Daniel J M¨uller, Anthony A Hyman, and Frank J¨ulicher. Rheology of the active cell cortex in mitosis. Biophysical journal, 111(3):589–600, 2016.

[58] Reinhard Windo↲er, Monika Borchert-Stuhltr¨ager, and Rudolf E Leube. Desmosomes: interconnected
calcium-dependent structures of remarkable stability with significant integral
membrane protein turnover. Journal of cell science, 115(8):1717–1732, 2002.

[59] Minnah Thomas, Benoit Ladoux, and Yusuke Toyama. Desmosomal junctions govern tissue
integrity and actomyosin contractility in apoptotic cell extrusion. Current Biology, 30(4):682–690, 2020.

[60] Judith B F¨ulle, Henri Huppert, David Liebl, Jaron Liu, Rogerio Alves de Almeida, Bian Yanes,
Graham D Wright, E Birgitte Lane, David R Garrod, and Christoph Ballestrem. Desmosome dualism–most of the junction is stable, but a plakophilin moiety is persistently dynamic. Journal
of cell science, 134(21):jcs258906, 2021.

[61] Kate E Cavanaugh, Michael F Staddon, Theresa A Chmiel, Robert Harmon, Srikanth Budnar,
Shiladitya Banerjee, Margaret L Gardel, et al. Force-dependent intercellular adhesion strengthening
underlies asymmetric adherens junction contraction. Current Biology, 32(9):1986–2000, 2022.

[62] Jana Slov´akov´a, Mateusz Sikora, Feyza Nur Arslan, Silvia Caballero-Mancebo, SF Gabriel Krens, Walter A Kaufmann, Jack Merrin, and Carl-Philipp Heisenberg. Tension-dependent

stabilization of e-cadherin limits cell–cell contact expansion in zebrafish germ-layer progenitor
cells. Proceedings of the National Academy of Sciences, 119(8):e2122030119, 2022.

[63] Craig D Buckley, Jiongyi Tan, Karen L Anderson, Dorit Hanein, Niels Volkmann, William I Weis, W James Nelson, and Alexander R Dunn. The minimal cadherin-catenin complex binds to actin filaments under force. Science, 346(6209):1254211, 2014.

[64] Masatoshi Takeichi. Dynamic contacts: rearranging adherens junctions to drive epithelial remodelling. Nature reviews Molecular cell biology, 15(6):397–410, 2014.
