# Supplementary material for: Rupture strength of living cell monolayers
Source: Nat Mater. 2024 Oct 28;23(11):1563–74. doi: 10.1038/s41563-024-02027-3 (PMC11525174; doi:10.1038/s41563-024-02027-3)
Supplement: Supplementary file 2 — Reporting Summary [file 41563_2024_2027_MOESM2_ESM.pdf]

nature portfolio

Corresponding author(s): Julia Duque, Alexandre Kabla, Guillaume Charras

Last updated by author(s): 26/01/2024

Reporting Summary

Nature Portfolio wishes to improve the reproducibility of the work that we publish. This form provides structure for consistency and transparency in reporting. For further information on Nature Portfolio policies, see our Editorial Policies and the Editorial Policy Checklist.

Please do not complete any field with "not applicable" or n/a. Refer to the help text for what text to use if an item is not relevant to your study.
For final submission: please carefully check your responses for accuracy; you will not be able to make changes later.

Statistics

For all statistical analyses, confirm that the following items are present in the figure legend, table legend, main text, or Methods section.

n/a	Confirmed	
☐	☑	The exact sample size (*n*) for each experimental group/condition, given as a discrete number and unit of measurement
☐	☑	A statement on whether measurements were taken from distinct samples or whether the same sample was measured repeatedly
☐	☑	The statistical test(s) used AND whether they are one- or two-sided<br>*Only common tests should be described solely by name; describe more complex techniques in the Methods section.*
☑	☐	A description of all covariates tested
☐	☑	A description of any assumptions or corrections, such as tests of normality and adjustment for multiple comparisons
☐	☑	A full description of the statistical parameters including central tendency (e.g. means) or other basic estimates (e.g. regression coefficient) AND variation (e.g. standard deviation) or associated estimates of uncertainty (e.g. confidence intervals)
☐	☑	For null hypothesis testing, the test statistic (e.g. *F*, *t*, *r*) with confidence intervals, effect sizes, degrees of freedom and *P* value noted<br>*Give P values as exact values whenever suitable.*
☑	☐	For Bayesian analysis, information on the choice of priors and Markov chain Monte Carlo settings
☑	☐	For hierarchical and complex designs, identification of the appropriate level for tests and full reporting of outcomes
☑	☐	Estimates of effect sizes (e.g. Cohen's *d*, Pearson's *r*), indicating how they were calculated

Our web collection on statistics for biologists contains articles on many of the points above.

Software and code

Policy information about availability of computer code

Data collection	Confocal microscopy images were acquired using Olympus FV10 software or Micromanager. Force measurements were acquired using LabView Virtual Instrument to control the data acquistion system (USB-1608G, Measurement Computing) and the motorised stage (MG126.DG1 motorised stage with Mercury DC motor controller, Physik Instrumente)
Data analysis	Routine image processing was carried out in Fiji. More complex processing and all of the mechanical data processing was carried out in Matlab R2019a. The computational models were implemented in the Julia programming language.

For manuscripts utilizing custom algorithms or software that are central to the research but not yet described in published literature, software must be made available to editors and reviewers. We strongly encourage code deposition in a community repository (e.g. GitHub). See the Nature Portfolio guidelines for submitting code & software for further information.

Data

Policy information about availability of data

All manuscripts must include a data availability statement. This statement should provide the following information, where applicable:

- Accession codes, unique identifiers, or web links for publicly available datasets
- A description of any restrictions on data availability
- For clinical datasets or third party data, please ensure that the statement adheres to our policy

All data supporting the conclusions are available from the UCL data repository (https://rdr.ucl.ac.uk/) with a unique link: https://doi.org/10.5522/04/21407160.

Research involving human participants, their data, or biological material

Policy information about studies with human participants or human data. See also policy information about sex, gender (identity/presentation), and sexual orientation and race, ethnicity and racism.

Reporting on sex and gender	n/a
Reporting on race, ethnicity, or other socially relevant groupings	n/a
Population characteristics	n/a
Recruitment	n/a
Ethics oversight	n/a

Note that full information on the approval of the study protocol must also be provided in the manuscript.

Field-specific reporting

Please select the one below that is the best fit for your research. If you are not sure, read the appropriate sections before making your selection.

☑ Life sciences ☐ Behavioural & social sciences ☐ Ecological, evolutionary & environmental sciences

For a reference copy of the document with all sections, see nature.com/documents/nr-reporting-summary-flat.pdf

Life sciences study design

All studies must disclose on these points even when the disclosure is negative.

Sample size	Each dataset is pooled across experiments performed on at least 3 independent days. Sample size is provided for each experiment. Samples sizes were chosen such that diffrences larger than 50% could be identified reliab
Data exclusions	Monolayers in which the first crack arose from a detachment of the bar were excluded, Outliers were defind as experiments that fell outside 1.5 interquartile ranges above the upper quartile (75%) or below the lower quartile (25%). The reported n numbers are the numbers of data points included in the statistical analysis after any data exclusion)
Replication	All experiments performed succesfully on at last 3 different days.
Randomization	On each day of experiment, care was take to perform control and perturbation experiments in a randomised order
Blinding	Blinding was not performed since quantifications were obtained through automated algorithms and therefore not susceptible to experimenter bias.

Behavioural & social sciences study design

All studies must disclose on these points even when the disclosure is negative.

Study description	
Research sample	
Sampling strategy	
Data collection	
Timing	
Data exclusions	
Non-participation	
Randomization	

Ecological, evolutionary & environmental sciences study design

All studies must disclose on these points even when the disclosure is negative.

Study description

Research sample

Sampling strategy

Data collection

Timing and spatial scale

Data exclusions

Reproducibility

Randomization

Blinding

Did the study involve field work? ☐ Yes ☐ No

Field work, collection and transport

Field conditions

Location

Access & import/export

Disturbance

Reporting for specific materials, systems and methods

We require information from authors about some types of materials, experimental systems and methods used in many studies. Here, indicate whether each material, system or method listed is relevant to your study. If you are not sure if a list item applies to your research, read the appropriate section before selecting a response.

Materials & experimental systems

n/a	Involved in the study	
☐	☑	Antibodies
☐	☑	Eukaryotic cell lines
☑	☐	Palaeontology and archaeology
☐	☑	Animals and other organisms
☑	☐	Clinical data
☑	☐	Dual use research of concern
☑	☐	Plants

Methods

n/a	Involved in the study	
☑	☐	ChIP-seq
☑	☐	Flow cytometry
☑	☐	MRI-based neuroimaging

Antibodies

Antibodies used

Mouse anti phospho myosin light chain 2 S19 (cell signalling 3675S), mouse anti-Ecadherin (BD Bioscience 610181), mouse anti-keratin18 (Abcam ab668), Rabbit anti-alpha-catenin (Sigma-Aldrich C-2081), rabbit anti-desmoplakin (Abcam, ab71690), mouse anti-GAPDH (Novus biologicals, NB300-221), mouse anti-desmoplakin 1/2 (Progen 65146), mouse anti-GAPDH (abcam, ab8245), mouse IgG HRP linked antibodies (Cytiva, NXA931).

Secondary antibodies:
Goat anti-mouse Alexa 568, Life Technologies, A11031
Goat anti-rabbit Alexa-647, Life Technologies, A27040

Validation

-mouse anti-GAPDH was validated for cross-reactivity with canine protein in Khalilgharibi et al, Nature physics, 2019
-rabbit anti-desmoplakin and mouse anti-keratin18 were validated by Western blotting and verifying that they recognised a protein of the correct molecular weight. We also verified that the localisation of the antigen in immunofluorescence experiments matched that in previous reports.
-rabbit anti-alpha catenin: the manufacturer indicates cross reactivity with several mammalian species. In immuno-fluorescence experiments, the antibody recognises an antigen localised to intercellular junctions as expected.
-mouse anti-phosphomyosin light chain 2 S19: the manufacturer indicates cross reactivity with canine protein.
-mouse anti-ECadherin: the manufacturer indiates cross-reactivity against canine protein.

Eukaryotic cell lines

Policy information about cell lines and Sex and Gender in Research

Cell line source(s)	MDCK II cells were obtained from Prof. Yasuyuki Fujita, Kyoto university, Japan. These can be obtained commercially from ECACC, catalogno: 00062107.
Authentication	The cell line was not authenticated for this study. RNA sequencing data provuded in this study indicates canine origin.
Mycoplasma contamination	All cell lines were tested negative for mycoplasma contamination. Tests were conducted using MycoAlert PLUS detection kit (Lonza, cat. no. LT07-710)
Commonly misidentified lines (See ICLAC register)	No commonly misidentified cell lines were used.

Palaeontology and Archaeology

Specimen provenance	
Specimen deposition	
Dating methods	

☐ Tick this box to confirm that the raw and calibrated dates are available in the paper or in Supplementary Information.

Ethics oversight	

Note that full information on the approval of the study protocol must also be provided in the manuscript.

Animals and other research organisms

Policy information about studies involving animals; ARRIVE guidelines recommended for reporting animal research, and Sex and Gender in Research

Laboratory animals	One day old Xenopus Laevis embryos.
Wild animals	no wild animals were used in this study.
Reporting on sex	n/a
Field-collected samples	No samples were collected in the field.
Ethics oversight	Animal procedures were approved by the Ethics Committee and Animal Welfare Body (ORBEA) of the Instituto Gulbenkian de Ciencia, Portugal, and complied with the Portuguese (Decreto'Leo n113/2013) and European (Directive 2010/63/EU) legislations.

Note that full information on the approval of the study protocol must also be provided in the manuscript.

Clinical data

Policy information about clinical studies

All manuscripts should comply with the ICMJE guidelines for publication of clinical research and a completed CONSORT checklist must be included with all submissions.

Clinical trial registration	n/a
Study protocol	n/a
Data collection	n/a
Outcomes	n/a

Dual use research of concern

Policy information about dual use research of concern

Hazards

Could the accidental, deliberate or reckless misuse of agents or technologies generated in the work, or the application of information presented in the manuscript, pose a threat to:

No	Yes	
☑	☐	Public health
☑	☐	National security
☑	☐	Crops and/or livestock
☑	☐	Ecosystems
☑	☐	Any other significant area

Experiments of concern

Does the work involve any of these experiments of concern:

No	Yes	
☑	☐	Demonstrate how to render a vaccine ineffective
☑	☐	Confer resistance to therapeutically useful antibiotics or antiviral agents
☑	☐	Enhance the virulence of a pathogen or render a nonpathogen virulent
☑	☐	Increase transmissibility of a pathogen
☑	☐	Alter the host range of a pathogen
☑	☐	Enable evasion of diagnostic/detection modalities
☑	☐	Enable the weaponization of a biological agent or toxin
☑	☐	Any other potentially harmful combination of experiments and agents

Plants

Seed stocks

Novel plant genotypes

Authentication

ChIP-seq

Data deposition

☐ Confirm that both raw and final processed data have been deposited in a public database such as GEO.

☐ Confirm that you have deposited or provided access to graph files (e.g. BED files) for the called peaks.

Data access links
May remain private before publication.

Files in database submission

Genome browser session
(e.g. UCSC)

Methodology

Replicates

Sequencing depth

Antibodies

Peak calling parameters

Data quality

Software

Flow Cytometry

Plots

Confirm that:

- [ ] The axis labels state the marker and fluorochrome used (e.g. CD4-FITC).
- [ ] The axis scales are clearly visible. Include numbers along axes only for bottom left plot of group (a 'group' is an analysis of identical markers).
- [ ] All plots are contour plots with outliers or pseudocolor plots.
- [ ] A numerical value for number of cells or percentage (with statistics) is provided.

Methodology

Sample preparation

Instrument

Software

Cell population abundance

Gating strategy

- [ ] Tick this box to confirm that a figure exemplifying the gating strategy is provided in the Supplementary Information.

Magnetic resonance imaging

Experimental design

Design type

Design specifications

Behavioral performance measures

Imaging type(s)

Field strength

Sequence & imaging parameters

Area of acquisition

Diffusion MRI ☐ Used ☐ Not used

Preprocessing

Preprocessing software

Normalization

Normalization template

Noise and artifact removal

Volume censoring

Statistical modeling & inference

Model type and settings

Effect(s) tested

Specify type of analysis: ☐ Whole brain ☐ ROI-based ☐ Both

Statistic type for inference

(See Eklund et al. 2016)

Correction

Models & analysis

n/a	Involved in the study
☐	☐ Functional and/or effective connectivity
☐	☐ Graph analysis
☐	☐ Multivariate modeling or predictive analysis

Functional and/or effective connectivity

Graph analysis

Multivariate modeling and predictive analysis

This checklist template is licensed under a Creative Commons Attribution 4.0 International License, which permits use, sharing, adaptation, distribution and reproduction in any medium or format, as long as you give appropriate credit to the original author(s) and the source, provide a link to the Creative Commons license, and indicate if changes were made. The images or other third party material in this article are included in the article's Creative Commons license, unless indicated otherwise in a credit line to the material. If material is not included in the article's Creative Commons license and your intended use is not permitted by statutory regulation or exceeds the permitted use, you will need to obtain permission directly from the copyright holder. To view a copy of this license, visit http://creativecommons.org/licenses/by/4.0/
