# Supplementary material for: Rupture strength of living cell monolayers
Source: Nat Mater. 2024 Oct 28;23(11):1563–74. doi: 10.1038/s41563-024-02027-3 (PMC11525174; doi:10.1038/s41563-024-02027-3)
Supplement: Supplementary file 22 — Results of the computational simulations plotted in Fig. 6 and link to the UCL repository. [file 41563_2024_2027_MOESM22_ESM.pdf]

This archive contains the results of the computational simulations plotted in:
- Figure 6
- Extended Data Figure 10
- Supplementary Figure 7

Code and primary data are available from the UCL data repository(https://rdr.ucl.ac.uk/) with a unique link:
https://doi.org/10.5522/04/21407160
=====================

The subfolders are organized as follow:

- rheology_data:

It contains the stress-strain-time curves for the different rheological models considered in the paper.
The files contain 4 columns that are respectively [time, strain, stress, gradient]. Each file name ends with a block of digits indicating the strain rate in %/s times 100. For instance, stress_strain_spring_70.0.txt corresponds to a strain rage of 0.7%/s.

The specific values of the rheological parameters are available in Supplementary Table 8. The distribution of slack distances is defined on Supp Fig 7d.

The curves for the fractional viscoelastic model have been calculated using RHEOS (https://github.com/JuliaRheology/RHEOS.jl)

The curves for the nonlinear models have been calculated using the generic code available in the repository https://github.com/ComputationalMechanobiology/IFTissueRheol. A simplified sample code to generate the relevant stress-strain curves is also included in the folder.

- adhesion_model_data:

Each file contains, for a given rheological model, i.e. stress-strain curve and strain rate, a list of values for [rupture time, strain, stress], each obtained by running the stochastic rupture model. Except for the constant stress data, each file name ends with a block of digits indicating the strain rate in %/s times 100. For instance, stress_strain_spring_70.0.txt corresponds to a strain rage of 0.7%/s. For the constant stress data, the filename refers to the tension as defined in the paper.

The data has been calculated using the adhesion stochastic model available in the repository https://github.com/ComputationalMechanobiology/CellAdhesion.jl (commitment ID: 8cc2ea2e0b7eafb1e46ca7f279617450b3aaa4e6).

The model parameters are available in Supplementary Table 7.

- adhesion_model_extensions

The folder rupture_nonlinear_model_N_kon_fe_variations contains rupture data, same format as above, but when adhesion model parameters are varied compared to the reference values in Supplementary Table 7: half N, half fe, or 10x the rate constants.

The foldel rupture_Ns contains the rupture stress values obtained by running the stochastic model on each of the experimental curves (copied in the experimental data subfolder), for the value of N adjusted to have a 50% chance of rupture before the experimental value of Gamma* is reached, i.e. the value of N that would cause the adhesion model to break statistically at the same time as the experimental data. The text file fitted_N.txt contains the values of N that were needed for each sample, grouped by strain rates and treatments.

The folder rupture_fracVisco_K14 contains the rupture stress and rupture strain values obtained by running the adjusted adhesion model for K14 mutants, applied to ramp of deformation on the linear fractional model validated for small deformations. The parameters for fractional model for K14 are (η = 1e4, c_β = 1360, β = 0.22, k = 1600).

===================

How the figures relate to this data:

Figure 6

Panel (b-c): The raw data is available in adhesion_model_data/rupture_constant_stress. The stress value is in the file name.

Panel (d): The raw data is available in adhesion_model_data/rupture_linearSpring. The strain rate value is in the file name. Rupture statistics was obtained by running the stochastic bond model on the rheological model data available in rheology_data/time_stress_strain_linearSpring. The experimental data comes from figure 3.

Panel (e): The raw data is available in adhesion_model_data/rupture_fracVisco. The strain rate value is in the file name. Rupture statistics was obtained by running the stochastic bond model on the rheological model data available in rheology_data/time_stress_strain_fracVisco/. The experimental data comes from figure 3.

Panels (f-g): The raw data is available in adhesion_model_data/rupture_nonlinear_spring. The strain rate value is in the file name. Rupture statistics was obtained by running the stochastic bond model on the rheological model data available in rheology_data/time_stress_strain_linearSpring, also providing the strain-strain curve on panel f. The experimental data comes from figure 3.

Panels (h-m): The raw data is available in adhesion_model_data/rupture_nonlinear_maxwell_55. The strain rate value is in the file name. Rupture statistics was obtained by running the stochastic bond model on the rheological model data available in rheology_data/time_stress_strain_nonlinear_maxwell_55. This rheological data also provides the stress-strain curves on panels h and i. The experimental data comes from figure 3.

Panel (n): The fitted values of N for each type of tissue and strain rate are listed in the file fitted_N.txt in the foldel rupture_Ns. The stochastic models were running on experimental stress-strain curves included in the same folder, and also visible on Extended Data Fig 4h (WT and calyculin).

Panel (o): The simulated rupture data is available in the folder adhesion_model_extensions/rupture_fracVisco_K14.

Extended Data Figure 10

Panel (a): The rupture data for each stochastic simulation are in the folder adhesion_model_data/rupture_nonlinear_model_N_kon_fe_variations, for each of the variations of the model parameters. The control data is extracted from adhesion_model_data/rupture_nonlinear_maxwell_55 for 1%/s rate of deformation. All models were using the rheological data of the nonlinear Maxwell model at the strain rate 1%/s from rheology_data/time_stress_strain_nonlinear_maxwell_55.

Panels (b-f): The distributions of rupture stresses are included in adhesion_model_extensions/rupture_Ns. The values of N leading to these distributions are in the fitted_N.txt file.

Panel (g): The raw vaules for N are present in the adhesion_model_extensions/rupture_Ns/fitted_N.txt file.

Panels (h-j): The distributions of rupture stresses are included in

adhesion_model_extensions/rupture_Ns. The values of N leading to these distributions are in the fitted_N.txt file.

Panel (k): The raw vaules for N are present in the adhesion_model_extensions/rupture_Ns/fitted_N.txt file.

Supp Fig 7

Panel a: same as Fig 6d but showing rupture tension rather than rupture strain.
Panel b: same as Fig 6e but showing rupture tension rather than rupture strain.
Panel c: same as Fig 6g but showing rupture tension rather than rupture strain.
